# Changes in memory and cognition during the SARS-CoV-2 human challenge study

**DOI:** 10.1016/j.eclinm.2024.102842

**Published:** 2024-09-21

**Authors:** William Trender, Peter J. Hellyer, Ben Killingley, Mariya Kalinova, Alex J. Mann, Andrew P. Catchpole, David Menon, Edward Needham, Ryan Thwaites, Christopher Chiu, Gregory Scott, Adam Hampshire

**Affiliations:** a Department of Brain Sciences, Imperial College London, UK; b Institute of Psychiatry, Psychology and Neuroscience, King's College London, London, UK; c Cambridge University Hospitals National Health Service Foundation Trust, UK; d Department of Infectious Disease, Imperial College London, London, UK; e National Heart and Lung Institute, Imperial College London, London, UK; f Department of Infectious Diseases, University College London Hospital, London, UK; g HVIVO Services Ltd., London, UK

**Keywords:** COVID-19, SARS-CoV-2, Human challenge study, Cognition, Baseline corrected

## Abstract

**Background:**

Patient-reported outcomes and cross-sectional evidence show an association between COVID-19 and persistent cognitive problems. The causal basis, longevity and domain specificity of this association is unclear due to population variability in baseline cognitive abilities, vulnerabilities, virus variants, vaccination status and treatment.

**Methods:**

Thirty-four young, healthy, seronegative volunteers were inoculated with Wildtype SARS-CoV-2 under prospectively controlled conditions. Volunteers completed daily physiological measurements and computerised cognitive tasks during quarantine and follow-up at 30, 90, 180, 270, and 360 days. Linear modelling examined differences between ‘infected’ and ‘inoculated but uninfected’ individuals. The main cognitive endpoint was the baseline corrected global cognitive composite score across the battery of tasks administered to the volunteers. Exploratory cognitive endpoints included baseline corrected scores from individual tasks. The study was registered on ClinicalTrials.gov with the identifier NCT04865237 and took place between March 2021 and July 2022.

**Findings:**

Eighteen volunteers developed infection by qPCR criteria of sustained viral load, one without symptoms and the remainder with mild illness. Infected volunteers showed statistically lower baseline-corrected global composite cognitive scores than uninfected volunteers, both acutely and during follow up (mean difference over all time points = −0.8631, 95% CI = −1.3613, −0.3766) with significant main effect of group in repeated measures ANOVA (F (1,34) = 7.58, p = 0.009). Sensitivity analysis replicated this cross-group difference after controlling for community upper respiratory tract infection, task-learning, remdesivir treatment, baseline reference and model structure. Memory and executive function tasks showed the largest between-group differences. No volunteers reported persistent subjective cognitive symptoms.

**Interpretation:**

These results support larger cross sectional findings indicating that mild Wildtype SARS-CoV-2 infection can be followed by small changes in cognition and memory that persist for at least a year. The mechanistic basis and clinical implications of these small changes remain unclear.

**Funding:**

This study was funded through the UK Vaccine Taskforce of the 10.13039/100011693Department for Business, Energy and Industrial Strategy (BEIS) of Her Majesty's Government. WT was funded by the 10.13039/501100000266EPSRC through the CDT for Neurotechnology Imperial College London.

**Research in context**

**Evidence before this study**

Searching PubMed for articles published prior to March 1 2024 that used the terms COVID-19 and cognition identifies a growing body of evidence associating COVID-19 with cognitive deficits that persists well beyond the acute illness. Most of these studies had small sample sizes and relied on self-reported cognitive symptoms. Fewer studies objectively measured cognition from task performance, and fewer still did so at large-population scale. As these studies were mainly cross-sectional, they are confounded by population factors known to correlate with cognitive performance. Critically, to date, no published studies have monitored cognitive change in response to prospectively controlled infection with SARS-CoV-2.

**Added value of this study**

This was the first, and likely will be the only, Human Challenge study in which unvaccinated virus naive volunteers were inoculated with Wildtype SARS-CoV-2. The 18 volunteers who showed sustained viral load post inoculation had measurable reductions in baseline-corrected cognitive scores relative to the 16 who did not show sustained viral load. In accordance with cross sectional population research, these differences were greatest for memory and executive functions; furthermore, they emerged after inoculation and remained evident at the one year study endpoint.

**Implications of all the available evidence**

These findings indicate that the previously reported cross sectional associations of cognitive and memory function with COVID-19 have a basis in persistent changes that occur after SARS-CoV-2 infection. The clinical implications of these changes remain unclear.

## Introduction

Converging evidence indicates that COVID-19 (symptomatic SARS-CoV-2 infection) may have a lasting impact on cognitive functions, both in severe and milder cases.^1^ Patients have reported “brain fog”, “poor memory”, and “difficulty finding words” months post recovery from acute COVID-19. Cross-sectional^2^, ^3^, ^4^, ^5^, ^6^, ^7^, ^8^, ^9^, ^10^, ^11^ and longitudinal^12^^,^^13^ studies have observed cognitive performance deficits a year or more after acute infection. Imaging has shown accelerated pre minus post infection brain atrophy including in memory and cognition-associated structures.^14^ Serum analyses have detected elevated markers of brain injury^15^^,^^16^ and blood biomarkers that predict cognitive deficits^17^ in hospitalised COVID-19 patients.

Much about the association between SARS-CoV-2 and cognition remains unknown due to reliance on cross-sectional and post-infection studies, where variables associated with increased SARS-CoV-2 exposure risk, e.g., pre-existing conditions, vocation, and sociodemographic factors, complicate the interpretation of correlations.^18^, ^19^, ^20^ Most relevantly, it remains to be confirmed under prospectively controlled conditions whether and how rapidly changes in cognition occur after SARS-CoV-2 infection, or how long it takes for any cognitive changes in milder cases to return to pre-infection levels. Furthermore, the exact cognitive processes that are most vulnerable to COVID-19 remain debated, although recent findings highlight a role for memory and executive functions.^2^^,^^3^^,^^11^

We analysed objective cognitive performance data from a computerised battery administered at two timepoints before inoculation, and then repeatedly up to one year after inoculation, during the SARS-CoV-2 Human Challenge Characterisation Study.^21^ We hypothesised that volunteers who had sustained viral load after inoculation (infected group) would show significantly worse pre inoculation baseline corrected cognitive performance relative to those who did not have sustained viral load (uninfected group) during acute illness, but that this difference would no longer be evident at the one year follow-up. Based on our recent cross sectional research with the same cognitive tasks, we further hypothesised that memory and executive functions would show the greatest sensitivity to SARS-CoV-2.

## Methods

### Ethics and protocol statement

This study was conducted in accordance with the protocol; the consensus ethical principles derived from international guidelines, including the Declaration of Helsinki and Council for International Organizations of Medical Sciences International Ethical Guidelines; applicable ICH Good Clinical Practice guidelines; and applicable laws and regulations. The screening protocol and main study were approved and given a favourable opinion by the UK Health Research Authority's Ad Hoc Specialist Ethics Committee (reference 20/UK/2001 (screening protocol dated 2 December 2020) and reference 20/UK/0002 (main protocol dated 16 February 2021)).

Written informed consent was obtained from all volunteers before screening and study enrollment. Volunteers were given up to £4565 to compensate for the time and inconvenience of taking part in the study (including at least a 17-day quarantine (3 days pre inoculation and minimum of 14 days post inoculation). This was calculated using the National Institute for Health Research (NIHR) formula and the UK national living wage. The study was overseen by a medical oversight committee (trial steering committee) with advice from an independent data and safety monitoring board, which assessed the study data.

Consultations were held with the Medicines and Healthcare products Regulatory Agency (MHRA) to clarify if the study qualified as a clinical trial. It was concluded that no medicinal product was under investigation, and therefore, the study was not classified as a clinical trial. Consequently, a EudraCT number was not required, and the study was registered on ClinicalTrials.gov with the identifier NCT04865237. These discussions and the registration process contributed to a delay in the study's start, resulting in the publication going live after the first volunteers had already been enrolled. The protocol was established on 08 February 2021, with no material changes before the study began on 06 March 2021. Detailed protocol history is available in the Supplementary Materials.

### Study population and design

Detailed description of the SARS-CoV-2 Human Challenge Characterisation Study and protocol https://clinicaltrials.gov/ct2/show/NCT04865237 have been published elsewhere.^21^ In brief, 36 healthy adults aged 18–30 years with no history of previous SARS-CoV-2 infection or vaccination were enrolled according to protocol-defined inclusion and exclusion criteria (see the Clinical Protocol in Supplementary Information and Appendix Table S1). Screening included assessments for known risk factors for severe COVID-19, including comorbidities; low or high body mass index (BMI); abnormal blood tests, including full blood count, renal and liver function, clotting and peripheral blood viral serology; spirometry; echocardiography; and chest radiography (Clinical Protocol in Supplementary Information).

Volunteers initiated the study between 6 March and 8 July 2021, with the final follow-up day on 11 July 2022. After screening and baseline assessment on two days, volunteers were inoculated intranasally with 10 TCID_50_ (median tissue culture infectious dose) of Wildtype SARS-CoV-2 (100 μl per naris). Subsequently, they were quarantined for a minimum of 14 days. They returned home after this time once they had two consecutive daily nose and throat swabs yielding no viral detection. Volunteers underwent follow-up assessments approximately 30, 90, 180, 270, and 360 days post-inoculation (Appendix Table S2). Six of the initial volunteers were assigned to receive pre-emptive remdesivir (100 mg intravenously for 5 days) to mitigate risk of severe illness.^21^ Twice-daily measurements of viral load were calculated during the quarantine period by qPCR of nasal and throat swabs (Appendix Section S1). Temperature was taken a minimum of four times per day. Thrice daily subjective symptom surveys were conducted, where volunteers rated nineteen symptoms on a four-point scale.^21^ For analysis, volunteers were categorised according to whether they met the criteria for laboratory confirmed infection as predefined in the study protocol, this being two quantifiable greater than lower limit of quantification RT-PCR measurements from mid turbinate and/or throat samples, reported on two or more consecutive timepoints, starting from 24 h post-inoculation and up to discharge from quarantine. Adverse events were recorded as part of a structured medical interview at each follow-up visit, using open questions to discuss any symptoms noted by volunteers since last contact with the study team, followed by further questioning to detail the nature and history of the complaint, and targeted physical examination if deemed necessary by the study physician.

### Cognitive assessment

Volunteers completed 11 computerised cognitive tasks (Appendix Section S2) from the Cognitron platform in fixed order on an iPad during two consecutive days pre-inoculation (baseline), each quarantine day, and each follow-up visit. Cognitron tasks are short and engaging while enabling global and domain-level analysis.^22^^,^^23^ They use automated algorithms to generate novel difficulty-balanced sequences of problems on-the-fly, which are designed to minimise learning of specific answers across timepoints while ensuring similar levels of task difficulty across volunteers and timepoints. The tasks were selected to give broad coverage of cognitive domains, some of which, preliminary cross-sectional research indicates were sensitive to SARS-CoV-2 infection.^2^ The main cognitive endpoint was a baseline-corrected global cognitive composite score (bcGCCS), defined as the baseline-corrected, standardised mean across all 11 tasks (Appendix Section S3). Exploratory cognitive endpoints were the baseline-corrected scores for individual tasks.

The 11 tasks included were, in order of administration (for full descriptions please see Appendix Section S2).

1. Motor Control–Measures visuomotor accuracy and reaction time
2. Object Memory (Immediate)–Measures short term precision recognition memory
3. Simple Reaction Time–Measures reaction time
4. Choice Reaction Time–Measures complex reaction time
5. 2D Manipulations–Measures mental manipulation of 2D visuospatial information
6. Four Towers–Measures mental manipulation of 3D visuospatial information
7. Spatial Span–Measures spatial working memory capacity
8. Target Detection–Measures attention and distractibility
9. Tower of London–Measures spatial planning
10. Verbal Analogies–Measures semantic reasoning
11. Object Memory (Delayed)–Measures medium term precision recognition memory

### Exploratory endpoints

Extending the original protocol due to emerging findings,^15^^,^^16^ blood samples from days −1, 0, 3, 7 and 14 from inoculation day were analysed for four serum markers of brain injury: neurofilament light (NfL), glial fibrillary acidic protein (GFAP), total tau, and Ubiquitin carboxy-terminal hydrolase L1 (UCH-L1) (Appendix Figure S1 and Section S4).

### Statistical analysis

As the cognitive arm of the wider SARS-CoV-2 human challenge characterisation study was not the primary objective of the study, no formal sample size calculations were performed. However, as per the protocol, a sample size of 30–90 volunteers was deemed sufficient to meet the primary objective of establishing a safe inoculation dose for challenge.

Data for the main and exploratory cognitive measures were split into six study phases for analysis: the quarantine phase and five follow-up timepoints. Single values for the main and exploratory outcome measures were calculated for the quarantine phase by taking the mean over the 14 post-inoculation days. Missing and non-compliant datapoints were imputed via linear interpolation (Appendix Section S5 and Figure S2). Imputation was used as opposed to casewise data removal as rates of missing data were expected to be low to avoid adding selection bias to the results and to conserve sample size.

Volunteers were divided into two groups for analysis: those who exhibited sustained viral infection, defined as having at least two consecutive quantifiable viral detections by qPCR (infected), and the remainder (uninfected). We applied a Shapiro–Wilk test over the residuals from the repeated measures models before repeated measures analysis of variance (RM-ANOVA) were performed to assess normality. RM-ANOVA was used to test whether there were differences in the main cognitive outcome measure over the six phases, with post-hoc t-tests conducted at each phase.

We ran sensitivity analyses to evaluate the robustness of the results for the main cognitive outcome when accounting for: baseline day, interpolation method, the two excluded volunteers (two participants were excluded from the analysis owing to seroconversion between screening and inoculation, identified after data collection), and learning effects. Tasks that were sensitive to learning effects were identified by performing a separate RM-ANOVA for each task, including quarantine time points for the uninfected volunteers. If the task showed a significant effect of time then it was deemed sensitive to learning. Two composite scores were then calculated using the tasks with no learning effect and tasks with a learning effect respectively, and the main analyses were repeated for these composites.

Further sensitivity analyses included covariates in the model to examine the impact on the results of sex, community acquired upper respiratory tract infection (URTI) (a time varying covariate) and remdesivir treatment. A mixed effects model was used to investigate this using timepoint, remdesivir treatment, infection status, sex and community infection as fixed effects and subject as a random effect to predict bcGCCS. This model was repeated for all combinations of baseline day, including or excluding seroconverted volunteers and learning composites.

We used t-tests to examine mean difference between groups for individual tasks to gauge their sensitivities to infection status as defined by the difference between the infected and uninfected groups’ change from baseline, meaned across the quarantine period and follow-up timepoints and divided by the standard deviation of a large pre-existing normative cohort dataset (Appendix Section S6).

Retrospective power analysis was conducted on the main effect of group for the main outcome RM-ANOVA. Details on these calculations and the resulting power curve plot can be seen in the supplement (Supplementary Methods Section S8 and Figure S6).

### Role of funding

This project was funded by the UK Vaccine Taskforce of the Department for Business, Energy and Industrial Strategy (BEIS) of Her Majesty's Government. WT was funded by the EPSRC through the CDT for Neurotechnology Imperial College London. The funders had no role in the conceptualization, design, data collection, analysis, decision to publish or preparation of the manuscript.

Authors were not precluded from accessing data in the study, and they accept responsibility to submit for publication.

## Results

Exact volunteer numbers and reasons for exclusion, viral load and biomarker results have been reported elsewhere.^21^ Of 36 inoculated volunteers, two were retrospectively excluded due to seroconversion to SARS-CoV-2 after screening and before inoculation. 18 were classified as infected (22.3 ± 2.8 years (18–27), six female, and 17 white, BMI 22.5 ± 1.9 (19.1–26.3)) and 16 as uninfected (21.1 ± 1.9 years (range 18–29), two female, 14 white, BMI 24 ± 2.7 (20–29)).^21^ The groups were not statistically different on any measured demographic variable (Table S1). Every volunteer attended each of their 5 follow up visits and the average time from inoculation of their final follow up was 362.5 days (Appendix Table S2). 15 volunteers acquired URTIs in the community after quarantine and before day 360 (Appendix Figure S3). As has been reported in detail elsewhere, volunteers experienced no to moderate symptom load, none were hospitalised or required supplemental oxygen.^21^

### Baseline-corrected composite cognitive score distinguishes infected and uninfected individuals following viral challenge

**Fig. 1:**
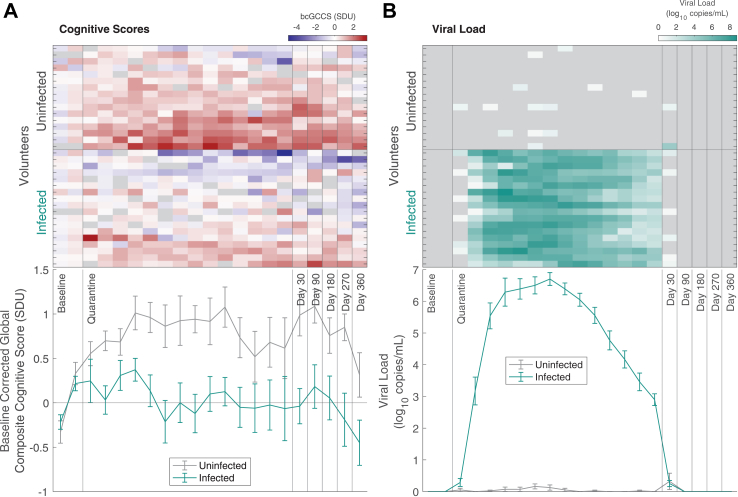
**Cognition and viral load of volunteers over study period**. Cognitive Scores and Viral load. A) baseline-corrected cognitive scores and B) viral load during the baseline, quarantine phase and follow-up timepoints. Upper plots show individual volunteer data, ordered within-group by mean cognitive score across all phases. Lower plots show mean data for infected (green) and uninfected (grey) groups with error bars representing the standard error of the mean.

Fig. 1A shows bcGCCS timecourses for the two groups over the whole study period. Distributions of residuals for bcGCCS scores deviated significantly from normality (sw-stat = 0.9804, p = 0.007), with a slight right skew (Appendix Figure S4). This was deemed to be acceptable given the robustness of ANOVA to moderate skew. When one-way RM-ANOVA was conducted with infected vs. uninfected as the between-subject factor and time (6 phases) as the within-subject factor, there was a significant main effect of group (η2 = 0.25, F (1,32) = 10.687, p = 0.003) and time (η2 = 0.132, F (5,160) = 4.926, p < 0.001) with no significant interaction (η2 = 0.013, F (5,160) = 0.491, p = 0.783).

Post hoc t-tests confirmed that the infected group bcGCCS did not differ from the uninfected group at baseline. The infected group also had statistically significantly lower bcGCCS than the uninfected group at each post-inoculation phase of the study with similar magnitude of difference throughout (Table 1). The study was not sufficiently powered to investigate a relationship between viral load, symptom severity or levels of brain injury markers on cognition; to guide future studies, correlation coefficients and confidence intervals for these relationships at each phase have been provided in the supplement (Appendix Tables S8, S9 and S10).

**Table 1:** Post hoc T-Tests of bcGCCS each phase of the study.

Phase	Estimate	95% CI	SE	tStat	p value
Baseline	0.218	−0.333, 0.769	0.271	0.805	0.427
Quarantine	−0.743	−1.232, −0.253	0.24	−3.091	0.004
Day 30	−1.025	−1.664, −0.386	0.314	−3.267	0.003
Day 90	−0.904	−1.566, −0.242	0.325	−2.784	0.009
Day 180	−0.703	−1.379, −0.026	0.332	−2.115	0.042
Day 270	−1.041	−1.77, −0.312	0.358	−2.908	0.007
Day 360	−0.764	−1.516, −0.011	0.369	−2.067	0.047

Posthoc analysis with unpaired two sample t-tests confirmed significantly lower baseline-corrected cognitive scores for the infected group at all post-inoculation stages at the two-tailed uncorrected threshold.

### Decrements in memory precision and executive function are main contributors to reduced task scores following infection

**Fig. 2:**
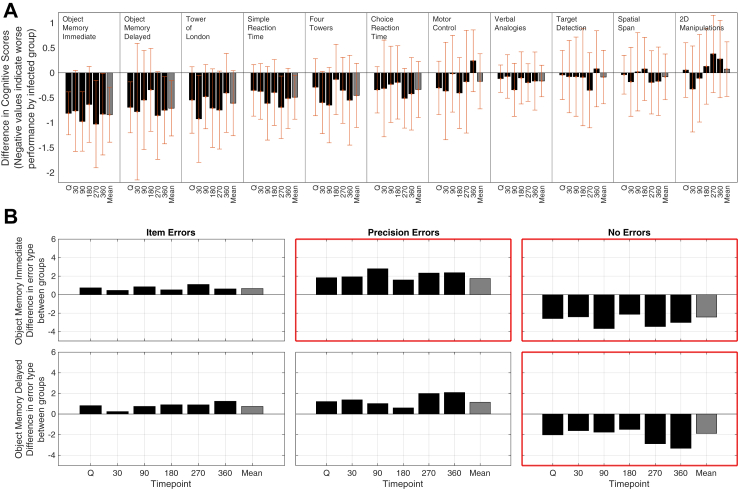
**Groups differences in individual task performance**. Group differences in cognitive task scores for each individual task at each timepoint. A) Each bar represents the mean score across the infected group minus the mean score for the uninfected group across each task and then across each timepoint, standardised by a large normative population dataset. The final bar (in grey) within each task represents the mean of the means across timepoints. Tasks are ordered by the magnitude of the mean difference across timepoints. Error bars indicate the 95% confidence intervals for the mean differences. B) The top row refers to the immediate object memory task and the bottom row, the delayed object memory task, each column refers to a different score taken from the task: Item Errors = The number of trials in which a volunteer got the entire item wrong, Precision Errors = The number of trials a volunteer got the item right but orientation or pose wrong and No Errors = The number of trials a volunteer got the image exactly correct. Each bar represents the difference between the groups at each phase of the trial. Bars above the 0 line indicate that the infected group did more of this type of outcome. Boxes with red borders indicate if there was a significant main effect of group in a one way RM-ANOVA for that task or score.

**Fig. 3:**
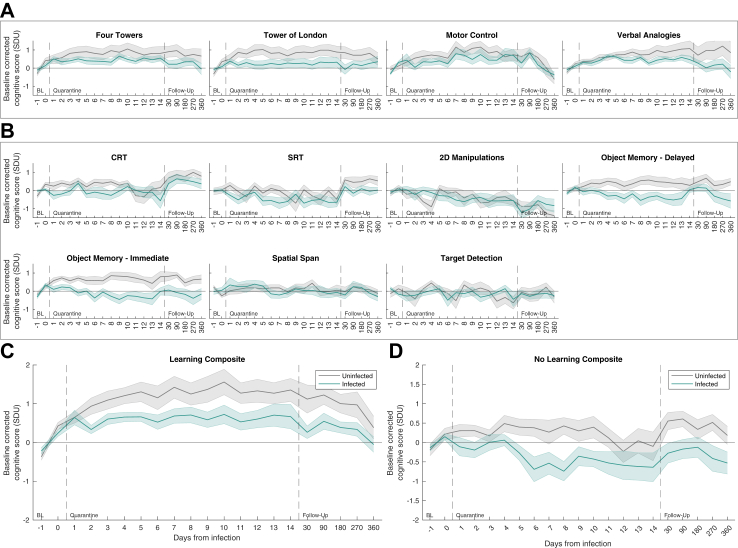
**Learning effects in individual tasks and subsequent composites**. Timecourses for individual tasks and composites formed from tasks with vs. without learning effects. A) Timecourses for tasks that had learning effect in the uninfected group over the quarantine period. B) Timecourses for tasks that had no learning effects in the uninfected group over the quarantine period. C) Composite score timecourse including only tasks that had an effect of time. Note improving performance with time during frequent testing followed by reducing performance with forgetting across the sparser follow-up visits. D) Composite score timecourse including only tasks that did not have an effect of time, note lack of learning and forgetting effects. Green lines are the mean of the infected group and grey lines are the mean of the uninfected group. Shaded regions are the standard error of the mean.

Individual tasks with significant main effect of group included Object Memory Immediate (η2 = 0.233, F (1,32) = 9.717, p = 0.004) and Delayed (η2 = 0.138, F (1,32) = 5.138, p = 0.03) and Simple Reaction Time (η2 = 0.136, F (1,32) = 5.033, p = 0.032). The largest effect sizes were for the Object Memory Task at Immediate and Delayed recognition, followed by Tower of London, which are designed to measure memory and executive planning functions, respectively (Fig. 2A and Appendix Table S3). Object Memory also distinguishes between errors due to the forgetting of items and poor precision in item feature encoding. Residuals for Object Memory error rates also were slightly skewed from normality, however as with the bcGCCS, the distributions were considered suitable for RM-ANOVA (Appendix Figure S5). Analysis of error rates showed that the infected group had significantly reduced memory precision at the immediate (η2 = 0.197, F (1,32) = 7.866, p = 0.008) but not delayed (η2 = 0.081, F (1,32) = 2.812, p = 0.103) timescales (Fig. 3B).

### Group differences in baseline-corrected cognitive scores after inoculation are evident for tasks that do not have significant cross-session learning effects

Some cognitive tasks are characterised by learning and forgetting effects across sessions (Fig. 3), which can complicate the interpretation of longitudinal results. Therefore, two further composites were calculated, grouping the tasks according to whether or not they showed significant learning effects across the post inoculation quarantine timepoints (Fig. 3C and D). Repeating the RM-ANOVA on these composites confirmed the main effect of group in the no-learning composite (η2 = 0.177, F (1,32) = 6.895, p = 0.013), with no main effect of time (η2 = 0.042, F (5,160) = 1.434, p = 0.215) or interaction (η2 = 0.013, F (5,160) = 0.446, p = 0.816). There was also a significant main effect of group (η2 = 0.126, F (1,31) = 4.474, p = 0.043) and time (η2 = 0.201, F (5,155) = 7.88, p < 0.001) and non-significant interaction (η2 = 0.009, F (5,155) = 0.365, p = 0.872) for the learning composite.

### Robustness of results to community infection, baseline day, interpolation method, sex, remdesivir and exclusion criteria

Community URTI was more common in the uninfected group, which could reduce the observed cross-group effects. Furthermore, it is possible that remdesivir modulated cognitive change. Therefore, the robustness of the model was evaluated using linear mixed effects modelling. While there was a significant main effect of the challenge infection in bcGCCS (Estimate = −0.920SDC, 95% CI = −1.492, −0.347, p = 0.002) and in the no-learning composite (Estimate = −0.949SDC, 95% CI = −1.562, −0.335, p = 0.003), there was no significant main effect of community infection, sex or remdesivir (Appendix Section S7 and Table S4).

Cognitive performance can fluctuate across days; therefore, robustness of the cross-group difference was evaluated when using Day −1 or Day 0 as the baseline, rather than the mean of the two. The main effect of group was materially unchanged when using either day for baseline correction with the bcGCCS (Day −1: η2 = 0.206, F (1,32) = 8.279, p = 0.007, Day 0: η2 = 0.187, F (1,32) = 7.352, p = 0.011) and no learning composite (Day −1: η2 = 0.131, F (1,32) = 4.832, p = 0.035, Day 0: η2 = 0.142, F (1,32) = 5.286, p = 0.028). It remained below the alpha threshold for baseline day −1 in the learning composite (Day −1: η2 = 0.137, F (1,31) = 4.91, p = 0.034, Day 0: η2 = 0.082, F (1,31) = 2.777, p = 0.106) (Appendix Tables S5 and S6).

The method by which we interpolated the missing and non-compliant data points could affect the results, so the main RM-ANOVA was repeated using class mean, cubic spline and K-nearest neighbour (knn) interpolation methods, rather than linear. The main effect of group was conserved (class mean: F (1,32) = 10.983, p = 0.002, cubic spline: F (1,32) = 11.181, p = 0.002, knn: F (1,32) = 11.106, p = 0.002), the main effect of time was conserved (class mean: F (5,160) = 5.024, p < 0.001, cubic spline: F (5,160) = 4.594, p = 0.001, knn: F (5,160) = 4.99, p < 0.001) and there was still no significant group by time interaction (class mean: F (5,160) = 0.774, p = 0.569, cubic spline: F (5,160) = 0.415, p = 0.838, knn: F (5,160) = 0.669, p = 0.648).

Finally, rerunning the analyses of bcGCCS including the two volunteers who had been infected in the community between screening and baseline but not during the trial did not materially change the results: main effect of group (η2 = 0.191, F (1,34) = 8.006, p = 0.008), main effect of time (η2 = 0.128, F (5,170) = 5.068, p < 0.001) time-by-group interaction (η2 = 0.011, F (5,170) = 0.418, p = 0.836). RM-ANOVA and cross group differences are in Appendix Tables S5 and S6 for all sensitivity analyses.

### Exploratory analysis of serum markers of brain injury

RM-ANOVA of GFAP showed a group ∗ time interaction (η2 = 0.083, F (3,93) = 2.995, p = 0.035), with increased levels at 14 days after inoculation relative to individual pre-inoculation baselines in the infected vs. the uninfected group. The group ∗ time interactions for NfL and Tau were statistically non-significant (Appendix Figure S1 and Table S7). UCH-L1 could not be evaluated as assays had high coefficients of variance between the replicates with only a small proportion reaching the lowest level of quantification.

## Discussion

Human infection challenge studies offer unique insights into diseases by precisely controlling viral exposure timing and dose, characterising pre-inoculation baseline measures, and monitoring volunteers post-inoculation.^24^ Monitoring is then conducted under carefully controlled conditions, which mitigates environmental factors that may confound such measures. These strengths enable the detailed analysis of subtle post-infection changes, providing controlled insights into disease mechanisms with modestly sized cohorts. Using state-of-the-art computerised cognitive assessment technology (Cognitron) we produced sensitive multi-dimensional profiles of cognitive change across repeated timepoints spanning from just before to one year after infection with Wildtype SARS-CoV-2. We found that volunteers who exhibited sustained viral load after inoculation with SARS-CoV-2 performed worse on a measure of global cognition, than volunteers who did not exhibit sustained viral load. This deficit persisted up to a year after inoculation.

The Cognitron assessment technology had already shown cross-sectional associations with lower cognitive scores in people after COVID-19 vs. uninfected populations, which scaled with virus variant, acute illness severity and symptom duration.^2^^,^^3^^,^^6^^,^^11^ However, due to the lack of detailed timecourse data and pre-infection baselines it was unclear whether these differences emerged after SARS-CoV-2 infection, and if so, how quickly they developed and recovered. The results presented here confirm that prospectively controlled infection with SARS-CoV-2 is followed by objectively measurable reductions in cognitive performance.^25^

Past studies have provided mixed evidence regarding the recovery of cognitive functions after COVID-19, with some studies supporting a positive relationship between cognition and time since illness,^6^^,^^9^^,^^12^ whereas others have reported persistent cognitive deficits.^2^^,^^13^^,^^14^^,^^26^ This contradictory evidence likely reflects confounding factors e.g., virus variant, acute illness severity, use of measurement instruments with different sensitivities, and a lack of pre-infection baseline. Here, the lack of a group-by-time interaction in the bcGCCS timecourses combined with the similar magnitude of cross-group differences up to the one year follow up timepoint indicate that for mild infected cases, any normalisation of the observed cognitive changes is at best gradual.

When the average effect size across cognitive tasks was scaled by the standard deviations within our large pre-existing normative dataset (Appendix Section S6), they were −0.37 standard deviations over the quarantine period, and across the follow-up timepoints were −0.42 standard deviations. This is comparable to the effect sizes observed in our previously reported cross-sectional citizen science research,^2^ which had been collecting large-scale cognitive performance data from a large population sample during the first pandemic wave in the UK. This effect size is also comparable to the deficits observed for recovered cases up to three years after recovery from short duration Wildtype and Alpha variant infections in our recently published epidemiological analysis of data from 112,964 adults from the REal-time Assessment of Community Transmission (REACT) study in England.^11^ Together, these studies converge in supporting the hypothesis that COVID-19 may cause a persistent objectively measurable change in cognition both in volunteers under experimental conditions and in the general population.

Here, the most robust cross-group differences were observed in the Immediate and Delayed Object Memory Task (Fig. 2). Notably, the same task was also the most sensitive to COVID-19 in our large-scale population analysis of cognitive deficits in the REACT cohort, comprising >112,000 UK residents.^11^ More broadly, this result accords with previous studies that report memory to be amongst the most sensitive domains to COVID-19.^2^^,^^6^^,^^11^ In both studies, memory performance showed similar effect sizes at immediate and delayed timescales. These results indicate that post-COVID-19 memory deficits may relate to encoding rather than consolidation or fatigue with longer assessment time. Speculatively, the fact that memory precision was reduced is of interest as this process has been closely associated with medial temporal lobe functions.^27^ Taken together with UKBioBank findings of accelerated atrophy in these brain regions after COVID-19,^14^ future research might investigate the impact of COVID-19 on the component processes of memory function, their relationship to medial temporal lobe circuits, and the interaction between attention and memory systems.

Notably, none of the volunteers reported subjective cognitive deficits. This apparent discrepancy between objective and subjective measures could be interpreted as indicating that the tasks are sensitive enough to detect small changes in cognition that are too subtle for the volunteer to be metacognitively aware of. It is important to note though that this interpretation is limited by the lack of specific questions about lasting changes in cognition–instead, volunteers underwent a structured interview about their overall health. Within the broader literature, larger observational studies have reported mixed results. Subjective symptoms have been reported 36 months post infection,^28^ which accords with the observation of objectively measured deficits in task performance a year or more post infection with Wildtype virus in our epidemiological research,^11^ and with the objective measures observed up to the 12 month final timepoint in the present study. However, correlations between objective measures of cognition and subjective assessment of cognition after COVID-19 are modest,^29^ and hard to confirm, particularly in less severe cases.^30^ Given that no direct assessment of subjective cognitive symptoms was performed and the sample size was small, the complex interaction between objective cognitive ability and subjective cognition cannot be elucidated in the current study. Relatedly, the clinical relevance of the observed cognitive changes remains unclear.

A key consideration is that cognitive task measures tend to be sensitive to novelty, learning and forgetting. The tasks applied here were specifically designed to minimise such effects, but for some tasks they are still evident when they are performed on a daily basis, which must be accounted for in the analysis. Novelty effects for the tasks occur primarily between the first and second time that volunteers perform them, which was prior to inoculation and subtracted when contrasting the infected and uninfected groups. Furthermore, although some tasks exhibited a learning effect post inoculation, the main effect of group was conserved when analysing just those tasks that did not show such learning effects. Taken together, this means that it is unlikely that the differences in baseline-corrected cognitive scores between the infected and uninfected groups have a basis in differential learning rates.

Our study has limitations. Although it was conducted under controlled conditions, the small sample size was not intended to enable confirmation of associations between cognitive changes and specific biomarkers or patient-reported symptoms. We provide correlation coefficients between levels of serum markers of brain injury and cognitive change to guide power calculations when designing future studies (Appendix Table S7). A further limitation is that the study population were mostly white males and as such we should be cautious in extrapolating these results to all members of the general population. Additionally, the study was of Wildtype SARS-CoV-2; therefore, we should be cautious generalising the results to more recent variants, which studies have shown may have attenuated cognitive implications.^11^ Similarly, we have previously reported more substantial cognitive deficits in hospitalised COVID-19 cases^2^, ^3^, ^4^, ^5^ and for people with persistent symptoms consistent with Long COVID. Additional psychological or neurological mechanisms may contribute to cognitive deficits in these populations and accordingly, their fine-grained profile of deficits across tasks differs.

There was some missing data, though this is unlikely to have been the basis of the observed cross group differences as the majority of data were present (3.6% of tasks missing or unusable in the infected group and 4.5% of tasks missing or unusable in the infected group).

Finally, uncontrolled community infection after the quarantine period complicates the results at later timepoints. However, the community-infection rate was higher in the group for whom infection was unsuccessful; therefore, we would expect the cross-group differences to be attenuated. Furthermore, the results remained statistically significant when sensitivity analysis included community infection as a nuisance variable.

In conclusion, this study confirmed that prospectively controlled infection with Wildtype SARS-CoV-2 is followed by objectively measurable reductions in cognitive task performance that can persist for at least a year. Immediate and delayed memory, and executive function were the most sensitive cognitive domains. Future research should examine the biological mechanisms that mediate this relationship, determine how they differ to those observed for other respiratory infections,^31^ and explore whether targeted interventions can normalise these memory and executive processes.

## Contributors

William Trender∗–data curation, formal analysis, methodology, software, writing—original draft.

Peter J Hellyer–software.

Ben Killingley–conceptualisation, methodology, investigation.

Mariya Kalinova–conceptualisation, methodology, investigation.

Alex J. Mann–conceptualisation, methodology, investigation, writing—review & editing.

Andrew P. Catchpole–conceptualisation, methodology, investigation.

David Menon–conceptualisation, methodology, investigation.

Edward Needham–conceptualisation, methodology, investigation.

Ryan Thwaites–methodology, data curation.

Christopher Chiu–conceptualisation, methodology, investigation, funding acquisition, writing—review & editing.

Gregory Scott∗–methodology, writing—review & editing, supervision.

Adam Hampshire∗–conceptualisation, methodology, software, writing—review & editing, supervision.

∗ These authors had access to and verified the data.

## Data sharing statement

Data can be shared with investigators whose proposed use of the data has been approved by the UK Vaccine Taskforce Human Challenge Steering Committee to achieve the aims in the approved proposal. Proposals should be directed to c.chiu@imperial.ac.uk. To gain access, data requestors will need to complete a data request form and sign a data access agreement.

## Declaration of interests

AH is founder and director of Future Cognition Ltd and H2 Cognitive Designs, which develop custom cognitive assessment tasks and provide online assessment services respectively, primarily within the academic research sector.

CC had funding to institution from the UK Vaccine Task Force and Wellcome Trust.

DM had support for the present manuscript in the form of payments to the institution from the UKRI (UKRI, The COVID-19: Clinical Neuroscience Study (COVID-CNS) (Co-I) and UKRI. Convalescent plasma for COVID-19 patients (Co-I)), the Addenbrooke's Charitable Trust (COVID-19: understanding its long-term respiratory, cardiac and neuro-psychiatric sequelae and the determinants of adverse outcomes), the NIHR (NIHR Cambridge Biomedical Research Centre). They also received support unrelated to the current manuscript in the form of consultancy fees and research support from: Neurotrauma Sciences, Lantmannen AB, GlaxoSmithKline Ltd and PressuraNeuro Ltd.

EN received a portion of their salary from Brain Research UK during the periods in question.

PJH is Co-founder and director of H2 Cognitive Designs LTD, which markets online cognitive testing platforms for healthcare and research. In this role he receives Remuneration.

RT received support for the present manuscript from UK Vaccine Taskforce of the Department of Business, Energy and Industrial Strategy of Her Majesty's Government (BEIS) and the Wellcome Trust (grant no. 224530/Z/21/Z). Also payment or honoraria for lectures, presentations, speakers bureaus, manuscript writing or educational events from AstraZeneca.

WT is employed by H2 Cognitive Designs LTD, which markets online cognitive testing platforms for healthcare and research.

AJM is a holder of shares in hVIVO Ltd and an employee of hVivo Ltd.

MK, GS, BK and APC have no declarations of interest.

## Appendix A Supplementary data

Protocol

Statistical analysis plan
