# Supplementary material for: Changes in memory and cognition during the SARS-CoV-2 human challenge study
Source: eClinicalMedicine. 2024 Sep 21;76:102842. doi: 10.1016/j.eclinm.2024.102842 (PMC11447363; doi:10.1016/j.eclinm.2024.102842)
Supplement: Protocol [file mmc1.pdf]

CLINICAL STUDY PROTOCOL

A dose finding human experimental infection study in healthy subjects using a GMP-produced SARS-COV-2 wild type strain (SARS-CoV-2 Characterisation Study)

**Version and Date of Protocol:**	**Final V 7.0 _09Nov2021**
**Sponsor Protocol Number:**	**20IC6437**
**hVIVO Protocol Number:**	**HVO-vCS-003**
**Sponsor:**	**Imperial College London**<br>Room 221, Level 2, Medical School Building<br>Norfolk Place, London W2 1PG
**Funder**	**UK Vaccine Taskforce, Department of Business, Energy and Industrial Strategy.**
**IRAS ID:**	**292098**
**REC Reference:**	**20/UK/0002**

Confidentiality and Protections Statement:

This document contains confidential information and must not be disclosed to anyone other than the study staff and members of the Independent Ethics Committee/Institutional Review Board or Competent Authorities. The information in this document cannot be used for any purpose other than the conduct or evaluation of the clinical investigation without the prior written consent of hVIVO and Imperial College London.

Personal data included in the protocol is subject to General Data Protection Regulation (GDPR: EU 2016/679) considerations and protections.

Sponsor Statement:

This protocol was subjected to critical review. The information it contains is consistent with current knowledge of the risks and benefits of the study intervention, and with the moral, ethical and scientific principles governing clinical research as set out in the current Declaration of Helsinki and the principles of International Council for Harmonisation (ICH) Good Clinical Practice (GCP).

Sponsor Signatory:

[redacted]

Investigator Agreement:

I have read the protocol and agree to conduct the study in accordance with the approved protocol and any future amendments, the Declaration of Helsinki, the principles of ICH GCP, the current regulatory requirements as detailed in the Medicines for Human Use (Clinical Trial) Regulations (Statutory Instrument 2004/1031) and all subsequent amendments, the UK Data Protection Act 2018, any other applicable laws and guidance.

I agree to conduct the procedures described in this protocol according to these guidelines and to appropriately direct and assist the staff under my control.

Chief Investigator (CI) Signatory:

[redacted]

Investigator Agreement:

I have read the protocol, and agree to conduct the study in accordance with the approved protocol and any future amendments, the Declaration of Helsinki, the principles of ICH GCP, the current regulatory requirements as detailed in the Medicines for Human Use (Clinical Trial) Regulations (Statutory Instrument 2004/1031) and all subsequent amendments, the UK Data Protection Act 2018, any other applicable laws and guidance.

I agree to conduct the procedures described in this protocol according to these guidelines and to appropriately direct and assist the staff under my control.

Principal Investigator (PI) QMB and Quarantine Site (including Follow Up Visits) Signatory:

[redacted]

Study Personnel Contact Information

[redacted]

[redacted]

[redacted]

Funder

This study is funded by the UK Vaccine Taskforce, Department of Business, Energy and Industrial Strategy.

This protocol describes the SARS-CoV-2 Characterisation Study and provides information about procedures for entering participants. The protocol should not be used as a guide for the treatment of other participants; every care was taken in its drafting, but corrections or amendments may be necessary. All amendments and changes will be discussed and agreed with all parties, relevant approvals obtained and implemented at the recruiting site.

Problems relating to this trial should be referred, in the first instance, to hVIVO Services Ltd., as a delegate of the sponsor.

The study will adhere to the principles outlined in the UK policy framework for health and social care (V3.3 07/11/17). This trial will adhere to the principles outlined in the Medicines for Human Use (Clinical Trials) Regulations 2004 (SI 2004/1031), amended regulations (SI 2006/1928) and the International Conference on Harmonisation Good Clinical Practice (ICH GCP) guidelines. It will be conducted in compliance with the protocol, the Data Protection Act and other regulatory requirements as appropriate.

Protocol History

Document	Date	Amendment Type
COVHIC001 Initial Clinical Trial Protocol (v3.0)	08Feb2021	Not applicable. First Version.
COVHIC001 Clinical Trial Protocol (v4.0)	24Feb2021	NSA1
COVHIC001 Clinical Trial Protocol (v5.0)	11Mar2021	SA2
COVHIC001 Clinical Trial Protocol (v6.0)	20May2021	SA3
COVHIC001 Clinical Trial Protocol (v7.0)	09Nov2021	SA4

Amendment: NSA 1 24Feb2021

Overall Rationale for the Amendment:

Page # and Section Name	Description of Change	Brief Rationale
Throughout	Incorrect section 0 link updated	Administrative correction
p24-32 SoA	Superscript w updated and added to GAD-7 and PHQ-9 questionnaire rows	Clarification
p24-32 SoA	Superscript v updated around cognitive testing timings	Clarification
p24-32 SoA	Superscript j updated	Clarification
p24-32 SoA	BD/TDS/QDS updated	Clarification
p24-32 SoA	Added arrows to clarify that SAE and con med recording will take place from screening through to follow up	Clarification
p24-32 SoA	Blood serum updated to blood plasma markers	Administrative correction
p24-32 SoA	Blood CPT - PBMCs & Plasma replaced with Blood Lithium Heparin – PBMCs, other blood cells, +/- plasma	Administrative correction
p24-32 SoA	Nasopharyngeal Transwab for cells for RNA - Nasopharyngeal replaced with mid-turbinate	Administrative correction
p24-32 SoA	DNA Paxgene removed	Administrative correction
p58, Risk determination	Nasosorption strip - 2 minutes corrected to 1 minute	Administrative correction
p57, Risk assessment	Incorrect title removed	Administrative correction
p71, 92, 118, figure 1	Deleted figure from caption	Administrative correction

Page # and Section Name	Description of Change	Brief Rationale
p73 Follow up phase	Added telephone calls every 2-3 days between discharge from Q and first follow up visit already in soa	Clarification
p75 Inclusion Criteria	Criterion 4 – Numbering corrected. 4 weeks corrected to 2 weeks for contraception and "first dose" corrected to "inoculation"	Administrative correction and clarification
p75 Inclusion Criteria	Criterion 4 – corrected to 2 weeks for contraception and "first dose" corrected to "inoculation"	Clarification
p75, 126,135	References to IMP – replaced with "study virus or Remdesivir." Or "Remdesivir" as applicable.	Administrative correction
p77 Exclusion Criteria	Criterion 10 – g corrected to kg	Administrative correction
p79 Exclusion Criteria	Criterion 18 – Asterix removed	Administrative correction
p96, criteria for escalation	Physician replaced with clinician	Clarification
P98, p153 ECG parameters	QTcB and QTcF added back in following confirmation that RFH machines can provide this readout	Administrative correction
p99, Radiology	Wording updated for clarity	Clarification
p100, Echocardiogram	removed in the quarantine unit at the participants' bedside	Administrative correction
p100 Spirometry	Wording around FEF25-75 included	This will be an exploratory measurement, not included in eligibility assessments.
p104	Incorrect reference to PK removed	Administrative correction
p107	Respiratory pathogen screen – nasopharyngeal updated to mid turbinate	Administrative correction
p108 Mask wearing sampling	Corrected to day -1 (previously – was omitted in error)	Administrative correction
p129, Table 12	HbA1c clarified as only required on admission	Clarification
p141	Broken table reference corrected	Administrative correction
p141, Table 17	PV reporting details included	Clarification

Amendment SA 2 11Mar2021

Overall Rationale for the Amendment:

Page # and Section Name	Description of Change	Brief Rationale
Throughout document	Minor typographical and formatting corrections	Correction of administrative error
p28, p33 and p 106	Update to frequency of UPSET smell tests, update to footnote u, update to text around UPSIT smell test	Clarification/Correction of administrative error
p33 SoA	Update to footnote o	Clarification/Correction of administrative error
p30 SoA	Mask wearing removed from day 0	Clarification/Correction of administrative error
p74, Follow up phase	Addition of day 270 (previously omitted in error)	Clarification/Correction of administrative error
p75, Inclusion criterion 4	Clarification about timing of contraception regarding Remdesivir and study virus.	Clarification/Correction of administrative error
p76, Inclusion criterion 5	Addition of “if applicable” regarding rescue medication	Clarification/Correction of administrative error
p79, Exclusion criterion 12	Corrected parentheses	Clarification/Correction of administrative error
p110 Discharge Criteria	Removal of reference to home visits	Clarification/Correction of administrative error
p110 Discharge Criteria	Update to wording to clarify home testing	Clarification/Correction of administrative error
p132 Appendix 3	Updates and amendments to table to reflect laboratory AE values	Clarification/Correction of administrative error
p140 Vital signs	Updates to Bradycardia grading	Clarification/Correction of administrative error

Amendment SA 3 20May2021

Overall Rationale for the Amendment:

**Page # and Section Name**	**Description of Change**	**Brief Rationale**
p.18 Protocol Synopsis	Change to anti-viral treatment wording - pre-emptive treatment with Remdesivir or Early Rescue with REGEN-COV or no pre-emptive or rescue treatment.	Change to wording following DSMB meeting 19Apr2021
p.19	Change to wording on Discharge Criterion. Subjects will remain in the quarantine unit until these criteria are met.<br><br>Two consecutive swabs with qPCR Ct >33.5 AND negative culture.<br><br>Lateral flow tests may be used in place of culture, if culture data is not available.<br><br>A qualitative PCR may be used in place of quantitative PCR, if the latter is not available.<br><br>At the PI's discretion, if protracted quarantine is deemed to be causing harm to the participant's mental or physical health and no viable virus is detected by culture or a lateral flow test is negative.<br><br>For non-infected subjects:<br><br>2 consecutive swabs with undetectable virus by PCR only prior to discharge	Change to wording following DSMB meeting 19Apr2021
p.19	Subjects will remain in the quarantine unit for a minimum of 14 Days from inoculation.	Clarification
p.21	Change to wording on Sample Size to include 'and treatment regimen' and 'up to a total of 90 subjects overall'	Clarification
p.23 SoA	Vital signs, Temperature, Diary Card, added (X) to D-2 assessment.	Clarification
p.24 SoA	HIV, Hepatitis A B & C added (X) to Day-2/-1	Clarification

Page # and Section Name	Description of Change	Brief Rationale
p.25 SoA	Added wording – Mid Turbinate or nasopharyngeal swab for cells for RNA (g)	Administrative change to wording
p.25 & p.26 SoA	Mask Wearing and Environmental Sampling added (X) at Extended stay	Clarification
p.25 & p.29 SoA	Added wording to clarify Chest X-Ray and ECHO will not be repeated if a subject is reinvited to a future Admission/Quarantine.	Clarification
p.27	Updated wording to reflect current National Vaccine Program roll-out – removal of 'vaccines in the near future'.	Administrative correction
p.27	Addition of wording to clarify Regeneron – 'Pre-clinical and clinical experience with REGEN-COV (Regeneron Monoclonal Antibody cocktail) for COVID-19. Background and reference to previous clinical studies.	Additional of wording for clarification.
p.29 SoA & p.109	Change to timing of Mask Wearing from 'up 60 minutes' to 'between 60 – 90 minutes'.	Additional wording for clarification and accuracy of assessment requirement.
p.51	Updated replacement of Gilead Remdesivir Fact Sheet.	Update.
p.67	Clarification on Screening activities in the Screening Protocol up to the time of signing the consent for the main study protocol (between D-90 to Day -2).	Clarification.
p.69	Additional wording to reference Royal Free Hospital elective admission procedure.	Clarification.
p.69	Quarantine Phase – adjustment to wording 'subjects will stay overnight for a period of at least 16 days'.	Clarification.
p.71 & p.72	Inclusion criterion, Item 4 & 5 – adjustment to wording on contraception for both male and female participants to add willing to use contraception 'until 6 months after receipt of final dose of study virus or intervention treatment'.	Clarification.
p.72	QCOVID removal of QCOVID tool as an Inclusion Criterion but remains in the study and ICF.	Change to Inclusion Criterion.
p.79-81	Section 4 – Study Intervention – addition of REGEN-COV information.	Clarification.
p.88 & p.89	Update to Dose Escalation, De-escalation and Confirmation Figure 4.	Clarification.

Page # and Section Name	Description of Change	Brief Rationale
p. 93	Criteria for Clinical Evaluation of Participant – re-wording of sentence to accurately reflect procedure and inclusion of the RFH Patient Assessment and Response Team (PARRT).	Clarification.
p.106 – p.109	Re-wording of SARS-CoV-2 Discharge Process.	Clarification.
p.108	Environmental and Mask Wearing sampling – re-wording.	Clarification.
p.111	Addition of wording - Reporting of Events related to REGEN-COV – addition of wording to reference SAEs / AEs and Grading.	Clarification.
p.21 & p.117	Sample Size – change to wording to include 'dose-level and treatment regimen' and 'up to 90 subjects overall'	Administrative correction.
p.123	Additional wording to cover reimbursement of extended stays in the quarantine unit, beyond the Day 14 post-inoculation quarantine period.	Administrative change.
p.129	Additional wording – HbA1c (only required at screening).	Clarification.
p.138	Rewording to add 'PI/Investigator will use his/her clinical judgement to assign severity grades above Grade 1, based on evaluation of clinical signs and symptoms'.	Administrative correction
	**Administrative Changes**	
Throughout	Section Numbering	Administrative correction for clarification
Throughout	Format, Tables, Figures, Document Alignment.	Administrative correction for clarification
Throughout	Re-wording for clarity that does not change the protocol.	Administrative correction for clarification
Throughout	Minor typographical errors.	Administrative correction
p.6	Updated RGIT website.	Administrative correction

Amendment SA 4 09Nov2021

Overall Rationale for the Amendment:

Page # and Section Name	Description of Change	Brief Rationale
Investigator Agreement (p. 4)	Change in Principal Investigator (PI) to remove Dr Ben Killingley and transfer responsibilities to Dr Mariya Kalinova	Change in PI
Cover page (p. 1) and protocol history (p. 8)	- Addition of summary of changes tables<br>- Removal of EudraCT number	Administrative corrections
Section 5.18 (p.107) and SoA (p.27)	'Mid-turbinate or nasopharyngeal' amended to 'Mid-turbinate and/or nasopharyngeal'	Update to reflect a non-substantial change implemented
Throughout	Minor administrative corrections	Administrative correction

List of figures

Figure 1 SARS-CoV-2 viral load in naturally infected patients by qPCR and culture. 37

Figure 2 Outcomes of 20,133 hospitalised COVID-19 patients by age 44

Figure 3 Frequency of individuals with the most common COVID-19 associated symptoms by duration. 48

Figure 4 Group and Dose Escalation Scheme 91

List of Tables

Table 1 Schedule of Activities 25

Table 2 Key notes for SoA 29

Table 3 Frequency of COVID-19 related symptoms in healthy 18-30 year olds with PCR-confirmed infection 46

Table 4 Duration of COVID-19 related symptoms in healthy 18-30 year olds with PCR-confirmed infection. 47

Table 5 Representative risk assessments using QCOVID 50

Table 6: Risk Assessment 59

Table 7 - Study Interventions 82

Table 8: Permitted medications and restrictions 89

Table 9 Prohibited Medications 90

Table 10 Study Stopping Rules 98

Table 11 List of symptoms recorded by subjects 105

Table 12 Protocol-Required Safety Laboratory Assessments 132

Table 13 Classification of Adverse Event Severity 141

Table 14 Severity grading criteria for physical observations 142

Table 15 Classification of Adverse Event Relationship 144

Table 16 Classification of Adverse Event Outcome 145

Table 17 Contact Details for Reporting SAEs 146

Table of Contents

Contents

CLINICAL STUDY PROTOCOL **1**
Investigator Agreement: ..... **Error! Bookmark not defined.**
Investigator Agreement: 4
Protocol History 8

Table of Contents **16**

1. Protocol Synopsis **20**
1.1 Protocol Summary 20
1.2 Schedules of Activities (SoA) 25

2. Background to Research **32**
2.1 Why is the COVID-19 challenge model so important? 32
2.2 Research Strategy 35
2.3 Virology of SARS-CoV-2 36
2.4 Virological Correlates of Infection 36
2.5 Immunology of COVID-19 37
2.6 Clinical outcomes of SARS-CoV-2 infection 39
2.7 Mitigating Risk in Experimental Human SARS-CoV-2 Infection 48
2.8 Pre-clinical and clinical experience with Remdesivir for COVID-19 51
2.9 Pre-clinical and clinical experience with REGEN-COV (Regeneron Monoclonal Antibody cocktail) for COVID-19 52
2.10 Human respiratory virus challenge studies in the UK 54
2.11 Study Rationale 54
2.12 Research Hypotheses 57
2.13 Benefit/Risk Assessment 57
2.13.1 Vulnerable persons 63
2.13.2 Benefit assessment 63
2.13.3 Overall benefit : risk conclusion 63
2.14 Objectives and Endpoints 64

3. Study Design **70**
3.1 Overall Design 70
3.2 Screening Phase 70
3.3 Study Specific Consent 71
3.4 Quarantine Phase 72
3.5 Follow Up Phase 73
3.6 End of Study Definition 73
3.7 Study Population 73
3.8 Inclusion Criteria 73
3.9 Exclusion Criteria 75
3.10 Lifestyle Considerations 81
3.10.1 Meals and dietary restrictions 81
3.10.2 Caffeine, alcohol and tobacco 81
3.10.3 Activity 81
3.11 Screen Failure 81

4. Study Intervention(s) **82**
4.1 Summary of Study Intervention(s) Administered 82

4.2 SARS-CoV-2 Challenge Virus ... 85
4.2.1 Provenance of the wild type SARS-CoV-2 challenge virus ... 85
4.2.2 Justification for treatment dose and route of administration ... 85
4.2.3 Supply and accountability of challenge virus ... 85
4.2.4 Storage of challenge virus ... 85
4.2.5 Preparation and administration of challenge virus ... 86
4.3 Antiviral Treatment (Remdesivir as Pre-emptive Medication) ... 87
4.4 Dose Preparation ... 87
4.5 Early “Rescue” Therapy ... 87
4.6 Dose Preparation ... 88
4.7 Handling, Storage, and Accountability ... 88
4.8 Randomisation and Blinding ... 89
4.9 Study Intervention Compliance ... 89
4.10 Concomitant and Prior Therapy ... 89
4.11 Dose Escalation, De-escalation and Confirmation ... 91
4.12 Temporary Discontinuation/Temporary Delay in Enrolment ... 98
4.12 Participant Replacement Strategy ... 98
4.13 Stopping Rules ... 98

5. Study Assessments and Procedures ... 100
5.1 Medical and Medication History ... 100
5.2 Demographics ... 100
5.3 Height, Weight and Body Mass Index (BMI) ... 100
5.4 Alcohol Breath Testing ... 101
5.5 Physical Examinations ... 101
5.6 Vital Signs ... 101
5.7 Temperature ... 102
5.8 Radiology ... 102
5.9 Echocardiogram ... 103
5.10 Electrocardiogram ... 103
5.11 Lung Function ... 103
5.11.1 Spirometry ... 103
5.11.2 Forced oscillation technique (FOT) ... 104
5.12 Patient Health Questionnaire (PHQ-9) and Generalised Anxiety Disorder (GAD-7) Questionnaire ... 104
5.13 Participant Symptom Diary Card – Clinical scores ... 104
5.14 University of Pennsylvania Smell Identification Test (UPSIT) ... 106
5.14 Cognitive Testing ... 106
5.15 Urinalysis ... 107
5.15 Urine Drugs of Abuse and Nicotine Test ... 107
5.16 Pregnancy Test ... 107
5.17 Blood Samples ... 107
5.17.1 Safety blood samples ... 108
5.17.2 Immunology and sero-suitability to challenge virus ... 108
5.18 Respiratory Samples ... 108
5.18.1 Viral load ... 109
5.18.2 Respiratory pathogen screen ... 109
5.18.3 SARS-CoV-2 confirmation of infection for intervention treatment ... 109
5.18.4 SAR-CoV-2 confirmation of infection endpoint analysis ... 109
5.18.5 SARS-CoV-2 discharge process ... 109
5.18.6 Post-discharge process ... 110

5.19 Environmental Sampling 111
5.20 Mask wearing sampling 111
5.21 Exploratory research 111
5.22 Optional Exploratory Non-Interventional Study of Wearable Device 112

6. Recording of Adverse Events and Serious Adverse Events 113
6.1 Time Period and Frequency for Collecting AE and SAE Information 113
6.2 Method of Detecting AEs and SAEs 113
6.2.1 Follow-up of AEs and SAEs 113
6.2.2 Regulatory reporting requirements of SAEs 113
6.3 Reporting of events related to REGEN-COV 114
6.3.1 Pregnancy 114
6.3.2 Disease-related events and/or disease related outcomes not qualifying as AEs or SAEs 115
6.3.3 Treatment of overdose 115
6.4 Safety Oversight Procedures 115
6.4.1 Procedures to be followed in the event of abnormal findings 115
6.4.2 Ongoing and interim safety reviews 115
6.4.3 Safety holding rules 116
6.4.4 Group holding rules 116
6.5 Data Safety Monitoring Board 117
6.6 Trial Steering Committee (also known as Medical Oversight Committee) 117
6.7 Human Challenge Steering Committee 118
6.8 Pharmacokinetics 118
6.9 Pharmacodynamics 118

7. Statistical Methods and Planned 119
7.1 Study Analysis Sets 119
7.2 Subgroup Analysis 119
7.3 Sample Size 120
7.4 Cohort and Dose Escalation 120
7.5 Interim Statistical Analysis 121
7.6 Statistical Analysis Plan 121
7.6.1 Subject Accountability 122
7.6.2 Protocol deviations 122
7.6.3 Demographic and baseline characteristics 122
7.7 Primary Endpoint Analysis 122
7.8 Secondary Endpoint Analysis 123
7.9 Exploratory Endpoints 123
7.10 Safety Analysis 123

8. Appendices: Supporting Documentation and Operational Considerations 125
Appendix 1: Regulatory, Ethical, and Study Oversight Considerations 125
8.1.1 Regulatory and ethical considerations 125
8.1.2 Financial disclosure 125
8.1.3 Funding 126
8.1.4 Informed Consent Process 126
8.1.5 Confidentiality 127
8.1.6 Data protection 127
8.1.7 Indemnity 127
8.1.8 Sponsor 127

8.1.9 Audits ... 127
8.1.10 Sample and data storage and usage ... 128
8.1.11 Dissemination of clinical study data ... 128
8.1.12 Data quality assurance ... 128
8.1.13 Source documents ... 129
8.1.14 Study discontinuation ... 129
8.1.15 Study management and governance ... 129
8.1.16 Publication policy ... 130
Appendix 2: Clinical Laboratory Tests ... 132
Appendix 3: Toxicity Grading Scale for Lab AEs ... 134
Appendix 4: Adverse Events: Definitions and Procedures for Recording, Evaluating, Follow-up, and Reporting ... 136
8.4.1 Adverse event ... 136
8.4.2 Serious adverse event ... 136
8.4.3 Potential adverse events related to SARS-CoV-2 infection ... 138
8.4.4 Recording, assessment and follow-up of AE and/or SAE ... 138
8.4.5 Reporting of SAEs ... 146
8.4.6 Adverse reactions to non-IMPS ... 147
8.4.7 Post-study AEs and SAEs ... 147
8.4.8 Pregnancy ... 147
Appendix 5: Normal Ranges ... 149
Appendix 6: Abbreviations ... 150

9. References ... 153

1. Protocol Synopsis

1.1 Protocol Summary

This is a dose optimisation study in healthy adults aged 18-30 who will be experimentally inoculated with SARS-CoV-2. The aim is to cause PCR-confirmed upper respiratory infection in the majority of challenged individuals with minimal or no illness, providing data on the course of COVID-19 and the immune response to SARS-CoV-2 infection. This will establish an optimised dose and study design that will then be used to evaluate the efficacy of treatment and vaccine candidates plus level and duration of immune protection in follow-on trials.

Title of the study	A dose finding human experimental infection study in healthy subjects using a GMP-produced SARS-COV-2 wild type strain (SARS-CoV-2 Characterisation Study)
Protocol number	COVHIC001
Estimated Timelines:	January 2021 – June 2022
Sites/Location:	A single centre study carried out in a high containment unit at the Royal Free Hospital, London. Follow Up visits are being carried out at the Queen Mary BioEnterprises (QMB) Innovation Centre, London
Challenge virus:	SARS-CoV-2, intranasally
Potential Intervention treatment	Pre-emptive therapy - VEKLURY™ (Remdesivir)<br>Rescue therapy – REGEN-COV™ (Regeneron Monoclonal Antibody cocktail)
Study Outline	This is a dose optimisation study in which escalating or de-escalating doses of wild-type SARS-CoV-2 (starting at $1x10^1$ $TCID_{50}$) will be given via nasal administration to different groups of volunteers in order to achieve a ≥50% attack rate (ideally between 50% and 70%), as determined by positive qPCR detection (viral load ≥LLOQ) in respiratory secretions (mid-turbinate swabs and/or throat swabs) at two consecutive 12 hourly time points.<br>For initiation of each new dose level, up to 10 subjects will be included in the first cohort, with a sentinel group of 3 subjects initially assessed for safety and infectivity by the investigators, before proceeding with subsequent inoculations.<br>A Data Safety Monitoring Board will review safety and quantitative

	virology after each cohort and will recommend continuation, dose escalation or de-escalation based on emergent data. A Trial Steering Committee (also known as the Medical Oversight Committee) will provide overall supervision of the project.<br><br>Subjects may be given SARS-CoV-2 intervention treatment, depending on the stage of the study.<br><br>Initially, subjects, upon evidence of infection, will be treated with a pre-emptive antiviral IV Remdesivir for five days.<br><br>Subsequent cohorts, may include one of the following treatment plans:<br>• Pre-emptive therapy, upon evidence of infection (Remdesivir),<br>• Early “rescue” therapy, once disease progression criteria are met.<br>• No pre-emptive or “rescue” therapy<br><br>Alternative treatments may be included that have shown compelling evidence of efficacy against SARS-CoV-2, if available, such as the IV REGEN-COV monoclonal antibody cocktail.<br><br>Decisions on treatment plans for cohorts of subjects will be made by the CI/PI, and in discussion with the DSMB<br><br>Subjects will remain in quarantine for a minimum of 14 days in a quarantine clinical trials unit (from inoculation), and until these criteria are met:<br>• For subjects with evidence of infection:<br>o Two consecutive swabs with qPCR Ct >33.5 AND negative culture.<br>o Lateral flow tests may be used in place of culture, if culture data is not available<br>o A qualitative PCR may be used in place of quantitative PCR, if the latter is not available.<br>o At the PI’s discretion if protracted quarantine is deemed to be causing harm to the participant’s mental or physical health and no viable virus is detected by culture or a lateral flow test is negative.<br>• For non-infected subjects:<br>o 2 consecutive swabs with undetectable virus by PCR only prior to discharge.
Population	Sero-suitable healthy male and female volunteers 18-30 years of age (inclusive) with no known risk factors for severe COVID-19
Number of Subjects	up to 90

Duration of Participation	The total duration of study for participants is estimated approximately 377 days from admission to the completion of last follow up visit.
Inclusion Criteria	Healthy adults ages 18-30 years who are seronegative for SARS COV-2 antibodies and have no known risk factors for severe COVID-19
Exclusion Criteria (specific to the nasal challenge)	Any significant abnormality altering the anatomy of the nose or nasopharynx, clinically significant history of epistaxis (nose bleeds) or any nasal or sinus surgery within six months of inoculation
Estimated duration of the study	The Sponsor estimates that the study will require approximately 52 weeks from the time the first participant signs the informed consent until the last study related follow up.
Primary Objective /Endpoint	• To identify a safe and infectious dose of wild type SARS-CoV-2 in healthy volunteers, suitable for future intervention studies, that:<br>• has an acceptable safety profile as measured by:<br>o Occurrence of Adverse Events (AEs) within 30 days post-viral challenge (Day 0) up to Day 28 follow up.<br>o Occurrence of Serious Adverse Events (SAEs) from the viral challenge (Day 0) up to Day 28 follow up.<br>• induces laboratory confirmed infection in ≥50% of participants (ideally between 50% and 70%). Laboratory confirmed infection is defined by:<br>o Two quantifiable greater than lower limit of quantification (viral load ≥LLOQ) RT-PCR measurements from mid turbinate and/or throat samples, reported on 2 or more consecutive timepoints, starting from 24 hours post-inoculation and up to discharge from quarantine.
Secondary Objectives/endpoint	• To further assess SARS-CoV-2 viral infection rates in upper respiratory samples by qRT-PCR and cell culture<br>• To assess the incidence of symptomatic SARS-CoV-2 infection<br>• To assess the SARS-CoV-2 viral dynamics in upper respiratory samples (AUC, peak, duration, incubation period)<br>• To assess the SARS-CoV-2 induced symptoms (Sum, AUC, peak, peak daily, frequency)<br>• To assess the incidence of SARS-CoV-2 illness (Upper Respiratory Tract illness [URTI], Lower Respiratory Tract illness [LRTI], Systemic Illness (SI), Febrile Illness [FI], grade 1, 2 & 3

	symptoms)<br><br>A full description of secondary endpoints is detailed in sections 2 and 7.
Exploratory/Tertiary Objectives / Endpoints	• To explore the safety of the wild type SARS-CoV-2 human challenge model (smell, cognition, pulmonary changes [CT, spirometry, FOT], safety laboratory tests, blood type, concomitant medications)<br>• To explore SARS-CoV-2 viral infection rates in saliva, by qRT-PCR and cell culture<br>• To explore the SARS-CoV-2 viral dynamics in saliva, by qRT-PCR and cell culture (AUC, peak, duration, incubation period)<br>• To explore the host-pathogen relationship in the SARS-CoV-2 human challenge model (including humoral and cellular immunity, proteomics, transcriptomics, host and viral genomics, microbiome and systems biology)<br>• To explore the Minimal Clinically Important Difference (MCID) in instrument change (e.g. Symptom diary cards)<br>• To explore environmental contamination in the SARS-CoV-2 human challenge model (quantitation and detection of virus in air sampling, exhaled breath and surface swabbing)<br><br>A full description of exploratory/tertiary endpoints is detailed in sections 2 and 7.
Sample size	The primary objective of the study is to identify a safe and infectious dose of wild type SARS-CoV-2 in healthy volunteers, suitable for future intervention studies. No formal sample size calculation has been performed for this early-stage dose finding study. However, a sample size of up to an expected 30 subjects for a dose level and treatment regimen (made up of 3 cohorts of up to an expected 10 subjects, including the dose expansion cohort) with a total of up to 90 subjects overall, is felt sufficient to meet the primary objective of escalating/expanding the dose in a safe manner whilst providing information on the attack rate.
Statistics	The following analysis sets are defined for this study:<br><br>• **Full Analysis Set (FAS)** is defined as all subjects that are inoculated with wild-type SARS-COV-2 challenge virus. The FAS will be considered as the primary analysis set for all primary, secondary and exploratory endpoints.<br><br>• **Safety Analysis Set** is defined as all subjects that are inoculated with wild-type SARS-COV-2 challenge virus. The Safety Analysis Set is identical to FAS and will be used for all

	safety endpoints.<br>• **Per Protocol (PP) Analysis Set** is defined as all FAS subjects that are sero-suitable, who have no major protocol deviations and who complete the quarantine period up to the final day of quarantine (study Day 14). The PP Analysis Set will be considered as the secondary analysis set for pre-specified primary, secondary and exploratory endpoints.<br>Membership of subjects in each analysis set will be determined at a planned data review meeting (DRM), prior to any analysis and database lock. A 'Laboratory confirmed infected' subgroup will be identified and will be presented for certain pre-specified analyses.<br>Primary, secondary and exploratory endpoints will be summarised using descriptive statistics. Continuous variables will be summarised using number of observations, mean (and/or geometric mean, where applicable), standard deviation, standard error, median, lower quartile, upper quartile, minimum and maximum values. Categorical variables will be summarised using proportions (counts and percentages). A 95% confidence interval may be presented for certain pre-specified endpoints.<br>Methods for dealing with missing data in the study analysis will be described within the Statistical Analysis Plan (SAP).
Keywords	SARS-CoV-2, COVID-19, immune, virus, viral challenge, viral lung disease, infection

A list of abbreviations used in this document can be found in Appendix 6.

1.2 Schedules of Activities (SoA)

Table 1 Schedule of Activities

Study Phase		Inpatient Quarantine																										
Study Day	Screening Day -90 to Day -3	D-2	D-1	Day 0 Pre	D0 Challenge	Day 0 Post	D1	D2	D3	D4	D5	D6	D7	D8	D9	D10	D11	D12	D13	D14	Extended stay days	Day 15. - 27	Day 28 (+/- 3 days)	Day 90 (+/- 7 days)	Day 180 (+/- 14 days)	Day 270 (+/- 14 days)	Day 360 (+/- 14 days)	Early withdrawal
Written consent	X*	X																										
Eligibility criteria (+)	X	X[k*]		X																								
Medical & medication history	X																											
Change in medical and medication history		X																					X	X	X	X	X	X
Demographics	X																											
Height & weight, BMI (a)	X	X[k]																										
Patient Health Questionnaire (PHQ-9)	(X)	X[k]																	X		X[W]							
Generalised Anxiety Disorder Questionnaire (GAD-7)	(X)	X[k]																	X		X[W]							
Alcohol breath test	X	X[k]																					X				X	X
Urinalysis	X	X[k]								X			X		X					X			X	X	X	X	X	X
Urine drugs of abuse & nicotine screen	X	X[k]																					X				X	X

Study Phase	Screening Day -90 to Day -3	Inpatient Quarantine																				Extended stay days							
Study Day		D-2	D-1	Day 0 Pre	D0 Challenge	Day 0 Post	D1	D2	D3	D4	D5	D6	D7	D8	D9	D10	D11	D12	D13	D14		Day 15. - 27	Day 28 (+/- 3 days)	Day 90 (+/- 7 days)	Day 180 (+/- 14 days)	Day 270 (+/- 14 days)	Day 360 (+/- 14 days)	Early withdrawal
Urine pregnancy test	X			X																X			X				X	X
Complete physical examination	X		X^k							X			X		X					X	$(X)^W$		X	X	X	X	X	X
Directed physical examination (inc nasal)				X		X	X	X	X		X	X		X		X	X	X	X		X							
Vital signs (HR, RR, SBP, DBP, SpO_2 (b) (n)	X	(X)	QDS		QDS		QDS	QDS	QDS	QDS	QDS	QDS	QDS	QDS	QDS	QDS	QDS	QDS	QDS	QDS^x	QDS^x		X	X	X	X	X	X
Tympanic temperature (n)	X	(X)	QDS		QDS		QDS	QDS	QDS	QDS	QDS	QDS	QDS	QDS	QDS	QDS	QDS	QDS	QDS	QDS^x	QDS^x		X	X	X	X	X	X
Symptom diary cards		(X)	TDS		TDS		TDS	TDS	TDS	TDS	TDS	TDS	TDS	TDS	TDS	TDS	TDS	TDS	TDS	TDS^x	TDS^x							
Smell Test (UPSIT)			X^u				X^u			X^u			X^u			X^u			X^u		(X)		X^u	X^u	X^u	X^u	X^u	
Cognitive Tests			X^v	X^v			X^v	X^v	X^v	X^v	X^v	X^v	X^v	X^v	X^v	X^v	X^v	X^v	X^v	X^v			X^v	X^v	X^v	X^v	X^v	
Chest X-ray (p)			X^k																									
Lung CT scan											X^0	X^0				X^0	X^0											
Echo (p)			X^k																									
12-lead ECG (n)	X		X^k	X			X	X	X	X	X	X	X	X	X	X	X	X	X	X	(X)		X	X	X	X	X	X
Spirometry (c)	X		X^k	X			X	X	X	X	X	X	X	X	X	X	X	X	X	X	(X)		X	X	X	X	X	X
FOT (c)			X^k	X			X	X	X	X	X	X	X	X	X	X	X	X	X	X	(X)		X	X	X	X	X	X
Telephone calls(r)																						X						
**Product Administration**																												

Study Phase		Inpatient Quarantine																										
**Study Day**	**Screening Day -90 to Day -3**	**D-2**	**D-1**	**Day 0 Pre**	**D0 Challenge**	**Day 0 Post**	**D1**	**D2**	**D3**	**D4**	**D5**	**D6**	**D7**	**D8**	**D9**	**D10**	**D11**	**D12**	**D13**	**D14**	**Extended stay days**	**Day 15. - 27**	**Day 28 (+/- 3 days)**	**Day 90 (+/- 7 days)**	**Day 180 (+/- 14 days)**	**Day 270 (+/- 14 days)**	**Day 360 (+/- 14 days)**	**Early withdrawal**
Challenge Virus inoculation					X																							
Administration of SARS-CoV-2 intervention treatment									$(X)^{y}$																			
**Collection of blood samples**																												
Serum β-HCG pregnancy test (all females) (e)			X^{k*}																									
HIV, Hepatitis A, B, & C	X		X^{k}																									
Thyroid function test	X		X^{k}																									
Coagulation (including d-dimer)	X		X^{k}				X	X	X	X	X	X	X	X	X	X	X	X	X	X	(X)		X	X	X	X	X	X
Haematology (f)	X		X^{k}	X			X	X	X	X	X	X	X	X	X	X	X	X	X	X	(X)		X	X	X	X	X	X
Biochemistry	X		X^{k}				X	X	X	X	X	X	X	X	X	X	X	X	X	X	(X)		X	X	X	X	X	X
Cardiac enzymes (including high sensitivity Troponin)	X		X^{k}				X	X	X	X	X	X	X	X	X	X	X	X	X	X	(X)		X	X	X	X	X	X
Blood - Antibodies SARS-CoV-2	X		X^{k}																	X			X	X	X	X	X	X
Blood - plasma markers (h) (g)			X^{k}	X			X	X	X	X	X	X	X	X	X	X	X	X	X	X			X	X	X	X	X	
Blood paxgene RNA			X^{k}	X		X	X	X	X	X	X	X	X	X	X	X	X	X	X	X			X	X	X	X	X	

Study Phase	Screening	Inpatient Quarantine																			Extended stay days							
Study Day	Day -90 to Day -3	D-2	D-1	Day 0 Pre	D0 Challenge	Day 0 Post	D1	D2	D3	D4	D5	D6	D7	D8	D9	D10	D11	D12	D13	D14		Day 15. - 27	Day 28 (+/- 3 days)	Day 90 (+/- 7 days)	Day 180 (+/- 14 days)	Day 270 (+/- 14 days)	Day 360 (+/- 14 days)	Early withdrawal
Blood Lithium Heparin – PBMCs, other blood cells, +/- plasma			X[k]						X				X			X				X			X	X	X	X	X	
**Collection of respiratory and environmental. Samples**																												
Nasopharyngeal swab- Respiratory pathogen screen - e.g. Biofire (i)	(X)	X	(X)[m]																									
Nasopharyngeal swab- tolerance test	X																											
Nasosorption – immunology & virology (q)			BD	X		X	BD	BD	BD	BD	BD	BD	BD	BD	BD	BD	BD	BD	BD	BD[x]	BD[x]		X	X	X	X	X	X
Saliva-virology (j) (g)			X				BD	BD	BD	BD	BD	BD	BD	BD	BD	BD	BD	BD	BD	BD[x]	BD[x]		X					X
Throat FLOQ swab - Virology (j) (g)			X				BD	BD	BD	BD	BD	BD	BD	BD	BD	BD	BD	BD	BD	BD[x]	BD[x]		X	(X)	(X)			X
Mid turbinate FLOQ swab - Virology (j) (g) (m)			X				BD	BD	BD	BD	BD	BD	BD	BD	BD	BD	BD	BD	BD	BD[x]	BD[x]		X	(X)	(X)			X
Mid turbinate and/or nasopharyngeal swab for cells for RNA (g)			X				X		X		X		X			X				X								
Mask wearing sampling			X				X	X	X	X	X	X	X	X	X	X	X	X	X	X	(X)							

Study Phase / Study Day	Screening Day -90 to Day -3	Inpatient Quarantine D-2	D-1	Day 0 Pre	D0 Challenge	Day 0 Post	D1	D2	D3	D4	D5	D6	D7	D8	D9	D10	D11	D12	D13	D14	Extended stay days	Day 15. - 27	Day 28 (+/- 3 days)	Day 90 (+/- 7 days)	Day 180 (+/- 14 days)	Day 270 (+/- 14 days)	Day 360 (+/- 14 days)	Early withdrawal
(s)																												
Environmental viral sampling (t)			X				X	X	X	X	X	X	X	X	X	X	X	X	X	X	(X)							
**Safety Assessments**																												
AE recording	X	←→ (D-2 to Day 360)																										X
SAE recording	X	←→ (D-2 to Day 360)																										X
Concomitant medications	X	←→ (D-2 to Day 360)																										X

Table 2 Key notes for SoA

X	Once
BD	Twice Daily.<br>The timing of the baseline assessment will be the guide to establish the windows for subsequent measurements. For scheduling purposes, the baseline assessment will be defined as the first day when BD measurements are performed. Subsequent sampling/measures will be performed at the same time ± 1 hour.
TDS	Three times daily.<br>The timing of the baseline assessment will be the guide to establish the windows for subsequent measurements. For scheduling purposes, the baseline assessment will be defined as the first day when TDS measurements are performed. Subsequent sampling/measures will be performed at the same time ± 1 hour.
QDS	Four times daily.<br>The timing of the baseline assessment will be the guide to establish the windows for subsequent measurements. For scheduling purposes, the baseline assessment will be defined as the first day when QDS measurements are performed. Subsequent sampling/measures will be performed at the same time ± 1 hour.

T	To determine tolerance of the procedure only (sample will not be tested).
+	Only the applicable Inclusion/Exclusion criteria will be reviewed at each time point.
a	Height will be taken at Screening only.
b	Vital signs will be taken at the same time each day (± 1 hour).
c	Lung function measures will be performed at the same time each day during quarantine (± 1 hour).
d	A serum follicle stimulating hormone (FSH) test will be performed in all post-menopausal women
e	Blood serum pregnancy test (ß-HCG) will be performed in all female subjects
f	Blood will be drawn under non-fasted conditions.<br>Repeat bloods may be drawn under fasted conditions if a lipid profile (triglyceride) or glucose is required (at PI discretion).
g	Samples for related exploratory research
h	Virus serology will be performed to determine eligibility and seroconversion.
i	Nasopharyngeal swab for respiratory virus screen to assess for the presence of other respiratory viruses; if found positive the subject will not be eligible for the current quarantine.
j	Post inoculation Nasal virology samples will be collected and used for RT-qPCR and viral culture assay (as appropriate). Samples may be used for related exploratory research
k + k*	Can be performed on Study Day -2 or Study Day -1. *Special Note:* k* Serum pregnancy test should be performed on either day -2 or -1, prior to Urine Pregnancy Test on Day 0 (pre-inoculation).
l	Study specific consent may occur on admission, providing all required eligibility information has been collected through the HRA approved study specific Screening process
m	If for the study, mid turbinate FLOQ swabs are not available (e.g. national shortage), nasopharyngeal FLOQ swabs may be taken as a viable alternative. The study should endeavor to be consistent in swab type across all the participants.
n	Assessments may additionally be continuously monitored (e.g. core temperature; vital signs (HR, RR, SBP, DBP, SpO_2), activity, sleep, ECG) as per Section 5.
o	CT scan to be performed in all participants on day 5 or 6 and Day 10 or 11 dependent upon scanner availability; CT scan to be performed only in participants with PCR confirmed infection or symptoms consistent with COVID-19.
p	Chest X-Ray and ECHO will be obtained on Admission to quarantine on Day-2/Day-1. Radiology assessments will not be repeated in the scenario where a subject is reinvited to attend a later Admission/Quarantine.
q	Two sets of nasosorption will be taken at each time point. Each set consists of 2 nasosorptions, 1 for each nostril (i.e. total of 4 nasosorption devices per time point). Timing between sampling of the sets should be at least 5 minutes apart. Samples may be used for: cytokines/chemokines, sIgA, and virology, as well as stored for future usage
r	Telephone calls with participants every 2 to 3 days
s	Participant will be asked to wear a single-use facemask for 60 – 90 minutes, 1 – 3 times a day to capture exhaled virus
t	Environmental viral sampling may be performed in rooms up to twice a day

u	The UPSIT is designed to be self-administered after explanation of the test by study staff and will be performed once before virus inoculation and then at least every third day starting from Day 1, though the test can be conducted more frequently at the discretion of the PI/study physician. If at Day 28 anosmia has subsided and smell has returned the UPSIT test can stop. If anosmia is still present UPSIT should continue until resolution or the end of the study.
v	Once daily, at the same time each day (+/- 1 hour) during quarantine and similar timing where possible during follow-up visits
w	Before discharge from quarantine each participant will undergo a complete physical examination (the date of this may vary dependent upon length of stay) and repeat GAD-7 and PHQ-9 questionnaires once.
x	Procedures should be performed at the appropriate scheduled timings, up until the point of discharge.
y	Subjects may be given SARS-CoV-2 intervention treatment, depending on the stage of the study. Treatment plans for subjects include one of the following:<br>• Pre-emptive therapy, upon evidence of infection (Remdesivir, once daily IV for 5 days)<br>• Rescue therapy, once disease progression criteria is met (e.g., REGEN-COV, once IV)<br>• No pre-emptive or rescue therapy
Notes:	Parenthesis indicates the assessment may be optional, or at the PI's discretion.<br>Study specific consent may occur on the day of admission, providing all required eligibility information has been collected and the study fully explained to the participant through earlier screening visits and remote appointments process<br>For all subjects QDS/TDS assessments on Day 0, the first assessment will be pre-virus challenge.<br>The PI may perform additional safety assessments as required.<br>Where any nasal sampling time points occur together, the order of sampling will typically be (1) Nasosorptions followed by (2) mid turbinate swab (3) Nasopharyngeal swab.

2. Background to Research

In the current COVID-19 emergency, controlled SARS-CoV-2 human infection has the potential to accelerate our understanding of pathogenesis, induction of immunity and immune mechanisms of resistance to disease, as well as a means to rapidly test novel diagnostics, treatments and vaccines, especially between waves of the pandemic, when occurrence of natural disease is relatively uncommon. Specifically, it will enable efficient early-stage testing of the many vaccine candidates in development so that the most promising can go forward quickly enough to large-scale field efficacy trials to meaningfully tackle the ongoing pandemic. Early cost-benefit modeling suggested that a SARS-CoV-2 human infection challenge study used to test vaccine efficacy could accelerate approval, potentially averting an additional 1.1 million infections and 8,000 deaths in the USA alone, compared to the next best clinical trial design (i.e. field trials with natural infection) (Berry et al., 2020). While this is not needed for the first generation of SARS-CoV-2 vaccines that are now becoming available, these types of studies remain important. Although first generation vaccines are disease-modifying, their transmission-blocking potential and longevity of protection are unknown and will need to be tested and improved upon. Greater efficacy in older adults and other high-risk groups, will almost definitely be required and it still remains unclear whether first generation vaccines will protect against severe disease as well as mild/moderate illness, the early trials having only enrolled small numbers in each of these subgroups. Furthermore, antivirals and monoclonal antibodies will still be needed for those who remain unvaccinated, become infected in spite of vaccination or cannot mount effective immune responses. In addition, the controlled nature of these studies can provide definitive answers to questions about protective immunity that cannot be achieved with episodic and sporadic natural infections, which will be critical for effective public health strategy. These studies have had expert (Eyal et al., 2020; Plotkin and Caplan, 2020) and public support (Gbesemete et al., 2020), but to reach these diverse goals, a safe and reproducible SARS-CoV-2 infection model must be first established.

No alternative study design exists that can so rapidly provide clinical readouts using small numbers of volunteers in a controlled setting. With the roll-out of first-generation vaccines many middle-aged and older adults will be immune (at least for a period of time) and natural transmissions will fall. Field studies will therefore become increasingly unfeasible and only trials that can support extremely large sample sizes will be conducted. This includes household transmission studies, which also lack control (due to unmeasurable variations in viral strain, inoculum size, environmental and host factors) and make conclusive interpretation of resulting data highly challenging. In any case, these types of studies cannot be used to accelerate testing of vaccines or prophylaxis due to the sporadic and unpredictable nature of transmission.

2.1 Why is the COVID-19 challenge model so important?

There are many aspects of COVID-19 infection that scientists still don’t understand. The human challenge study may be the only way to get this information. We believe that the information

obtained in the challenge study will have important public health benefits and play a significant role in the ongoing management of the pandemic.

1. The “incubation period” of COVID-19 is the time from initial coronavirus exposure to the onset of symptoms and when people are most infectious. A human challenge study is the only way to accurately measure how long the incubation period is, and will assist and inform future track and trace efforts.
2. A human challenge study will allow us to accurately measure how long people are infectious, from first exposure to the virus being cleared. This will determine exactly how long self-isolation (quarantine) periods should be after exposure.
3. Reinfection means a person was infected (got sick) once, recovered fully, and then later became infected for a second time. Based on what we know from similar viruses, some people could catch COVID-19 more than once. A human challenge study will help answer a number of important questions on re-infection, including how long natural immunity can protect people from re-infection; what makes people more at risk of re-infection; and whether re-infected people can spread the virus to others. A human challenge study will enable future testing of vaccines and antiviral treatments. A key question for new vaccines is whether they stop people carrying and spreading the virus as well as preventing symptoms.
4. Many people infected with coronavirus have no symptoms (asymptomatic) and are a major cause of spreading the infection because they are unaware they are infected. A human challenge study will allow researchers to learn much more about asymptomatic infection and importantly if vaccines can prevent it.

- **Rationale:** SARS-CoV-2 human infection challenge provides critical knowledge about the course of infection to guide public health measures, and accelerate vaccine and therapeutics development. This is achieved enabling small, rapid efficacy studies to compare and triage novel candidates in the face of increasing limitations to implementing field efficacy trials.
- **Why conduct human challenge now vaccines are available?** First generation SARS-CoV-2 vaccines are now available. However, even with effective vaccines, SARS-CoV-2 will almost definitely become endemic and repeated outbreaks will occur. The establishment of this model is therefore still critical to accelerate the development of next-generation SARS-CoV-2 vaccines, test antivirals and monoclonal antibodies, understand immunity against coronaviruses, and prepare for viral mutation or future pandemics.
- **Why are the first-generation vaccines not good enough?** There is currently no antiviral treatment that works well to treat severe COVID-19. Improved vaccines are also still required that:

    1. definitively block transmission
    2. are deliverable to low resource settings (without cold chain or facilities/manpower to provide injections)
    3. can be administered with a single or smaller dose

4. can provide cross-strain protection that will be needed as the virus mutates.

There is significant interest from vaccine producers with novel vaccine candidates not yet in field trials to use the challenge model to quickly determine proof of concept for their vaccine and/or provide supportive efficacy data to apply for emergency licensure of the vaccine.

- **Why do we need to know about immune protection?** Human challenge will answer critical questions about what type or level of immunity (after natural infection or vaccination) confers protection and how long the protection will last. Knowing this will have immediate public health impacts, including risk to individuals of infection/re-infection, choice of vaccines, and decisions on timing of vaccination and re-vaccination.
- **Why not do field trials instead?** Field trials of all kinds will become increasingly difficult and slow to conduct once widespread vaccination begins and natural infections decrease. Human challenge studies may therefore become the only way to test efficacy for most candidates (with young adults being last and least likely to be vaccinated) and to definitively understand the strength of immune protection quickly enough to make an impact on public health strategy.

- **What will this first study immediately enable?** This virus characterisation study is the key to enabling dependent studies. The following are already funded and will follow on immediately once we have preliminary results:
    1. The Vaccines Taskforce is funding studies to compare vaccine candidates head-to-head against licensed vaccine(s). These will identify next-generation vaccines, formulations or dosing regimens that better address global needs and are planned to start in May 2021.
    2. The Department of Health and Social Care have funded a study led by the University of Oxford to show whether previously-infected people are immune to re-infection and what level of antibodies equates to protection.
    3. The Wellcome Trust have pledged funding of the manufacture of further SARS-CoV-2 challenge virus stocks that will be shared freely with investigators around the world to boost this research.
- **How is this research relevant to patients?** While young healthy adults may not fully recapitulate high risk groups, they provide a benchmark for optimal protective immunity and are highly suitable for antiviral and monoclonal antibody testing. People in this age group are also likely to be the main drivers of continuing pandemic transmission once older adults are vaccinated and will be the main target for vaccines that aim to prevent asymptomatic transmission by reduction of viral shedding in the upper respiratory tract.
- **What are the risks to volunteers?** Young healthy adults in the 18-30 year old age are at low risk of severe outcomes following COVID-19. As of 6th November 2020 in England and Wales, 95 individuals in this age range have died due to COVID-19, but most had risk factors. Risk of hospitalisation in this age group (without accounting for co-morbidities) has recently been estimated at 0.08-0.39%. Protracted symptoms ("long COVID") can occur but are less common in young people with mild disease, almost

always resolving within 4 months. This study is fully indemnified with no upper limit if a participant becomes unexpectedly unwell.

- **What risk mitigations are in place?** Since there may have been rare cases of severe disease in 18-30 year olds with no underlying risk factors, the following risk mitigations will be put in place to maximally limit the possibility of severe outcomes:
  1. The first-in-human virus characterisation study will be performed by an **expert team** with extensive respiratory virus human challenge experience.
  2. **Inclusion/exclusion criteria** will select the first participants with the lowest risk based on objective UK data with regular review as new data emerges. However, the DSMB will formally assess the criteria to maximise inclusion as soon as the challenge infection has been shown to be safe and well-tolerated.
  3. Dose finding will be performed in a cautious staged manner, **starting with the lowest reliably quantifiable dose** of 10 TCID50 using a GMP-manufactured virus.
  4. Intervention treatment (e.g. pre-emptive or rescue therapy) will be used as appropriate, when compelling evidence for efficacy of specific treatments exists and such treatments are available for use. Pre-emptive treatment occurs at a defined timepoint related to infection diagnosis; “rescue” therapy occurs at a timepoint related to disease progression. Participants will be **quarantined at the Royal Free Hospital** after inoculation and continuously monitored for unexpectedly severe symptoms. If necessary, they will have full access to world-class NHS care by a highly experienced clinical team.
  5. Participants will be **followed up for 12 months** to monitor for protracted symptoms and managed appropriately (with specialist referral if necessary).

2.2 Research Strategy

The two endpoints in a human challenge model which could provide hard evidence in evaluation of vaccines and other interventions are (i) virological (i.e. quantified evidence of infection of the participant) and (ii) disease-based (i.e. a measure of symptom intensity in the participant). A significant proportion of natural SARS-CoV-2 infections are asymptomatic, and at the present time the force of infection, probability of disease given infection, and the host and microbial factors which determine progression from infection to disease are not defined. In view of this, and so that the risk of associated morbidity is minimized, the Key Protocol & Research Contributors agreed that measurement of viral concentrations within the upper respiratory tract (URT) would provide the safest and most reliably-defined endpoint, rather than a measured symptom/disease-based read-out.

The current study design is therefore a dose titration to identify the virus dose that is safe and well tolerated while resulting in reproducible levels of SARS-CoV-2 viral infection in the URT and immune responses in inoculated subjects, without necessarily inducing symptoms.

In order to develop SARS-CoV-2 human challenge studies, a comprehensive review of potential safety concerns and risk mitigation is necessary. To ensure the safety of the challenge model participants, it is also paramount that the challenge virus be produced compliant with Good

Manufacturing Practice (GMP) and the manufacturing and testing processes subject to detailed regulatory review and approval. Inclusion criteria must take account of the population risk for the selected group (including age, demographics and co-morbidities). The challenge study must be conducted in a fully equipped clinical study unit with appropriate containment conditions and under the care of experienced medical and nursing staff. Additionally, research subjects must be closely monitored using state of the art medical monitoring including CT imaging to sensitively identify changes in the lung.

2.3 Virology of SARS-CoV-2

SARS-CoV-2 is an enveloped positive sense single-stranded RNA virus with a genome of approximately 29,000 nucleotides in length. SARS-CoV-2, along with SARS1 (the cause of SARS in 2002-3), are members of the genus *Betacoronavirus,* subgenus *Sarbecovirus*. SARS-CoV-2 shows 79% nucleotide similarity with SARS1 virus but is suggested to be most closely related to the horseshoe bat *Sarbecovirus*, RaTG13, having diverged from this around 40-70 years ago. It is likely that SARS-CoV-2 has been circulating in bats and intermediate species (possibly the pangolin) before crossing into humans in Hubei province of China in late 2019.

SARS-CoV-2 enters a human host cell by binding to the human angiotensin-converting enzyme 2 (ACE2) receptor with the viral spike (S) protein via the spike receptor binding domain (RBD). Since January 2020, SARS-CoV-2 has spread globally. It has a relatively low mutation rate of around 2.5 mutations/genome/month (similar to other coronaviruses), but the rapid global expansion has resulted in the accumulation of over 2,000 known mutations within the viral genome. The significance of these mutations to pathogenesis and transmission is not yet clear and require further study. However, recent mutations in strains transmitted from mink have shown changes in recognition by S protein-specific antibodies, highlighting the risk that viral mutation may lead to immune evasion and therefore reduction in protective efficacy of first generation vaccines based on the sequences of early viral isolates. Furthermore, emerging data from the UK suggests a new variant that potentially can transmit more readily but it is unclear at this point if this is associated with any change in virulence or significant antigenicity changes. Typically, as viruses evolve to more readily transmit between their hosts this is more often associated with a decrease in virulence.

2.4 Virological Correlates of Infection

Figure 1 shows SARS-CoV-2 viral load in the upper respiratory tracts of naturally infected symptomatic patients by qPCR and culture. The highest viral loads, are observed in the first 14 days after onset of symptoms. With respect to recovery from infection, many people have viral RNA (detected by swab PCR test on a nasal sample) but do not shed live virus that can be grown in a lab or infect others. The loss of viral infectivity happens at around day 8-9 (van Kampen et al., 2020), when the viral load (detected by PCR) is in decline and antibody is starting to appear. A qPCR Ct value >35 has been presented to the New and Emerging Respiratory Virus Threats (NERVTAG) committee as associated with lack of infectivity (Wendy Barclay and Peter Openshaw, Imperial College London, personal communication).

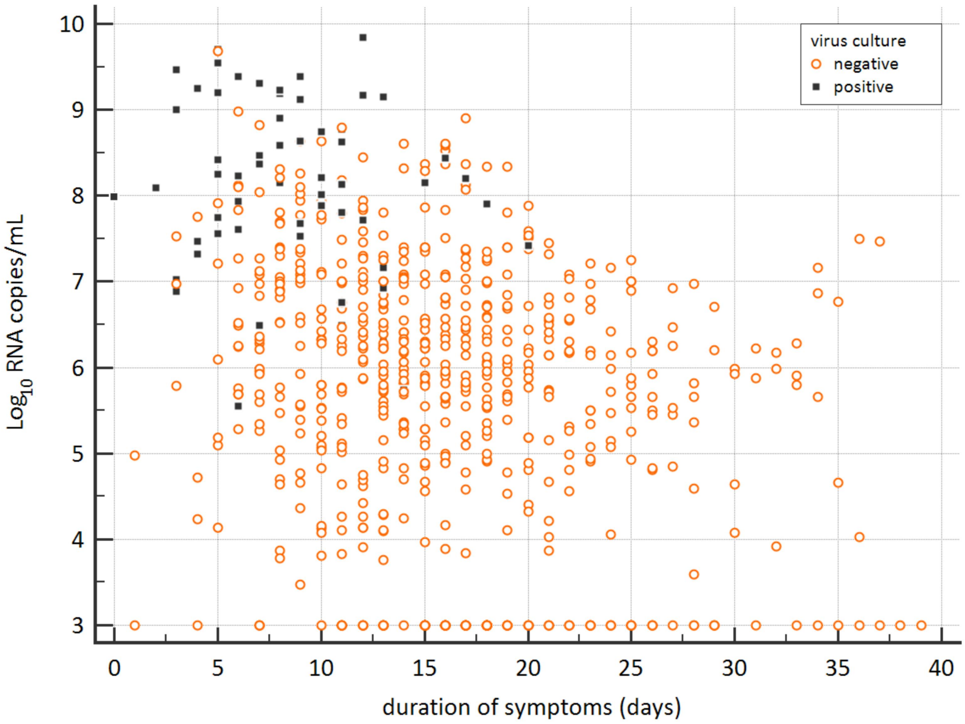

Figure 1 SARS-CoV-2 viral load in naturally infected patients by qPCR and culture.

The Scientific Advisory Group for Emergencies (SAGE) has been advised by NERVTAG that it is unlikely that people who are recovering from SARS-CoV-2 infection and have developed antibody in nasal secretions, blood or serum are still infectious.

2.5 Immunology of COVID-19

Following SARS-CoV-2 infection, an immunological response is demonstrated, but despite extensive studies, there is still limited evidence about the type and level of immune response that protects people from re-infection. However, with the efficacy of first-generation vaccines and the number of documented re-infections remaining low despite high incidence rates in some countries, it is clear that immunity after vaccination or natural infection can provide resistance to re-infection, although the duration of protection remains unknown.

Antibody responses

The frequency with which people infected with SARS-CoV-2 make an antibody response to the major antigens (including the spike protein S and the nucleoprotein N) is dependent on the test used. Previous reports that a high proportion of those with mild disease fail to show a response were based on earlier versions of the antibody test, that have now been superseded.

Recent evidence from animal models showed that antibodies against spike protein administered passively protect against subsequent challenge with SARS CoV2 virus (Rogers et al., 2020). This is especially true when the antibodies are neutralizing *in vitro*. Such antibody protects cells

from infection by blocking the interaction between the spike receptor binding domain and the ACE2 receptor. However, other antibodies that bind to spike protein but did not neutralize *in vitro* may fail to protect against infection. The success of the passively transferred antibodies in animal models suggests that antibody prophylaxis (and possibly, therapy) either with convalescent plasma or with cloned antibodies could be protective in humans. The optimal therapy might be with polyclonal antibody, since single antibodies might drive virus escape.

Most people make a polyclonal response after virus infection that targets many different proteins and epitopes within them (Brouwer et al., 2020). Thus, it might be expected that most people will make neutralizing antibodies that are protective against reinfection. However, the specific protective (neutralizing) antibodies are not measured in all the different antibody tests that have been developed so far. For example, some tests measure antibodies against N protein. Some of the latest serological tests do measure antibodies that target the receptor binding domain specifically, or even measure the antibody's ability to disrupt the interaction between spike and ACE2 receptor. A positive, high titre result in these tests would give some reassurance that an individual was 'immune' and unlikely to be susceptible to reinfection. This is supported by efficacy data from first-generation SARS-CoV-2 vaccines, which primarily have sought to stimulate high antibody levels.

However, it remains uncertain what titre of neutralizing antibody is required for immunity; it is also uncertain how long antibody persists and how effectively it is boosted by re-exposure or vaccination. In addition, antibody in the blood may not be reflected by protection against lung disease (although this seems probable) or against infection of the nose and pharynx (less likely). Early results from non-human primate vaccine and challenge studies performed on the ChAdOx Oxford vaccine showed modest levels of antibody and some protection against disease but not against infection in the upper airway (van Doremalen et al., 2020). However, the studies were performed very early after vaccination and with a high dose of challenge virus delivered both to the upper and lower airway. The design of the study was inherently likely to give a less than protective signal.

T cell responses

T helper (CD4+) cells are essential to durable B cell (antibody-mediated) immunity. The T cells respond to antigen presenting cells that present fragments of virus (T cell epitopes) in conjunction with MHC class II. T cell epitopes have recently been described for SARS-CoV-2 (Grifoni et al., 2020) and are located in spike, nucleoprotein and M proteins as well as in other non-structural viral proteins. A substantial number (>40%) of people unexposed to SARS-CoV-2 were found to have CD4+ T cell responses that were likely elicited by infection with previous seasonal coronavirus infections. The presence of CD4+ T cell epitopes in spike protein suggests that vaccines that consist only of spike will elicit an immune response, but it will differ from that after natural infection. Infection will normally generate durable CD4 T cell responses, but purified antigens may not, although these cells have been detected after ChAdOx1 nCoV-19 vaccination.

CD8+ T cells responses have been detected in people recovering from SARS-CoV-2 infections. Some reacted to spike proteins, but several other proteins were more dominant. In another study CD8+ T cells were present in lungs of people with mild but not with severe disease suggesting they may modify disease outcome (Bost et al., 2020). Purified spike protein vaccines are unlikely to elicit CD8+ responses unless formulated with an adjuvant that promotes such responses, and the value of CD8 responses is undetermined.

Harmful Immune responses

There has been some concern around antibody dependent enhancement (ADE) of disease and other mechanisms by which re-infection with SARS-CoV-2 in the presence of suboptimal pre-existing immunity may lead to more severe disease, for example due to immunopathology (Iwasaki and Yang, 2020). While there have only been a few cases of documented re-infection reported, one recent case report described a young healthy adult whose first episode of COVID-19 was mild but who was re-infected with a variant strain with more severe disease requiring hospitalisation (Tillett et al., 2020). There is therefore a theoretical risk that previous immune responses (by infection or vaccination) may contribute to more severe disease on re-infection. However, there is still no robust evidence of this for SARS-CoV-2. In one paper using passive transfer of antibody to hamsters, there was a small signal of increased weight loss over control for the groups receiving low dose antibody (Rogers et al., 2020). Numbers were too small to conclude this was ADE and no mechanistic studies were performed. In respiratory syncytial virus (RSV) disease (which shows many similarities to COVID-19 in terms of mechanisms of disease and immunity), T cells may contribute to enhanced disease in the absence of neutralising antibody.

The key immunological questions that might be resolved by human challenge therefore include:

- The protection afforded by new vaccines
- The role of antibody vs. cellular immunity in immune protection and disease
- The immune biomarkers that predict variations in symptoms
- The durability of immunity in those previously infected with SARS-CoV-2
- The effects of passive antibody therapy on viral infection and recovery

2.6 Clinical outcomes of SARS-CoV-2 infection

This protocol has been designed to minimise, as much as possible, risk to participants and their contacts, whilst providing a robust platform for vaccine efficacy evaluation. For the purpose of protocol design and informed consent, the degree of risk can be semi-quantified using data from observational studies of natural infection, which have also identified factors associated with risk of severe disease such as age, co-morbidities, gender and ethnicity. Animal models provide information correlating severity of pathology with route and dose of inoculation. In addition, there is emerging information concerning the efficacy of antiviral and immunomodulating therapy, which has the potential to attenuate risk to participants.

Much of the information that informs the general public of COVID-19, and indeed the scientific community, is derived from hospital-treated cases, that are, by definition, at the severe end of

the spectrum. In conducting a dose ranging study commencing with an inoculum at the lowest measurable dose, investigators aim to achieve a model in which volunteers experience an infection that results in no symptoms, or symptoms no more severe than the common mild response of healthy people of the same age within the general population.

Age as a risk factor for severe COVID-19

Age has been a major factor in severe outcome of COVID-19 in all series published so far (Spiegelhalter, 2020). In one large meta-analysis by the Imperial group of data from China, an estimated 20% of hospital-treated infections were severe with an overall infection-mortality rate of approximately 2% (Salje et al., 2020). Using this huge dataset, it was estimated that the infection-mortality rate (95% confidence interval) in 20–29-year-olds was 0·0309% (0·0138–0·0923), and in 30-39 year olds was 0·0844% (0·0408–0·185). In contrast, the estimated infection-mortality rate in the over-60s was 3·28% (1·82–6·18).

Modelling from France estimated that 0.5% of 20-29 year olds who were infected during the first pandemic wave (including those with co-morbidities) were admitted to hospital, using denominator data from passive surveillance and proportions of asymptomatically infected individuals from the *Diamond Princess* cruise ship to estimate the total number of infections over the same period (Sakurai et al., 2020). These are likely to have been over-estimates, as more recent analysis of severe outcomes from several European countries using denominators estimated by seroprevalence data showed the following in young adults <30 years old (Kuiper et al., 2020).

- Risk of death following infection: 1.2-6.1 per 100,000 (0.0012-0.0061%)
- Risk of ICU following infection: 0.9-4.5 in 10,000 (0.009-0.045%)
- Risk of hospitalization following infection: 0.8-3.9 per 1,000 (0.08-0.39%)

Even these are likely to be over-estimates for a healthy young adult group, as the figures do not take risk factors or co-morbidities into account. No published data exist specifically describing the risk of hospitalization in the UK of individuals in the 18-30 year old age group once they are already infected. As of 6th November 2020, 95 adults aged 18-30 have died due to COVID-19, the majority of whom had co-morbidities or other risk factors (Ben Humberstone, Office of National Statistics [ONS], personal communication,

https://www.england.nhs.uk/statistics/statistical-work-areas/covid-19-daily-deaths/).

Data from the ONS from the 16 weeks between 7th March and 26th June 2020 (Williamson et al., 2020) covering the peak of the first pandemic wave show an estimated absolute risk of death in those aged 15-24 years of 0.5 in 100,000 (0.0005%) and those aged 25-34 of 1.6 in 100,000 (0.0016%). Additionally, the QCOVID living risk prediction algorithm (Harrison, 2020) provides an absolute risk of COVID-associated hospitalization in a White British 30 year old woman with no risk factors as 1 in 5076 (0.0197%).

Extrapolating study findings from young adults to high risk groups such as older people with co-morbidities is not always possible. However, young adults with mature intact immune systems are the benchmark for optimal immune responses. They are therefore the best group in which to

identify correlates of protection against which vaccine-induced responses can be compared. So far, phase III studies have shown no major differences between young and older vaccinees, which also reassures about extrapolating in this context. Furthermore, a vaccine candidate tested by infection challenge of young adults that showed no effect on infection rate or viral shedding can reasonably be understood to have little protective efficacy in those with young healthy immune systems, and therefore would be even less likely to show efficacy in more high-risk populations with impaired immune responses. Host factors such as age are also less likely to impact the efficacy of antiviral drugs and monoclonal antibodies, so extrapolation is more feasible for these types of studies. Finally, with widespread vaccination in the near future, the majority of high-risk individuals will have been vaccinated and almost all efficacy studies, including head-to-head comparisons of drugs and vaccines, will need to take place in younger volunteers. While young adults with no known underlying conditions may still develop unexpectedly severe outcomes following virus or drug administration, these are likely to be rare and can be further mitigated in an early phase clinical trial setting.

Detailed analyses of the existing phase III vaccine trials are not yet available, so although preliminary announcements have suggested no difference in efficacy in older adults and other high-risk groups this has yet to be fully evaluated. In the published Pfizer data, analysis was based on only 170 infections, with only 20 cases in those aged >65 and 5 cases in those aged >75, who are likely to respond least well to vaccination (Polack et al., 2020). Similar issues with statistical power apply to subgroups with co-morbidities or immunosuppression, so we would argue that the efficacy of these first-generation vaccines remains uncertain and there remains a strong rationale to continue developing vaccines to improve protection in those who have difficulty mounting good immune responses.

Ethnicity as a risk factor for severe COVID-19

Initial analysis by the Office for National Statistics (ONS) (https://www.ons.gov.uk/peoplepopulationandcommunity/birthsdeathsandmarriages/deaths/articles/updatingethniccontrastsindeathsinvolvingthecoronaviruscovid19englandandwales/deathsoccurring2marchto28july2020) showed that the rate of death involving the coronavirus (COVID-19) among almost all ethnic groups has been significantly higher than that of those of White British ethnicity. Based on an initial statistical model adjusting for age, males of Black African ethnic background were 3.8 times more likely to die from a COVID-19-related death and females of Black African ethnic background were 2.9 times more likely than males and females who self-identified as White British People. Bangladeshi, Pakistani, Indian and Mixed ethnicities also had statistically significantly raised risk of death involving COVID-19 compared with those of White ethnicity. However, much of this increased risk disappeared after taking into account geography, socio-economic characteristics and health measures such as pre-existing conditions, with Black African males having a 2.5 times higher corrected risk, Black African females having a 2.1 times greater corrected. For other ethnic minority groups the increased rate of COVID-19 mortality was also reduced following correction and the ONS concluded that the majority of the difference between ethnic groups in COVID-19 mortality was a result of socio-economic disadvantage and other circumstances such as high rates of employment in essential services including front line health care.

These data have been partially supported by findings from other studies. The OpenSAFELY study showed that after adjustment for other factors, Black and South Asian people were at higher risk with hazard ratios of 1.48 and 1.45 respectively (He et al., 2020). However, the ISARIC-4C study showed an increased risk only in South Asian people after correction for other factors (Tenforde, 2020). Some of this remaining increased risk may be due to unmeasured social and environment factors, although it is not possible to exclude biological risk factors at this stage. Thus, while there is an increased risk of poor outcomes following COVID-19 in people of some ethnic backgrounds, most is due to exposures, socioeconomic and health inequality factors, the data for which are incomplete. Furthermore, while the relative risk of death may be higher even when certain factors have been taken into account, in young adults this still represents a very low absolute risk of severe outcomes. This is reflected in the QCOVID risk model, where risk of severe outcomes in young adults remains extremely low irrespective of ethnicity (Harrison, 2020).

Consequences of natural infection

Natural infection by SARS-CoV-2 mostly results in no or minor symptoms. In the careful observation of passengers and crew in quarantine on board the cruise ship *Diamond Princess*, 712 of a total of 3711 persons were found to be infected with SARS-CoV-2, and 410 (58%) of those infected were asymptomatic at the time of testing. Amongst the latter asymptomatic cases, a small fraction - 12% - subsequently developed symptoms (Berlin et al., 2020). Where infection results in symptoms, international published epidemiological surveys indicate a median incubation period of 5 days. People infected with SARS-CoV-2 are most infectious (i.e. exhibit significant viral shedding) from 2 days before, until 7 days after symptom onset (Docherty et al., 2020).

Evidence from observational studies of infected individuals and non-human primates have shown that changes occur in the lungs even sometimes during asymptomatic (Long et al., 2020). These are generally seen as ground-glass shadows on imaging that may be temporary or persistent. They can be early (around 5 days post-infection) or late (around 10 days) and can progress to more severe lung inflammation characterised by widespread changes in both lungs.

A wide range of symptoms can occur from disease onset. In a detailed, multi-site CDC study of 274 people in the United States with symptoms, who were tested as outpatients and found to be positive by RT-PCR and then interviewed in the period 14-21 days after the initial swab, the complaints experienced in descending order of frequency were fatigue, cough, headache, body ache, fever, chills, loss of taste, loss of smell, diarrhoea, congestion, dyspnoea, nausea, sore throat, chest pain, abdominal pain, confusion and vomiting. Among respondents aged 18–34 years with no chronic medical condition, 19% (9 of 48) reported not having returned to their usual state of health. Age ≥50 versus 18–34 years (adjusted odds ratio [aOR] = 2.29) and reporting 3 or more versus no chronic medical conditions (aOR = 2.29) were associated with failure to regain usual health. Obesity (body mass index ≥30 kg per m^2) (aOR 2.31) and a psychiatric condition (aOR 2.32) also were associated with failure to recover completely after adjusting for age, sex, and race/ethnicity.

Consequences of development of disease

In a minority of higher-risk individuals, initial symptoms of natural infection are followed by a dramatic decline in clinical state, characterized by worsening dry cough, severe dyspnoea and profound malaise. This is caused primarily by uncontrolled viral replication and dissemination to the lower respiratory tract followed by onset of a diffuse maladaptive lung inflammation causing failure of gas exchange resulting in low arterial oxygen saturation. This, together with generalised sepsis (including coagulation disorders and cardiac complications) can lead to multi-organ failure. Severe COVID-19 may also lead to acute cardiac, kidney, and liver injury, in addition to cardiac arrhythmias, rhabdomyolysis, coagulopathy, and septic shock. In a small number of children and teenagers, a condition (Paediatric Inflammatory Multisystem Syndrome temporally associated with COVID-19 or PIMS-TS) has also been reported that leads to more severe disease (Jiang et al., 2020; Whittaker et al., 2020). It is unclear whether this syndrome occurs in adults.

The UK ISARIC study investigated 20,133 people with COVID-19 admitted to hospitals whose median age was 73 years (interquartile range 58-82), the median duration of symptoms before admission was 4 days and the median duration of hospital stay was 7 days (Docherty et al., 2020). Amongst this cohort, 23% had no documented reported comorbidity. The commonest comorbidities of the remainder were chronic cardiac disease (31%), diabetes (21%), chronic pulmonary disease (18%), asthma (14%) and Chronic Kidney disease (16%). Increased age and comorbidities including obesity were associated with a higher probability of mortality. The investigators reported 3 major and distinct clusters of symptoms: (i) respiratory (cough, sputum, sore throat, runny nose, ear pain, wheeze, and chest pain) – the commonest cluster; (ii) systemic (myalgia, joint pain and fatigue); and (iii) enteric (abdominal pain, vomiting and diarrhoea). Across the entire cohort, 41% of patients were discharged alive, 32% died and 41% continued to receive care at date of reporting. 17% required admission to High Dependency or Intensive Care Units; of these, 17% were discharged alive, 37% died and 46% continued to receive care at the reporting date. Of those receiving mechanical ventilation, 20% were discharged alive, 53% died and 27% remained in hospital. Overall, younger age, female sex, and lack of co-morbidities or obesity were associated with lower mortality in hospital. However, individuals aged 18-30 made up only a small proportion of cases (with no male preponderance) (Docherty et al., 2020).

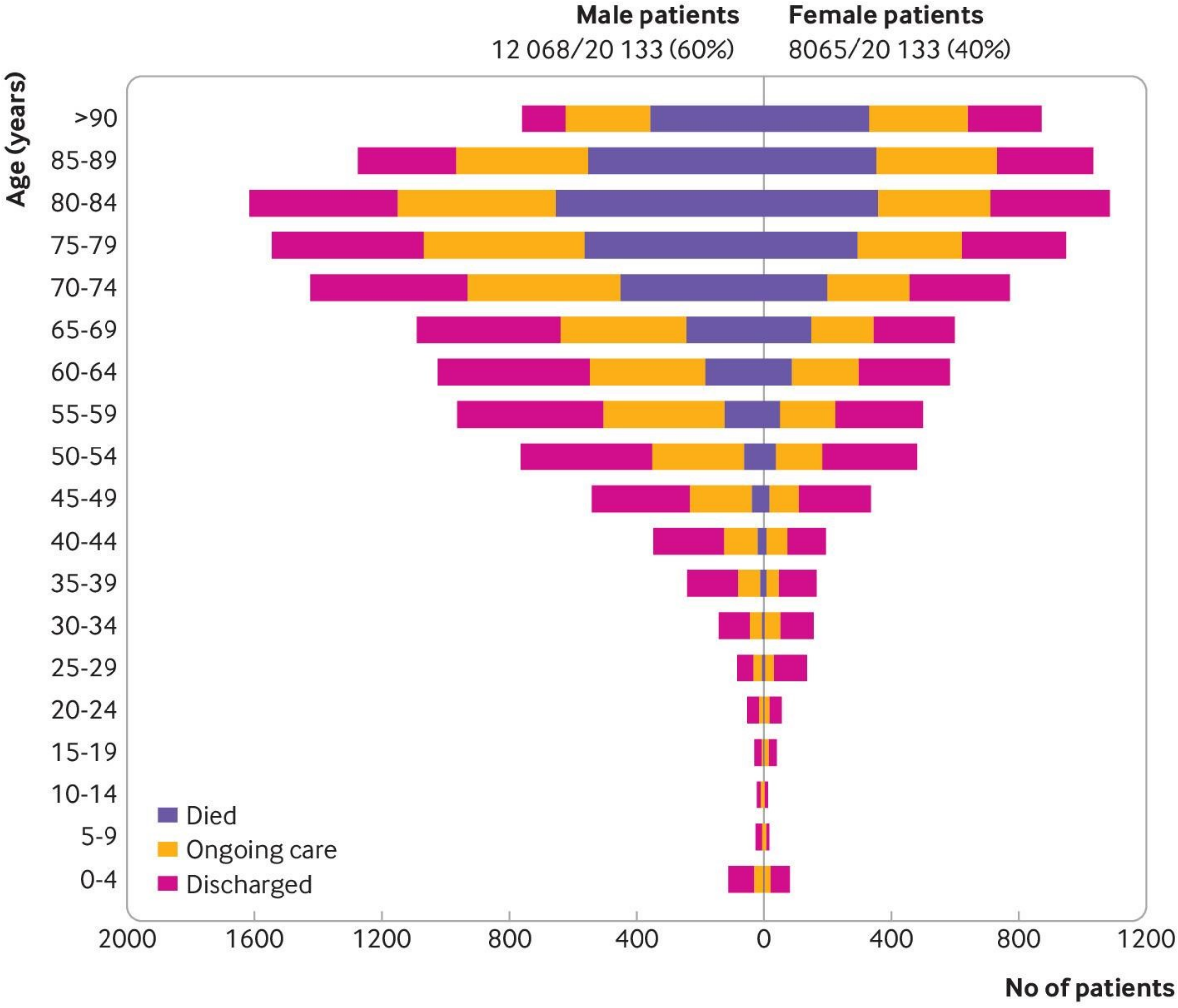

Figure 2 Outcomes of 20,133 hospitalised COVID-19 patients by age

Together, these data suggest that SARS-CoV-2 infection in healthy young adults with no co-morbidities rarely causes more than mild self-limiting symptoms.

Treatment of Severe COVID-19

Patients with severe COVID-19 often require oxygen and ventilatory support, together with correction of physiological dysfunction usually in intensive care units. The most severely ill usually require mechanical ventilation, plus one or more of haemodynamic support, correction of coagulopathy, treatment of heart failure and correction of acute renal failure. Standard of care of severely ill people now includes dexamethasone, Remdesivir and Tocilizumab. In patients hospitalized with COVID-19, the use of dexamethasone resulted in lower 28-day mortality among those who were receiving either invasive mechanical ventilation or oxygen alone at randomization but not among those receiving no respiratory support (Horby et al., 2020). While the efficacy differs between studies, treatment with the anti-viral drug Remdesivir 200mg IV daily has been shown to be superior to placebo in shortening the time to recovery in adults

hospitalized with COVID-19 and evidence of lower respiratory tract infection (Beigel et al., 2020) In contrast, the recent SOLIDARITY trial (WHO_Solidarity_Trial_Consortium, 2020) suggested no overall improvement in mortality or clinical outcome in hospitalized patients, but this non-placebo controlled international multi-centre study enrolled a wide range of patients at various stages including late disease, which cannot be extrapolated to early treatment of mild infection (see 0 Pre-clinical and clinical experience with Remdesivir for COVID-19).

Potential long-term complications of COVID-19

Currently, it is still too early to assess the long-term complications of COVID-19. However, it is possible that severe ARDS may be associated with long-term pulmonary pathology including fibrosis. In the case of the SARS outbreak of 2003, a 2-year study of a selected population of SARS survivors, showed significant impairment of DLCO, exercise capacity and health status had persisted (Ngai et al., 2010). In the wake of the SARS epidemic, some survivors experienced chronic fatigue syndrome for several years after illness (Lam et al., 2009). Currently, large cohort studies (such as the UK sponsored PHOSP-Covid) have been set up to collect this information. However, to date, there has been no evidence that long-term sequelae are significantly associated with asymptomatic or mild SARS-CoV-2 infection. “Long COVID” is currently being widely discussed (Maxwell, 2020) and refers to a post-infective state that has been experienced by COVID-19 patients including health care workers, characterised by fatigue and lassitude, extending for weeks and even months. The incidence and nature of this illness is currently being investigated by several groups internationally (Nabavi, 2020).

Data from the COVID Symptom Study (led by Tim Spector and Claire Steves at King’s College London) is using self-reported symptom data from a mobile phone app to analyse the frequency and duration of symptoms related to COVID-19. In that dataset, preliminary data were obtained of 629 individuals in the 18-30 year old age group with PCR-confirmed SARS-CoV-2 infection and who were non-smokers, had a BMI<25, had no co-morbidities and consistently logged into the app (data extracted September 2020, personal communication, Claire Steves, King’s College London). In this cohort, 78% were women but the frequencies and duration of symptoms were similar between men and women (Table 3 & Table 4). The most frequent symptoms were fatigue (78%), headache (74%), loss of smell (61%), sore throat (59%) and cough (48%) as shown in Table 3. On average, these symptoms lasted no more than 5 days although some rare individuals experienced loss of smell and fatigue for up to 4 months before resolution (Table 4, Figure 3). Nevertheless, fatigue had resolved in 75% of individuals after 11 days or fewer and 90% of individuals after 19 days or fewer, and loss of smell in 75% after 9 days or fewer and 90% after 14 days or fewer (Figure 2). Further analysis of the overall dataset has allowed the development of a risk prediction system for “long COVID”, which has shown relatively lower risk in younger individuals with <5 symptoms (Sudre et al., 2020).

Table 3 Frequency of COVID-19 related symptoms in healthy 18-30 year olds with PCR-confirmed infection

Frequency	Overall	Male	Female
Fatigue	0.783784	0.791045	0.780933
Headache	0.739269	0.671642	0.756592
Loss of smell	0.605723	0.544776	0.62069
Sore Throat	0.586645	0.514925	0.604462
Persistent cough	0.481717	0.492537	0.476673
Shortness of breath	0.36725	0.343284	0.371197
Fever	0.36407	0.425373	0.344828
Chest pain	0.357711	0.291045	0.375254
Skipping meals	0.349762	0.298507	0.363083
Unusual muscle pains	0.343402	0.358209	0.338742
Hoarse voice	0.298887	0.246269	0.312373
Diarrhoea	0.233704	0.186567	0.245436
Dizziness	0.197138	0.156716	0.208925
Abdominal pain	0.18124	0.156716	0.184584
Eye soreness	0.151033	0.141791	0.15213
Confusion, disorientation or drowsiness	0.138315	0.164179	0.127789
Rashes	0.028617	0.014925	0.032454
Blisters	0.009539	0.007463	0.010142

Table 4 Duration of COVID-19 related symptoms in healthy 18-30 year olds with PCR-confirmed infection.

Duration (days)					
**Symptom**	**Median**	**Min**	**Max**	**75th centile**	**90th centile**
**Loss of smell**	5	1	119	9	14
**Fatigue**	5	1	102.5	11	19
**Persistent cough**	4	1	46	9	14.8
**Headache**	3.5	1	72	8	15
**Shortness of breath**	3	1	92	9	17
**Chest pain**	3	1	92	7	17
**Skipping meals**	3	1	66	6	10
**Sore Throat**	3	1	52.5	7	12.2
**Hoarse voice**	2.5	1	46	6	11
**Confusion, disorientation or drowsiness**	2.5	1	34	6	11.4
**Dizziness**	2.5	1	32.5	7	18
**Rashes**	2.25	1	9.5	4.75	6.9
**Unusual muscle pains**	2	1	92	5	9.5
**Abdominal pain**	2	1	66	5.75	12
**Fever**	2	1	46	4	8
**Diarrhoea**	2	1	32.5	4	9
**Eye soreness**	2	1	29	4	9.2
**Blisters**	1.25	1	9	1.75	5.5

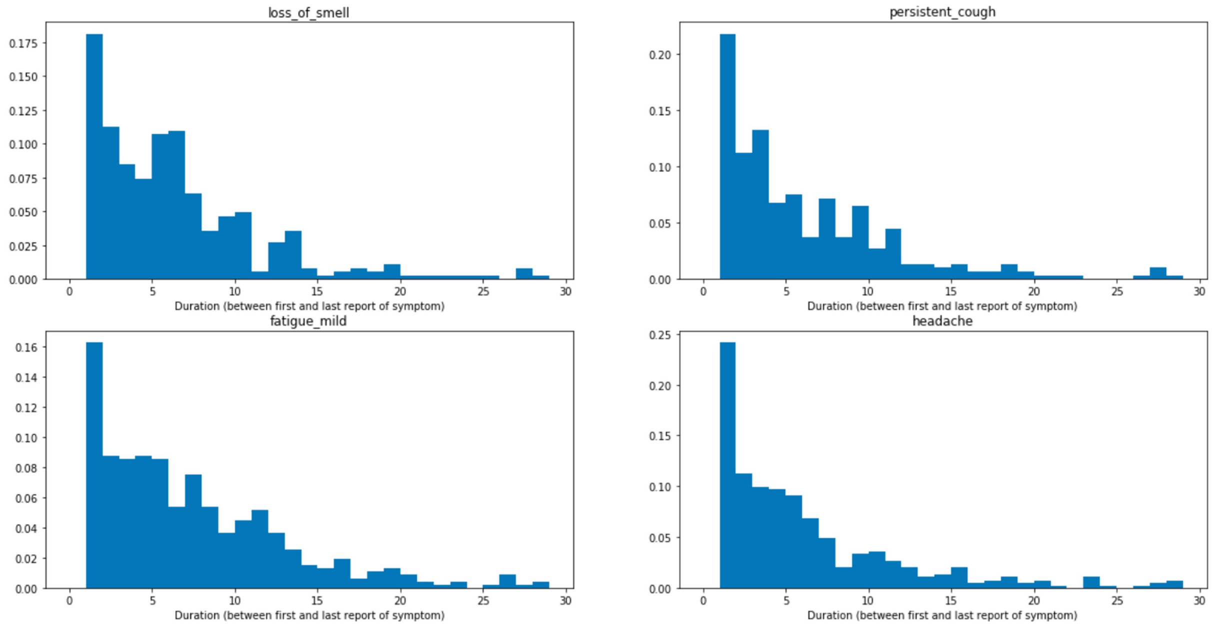

Figure 3 Frequency of individuals with the most common COVID-19 associated symptoms by duration.

From these data and considering the intranasal inoculation of SARS-CoV-2 in this study, loss and alteration of smell is a potential specific risk, even in healthy young adults and may persist in rare cases for weeks to months. Objective testing has shown that anosmia may be even more common than when assessed by self-report and may be the only symptom, particularly in young adults. In addition, parosmia (alteration in the sense of smell that can severely impair appetite due to familiar foods triggering a foul smell) has previously been reported in a large proportion of those with post-viral loss of smell. It is now also being reported during the COVID-19 pandemic and may persist for longer than anosmia. While anosmia is most common in those aged 40-45, parosmia may occur with similar frequency across the age ranges. Some data exist that suggests the usefulness of treatments including steroid rinses, topical vitamin A and omega-3 supplements that may reduce these symptoms or speed recovery. A placebo-controlled randomized controlled trial accepted for publication has shown that oral and intranasal steroids given at 4 week in those with persistent anosmia achieves better recovery rates (Claire Hopkins, ENT consultant, Guy's and St Thomas' Hospital, personal communication).

2.7 Mitigating Risk in Experimental Human SARS-CoV-2 Infection

Taken together, these data suggest that to minimise the likelihood of injury to participants, the inclusion criteria should specify young age (18-30 years) and exclusion criteria should consider currently known risk factors such as obesity and any comorbidity detected on screening. Additionally, a risk prediction tool such as QCOVID should be used for personalised risk assessment of volunteers using a pre-specified absolute risk threshold until the challenge

infection has been shown to be safe and very well tolerated. Other ways to reduce risk as much as possible include (i) use of a low infecting dose, (ii) intranasal deposition of inoculum (avoiding direct instillation to the lung), (iii) early recognition of features associated with progression to severe disease, including viral hyper-replication and lower airway inflammation and (iv) intervention treatment of infected participants in the form of pre-emptive or rescue therapy, when indicated.

Role of inoculum density in severity of infection

It is intuitive that the concentration of virus to which a participant is exposed is likely to determine the chances of successful infection, but also of severity of the inflammatory response and, potentially, the disease. We are not aware of relevant infecting dose experiments with animal models of COVID-19, but animal models of other viral infections show that variation in the infecting dose determines the severity of the disease. A dose-response has been shown in mouse models with several strains of SARS-CoV-1. The infectivity varies between different strains of the virus, which modifies the shape of the dose-response curve, but nevertheless consistent dose-response relations are observed with the severity of the infection (Roberts et al., 2008). Human influenza infection challenge studies have also demonstrated higher infection rates related to dose (Watson et al., 2015). In this study, we will therefore adopt a strategy of infecting participants with the lowest dose of SARS-CoV-2 that can be consistently administered, and then conduct a series of dose increment cycles until we identify the lowest dose which achieves the objective of viral replication in the upper respiratory tract of ≥50-70% of participants.

Significance of high viral loads within the upper airway

Longitudinal studies have not established any correlation between SARS-COV-2 viral load in the respiratory tract and disease severity in younger patients due to the high frequency of asymptomatic disease. In symptomatic hospitalised patients, the highest levels of pharyngeal virus shedding occur during the first week of symptoms, with a peak on day 4 (Wölfel et al., 2020). Transient peak viral loads of 7×10^8 RNA copies/swab have been reported in upper and lower respiratory tract samples but decline rapidly after day 5. In hospitalised patients with mild-severe disease, the median duration of infectious virus shedding is 8 days (van Kampen et al., 2020). Shedding of viral RNA from respiratory tract samples generally outlast the end of symptoms. Viral loads above 10^7 RNA copies/mL samples have been independently associated with detection of infectious SARS-CoV-2. Patients with $<10^6$ viral RNA copies/mL are highly unlikely to be infectious (van Kampen et al., 2020; Wölfel et al., 2020).

Personalised risk assessment using the QCOVID tool

The safety of volunteers enrolled in these studies is of utmost concern and ethnicity has been discussed widely as a potential risk factor for hospitalisation and death with COVID-19. The role of ethnicity as an exclusion factor was therefore discussed extensively in the protocol development group, specifically that of Black, Asian and minority ethnicities (BAME). Analysis by the Office of National Statistics and the OpenSAFELY and ISARIC 4C studies of hospitalized patients have all concluded that the majority of the increased risk seen (particularly in Black and South Asian groups) was related to socioeconomic factors including greater exposure due to

disproportionately being in front-line jobs. However, there remained some element of increased risk in BAME individuals that was still unexplained, with up to ~2.5 times increased risk of severe outcomes in Black African men, for example. Our public and participant inclusion and engagement as well as reviews by experts in BAME health (including Kevin Fenton, PHE, and Kamlesh Khunti, Centre for BME Health) highlighted two opposing views: (1) that inclusion and diversity should be maximized, and (2) that BAME people should not be subjected to any increased risk before those with no documented risk had been through the study procedures. To take these views into account and since this increase in risk was not identical across ethnicities, it was felt that a personalised risk assessment would be a better way to balance inclusivity with safety. Furthermore, a recruitment approach should be responsive to the most up-to-date data. The QCOVID risk scoring tool (qcovid.org) published by a consortium led by Julia Hippisley-Cox (University of Oxford) is an independent, validated risk assessment algorithm that integrates age, sex, ethnicity, geography, body mass index and co-morbidities to provide an individualised estimate of absolute mortality and hospitalisation risk. This provides an objective absolute risk of death and hospitalization based on the best available UK epidemiologic data. The tool is CE marked and will be recalibrated with up-to-date data every 3-6 months.

Table 5 Representative risk assessments using QCOVID

Absolute risk of death	Absolute risk of hospitalisation	Age	Sex	Ethnicity	BMI
1 in 250,000 (0.0004%)	1 in 4902 (0.020%)	30	F	White British	23.5
1 in 250,000 (0.0004%)	1 in 7143 (0.014%)	30	M	White British	23.5
1 in 250,000 (0.0004%)	1 in 8475 (0.012%)	22	M	Black African	23.5
1 in 250,000 (0.0004%)	1 in 5319 (0.019%)	26	M	Indian	23.5
1 in 250,000 (0.0004%)	1 in 9901 (0.010%)	24	M	Chinese	23.5
1 in 250,000 (0.0004%)	1 in 4587 (0.022%)	27	F	Chinese	23.5
1 in 250,000 (0.0004%)	1 in 4329 (0.023%)	30	F	White British	27.7
1 in 250,000 (0.0004%)	1 in 6211 (0.016%)	30	M	White British	27.7
1 in 250,000 (0.0004%)	1 in 7143 (0.014%)	22	M	Black African	27.7
1 in 250,000 (0.0004%)	1 in 3984 (0.025%)	27	M	Indian	27.7
1 in 250,000 (0.0004%)	1 in 8621 (0.012%)	24	M	Chinese	27.7
1 in 250,000 (0.0004%)	1 in 4000 (0.025%)	27	F	Chinese	27.7
1 in 200,000 (0.0003%)	1 in 2475 (0.04%)	22	F	Black African	23.5
1 in 200,000 (0.0003%)	1 in 2959 (0.034%)	23	F	Indian	23.5
1 in 200,000 (0.0003%)	1 in 4329 (0.023%)	30	F	Chinese	23.5
1 in 166,667 (0.0006%)	1 in 3333 (0.03%)	30	M	Indian	23.5
1 in 125,000 (0.0008%)	1 in 2632 (0.038%)	30	F	Indian	23.5
1 in 111,111 (0.0009%)	1 in 2169 (0.046%)	30	F	Black African	23.5
1 in 100,000 (0.001%)	1 in 4739 (0.021%)	30	M	Chinese	23.5
1 in 83,333 (0.0012%)	1 in 2755 (0.036%)	30	M	Black African	23.5

2.8 Pre-clinical and clinical experience with Remdesivir for COVID-19

In a macaque model of COVID-19, treatment with Remdesivir 12 hours after respiratory tract inoculation of SARS-CoV-2 reduced subsequent signs of respiratory disease, radiologically defined pulmonary infiltrates and virus titres in bronchoalveolar lavages 2 hours after the first treatment administration. However, virus shedding from the upper respiratory tract was not reduced by Remdesivir treatment (Williamson et al., 2020). In a clinical study of 1063 patients with COVID-19, the data and safety monitoring board recommended early unblinding because of shortened time to recovery in the Remdesivir group (Beigel et al., 2020). Results from 1062 patients (541 assigned to Remdesivir and 521 to placebo) with data available after randomization indicated that those who received Remdesivir had a significantly reduced median recovery time of 10 days compared with 15 days in those who received placebo (Beigel et al., 2020). Estimates of mortality by 15 days were 6.7% with Remdesivir and 11.9% with placebo. Serious adverse events were no more frequent in the Remdesivir group compared with placebo. In a further study, treatment with Remdesivir 200mg IV daily for 5 days achieved similar clinical outcomes to 10 days (Goldman et al., 2020).

However, in a smaller Chinese study of Remdesivir 237 patients with proven COVID and hypoxaemia were enrolled and randomly assigned to a treatment group (158 to Remdesivir and 79 to placebo), Remdesivir was not associated with a difference in time to clinical improvement. Nevertheless, although not statistically significant, patients receiving Remdesivir had a numerically faster time to clinical improvement than those receiving placebo among patients with symptom duration of 10 days or less (hazard ratio 1·52 [0·95-2·43]). Adverse events were reported in 102 (66%) of 155 Remdesivir recipients versus 50 (64%) of 78 placebo recipients. Remdesivir was stopped early because of adverse events in 18 (12%) patients versus four (5%) patients who stopped placebo early (Wang et al., 2020). Recently, data from the non-placebo controlled multi-centre WHO Solidarity trial also showed no difference in mortality or length of hospitalization in patients treated with Remdesivir compared to placebo (Pan et al., 2020). However, subgroup and meta-analysis conducted by the investigators did show statistically significant reductions in hospital stay in younger patients with less severe disease and oxygen requirement (Shrestha et al., 2020). This supported the randomized placebo-controlled ACTT-1 trial data that showed significant reduction in hospital duration in those individuals treated with earlier and less severe disease. Furthermore, data from immunosuppressed individuals have shown viral suppression and improvement in clinical indices correlating directly with Remdesivir treatment (Buckland et al., 2020; Helleberg et al., 2020). These data clearly indicate physiological and antiviral effects of Remdesivir *in vivo*, despite the clinical benefit for most hospitalized patients remaining in question (due to unavoidably delayed treatment in the clinical setting).

In the hospitalized patients described in clinical trials to date, it is not possible to administer Remdesivir at the early pre-symptomatic timepoints proposed here. Antivirals against influenza have invariably shown greater efficacy when given early in the disease course and a number of antivirals against influenza and RSV have shown greater efficacy (and in some cases only shown efficacy) in the controlled human challenge model and not in clinical practice (DeVincenzo et al., 2015; Hayden et al., 1999). This along with the non-human primate data

imply that the same may be true for Remdesivir, where early pre-emptive treatment while viral load is low is likely to have greater efficacy than later treatment in the context of uncontrolled viral replication and inflammatory responses that can no longer be ameliorated by reduction in viral load.

The UK's Medicines and Healthcare Products Regulatory Agency (MHRA) granted early access to Remdesivir in its medicines scheme for conditions with a high unmet medical need. The fact sheet for healthcare providers (https://www.gilead.com/-/media/files/pdfs/remdesivir/eua-fact-sheet-for-hcps.pdf) lists adverse events including raised liver transaminases, headache and nausea as common or very common. However, in clinical practice, these are generally mild and easily managed. In young adults (Humeniuk et al., 2020), the excellent safety profile of Remdesivir has been established in two phase I trials (single and repeat dose cohort studies), which included a diverse range of participants (male, female, and diverse ethnicities). Repeat dosing was given up to 14 days with 150mg once daily IV, which is a 50% higher dose and longer duration than current the current standard. In all cases, adverse effects were mild (with only grade 1 adverse events and no grade 3 or 4 adverse drug reactions). A number developed mild transaminitis (grade 1-2) but ALT and AST values universally returned to normal during the study. Additionally, in the final report of the randomized controlled trial of 1062 hospitalised COVID-19 patients, there was no difference in severe adverse events between Remdesivir (24.6%) and placebo (31.6%), nor grade 3 or 4 nor any other adverse events (Beigel et al., 2020). Thus, while its efficacy as pre-emptive treatment in the setting of the human challenge model has yet to be shown, it is unlikely that Remdesivir in the young adult age group treated with a short course leads to harm. These considerations should be explained to potential study participants.

2.9 Pre-clinical and clinical experience with REGEN-COV (Regeneron Monoclonal Antibody cocktail) for COVID-19

Monoclonal antibodies that recognise SARS-CoV-2 are believed to be able to block viral replication and either prevent or reduce the severity of disease. The leading example is from Regeneron^{TM}, which has developed 2 non-competing, high-affinity human IgG1 anti-SARS-CoV-2 monoclonal antibodies (mAbs), REGN10933 (casirivimab) and REGN10987 (imdevimab) [52, 53]. These mAbs bind specifically to the RBD of the SARS-CoV-2 spike glycoprotein and act as potent neutralising antibodies by blocking interaction with the host ACE2 receptor, which is responsible for viral uptake into host cells. REGN10933 and REGN10987 are intended to be used as a combination therapy (REGEN-COV) as they bind to distinct and non-overlapping regions of the RBD with the view to broaden cover against circulating viral variants (40). Additionally, *in vitro* studies have demonstrated that using this non-competing combination minimises the risk of escape mutation development due to selection pressure, with no escape mutants developing with the dual non-competing REGEN-COV mAB regime compared with development of escape mutations occurred when single mAb or dual competing mAb therapies were tested *in vitro* [53].

Clinical studies in both hospitalised and outpatient SARS-CoV-2 infected individuals are ongoing. However, interim results released from the phase 1 and 2 portions of a phase 1-3

double blind outpatient SARS-CoV-2 study (COV-2067) prompted the FDA to issue an emergency use authorisation (EUA) on 21st November 2020 for treatment of non-hospitalised patients with mild to moderate COVID-19 who are at high risk for progressing to severe disease and/or hospitalisation [55]. In this study, non-hospitalised participants received a single intravenous infusion of REGEN-COV within 3 days of having a positive SARS-CoV-2 PCR test. According to the EUA, 799 participants were randomized to receive one of two doses of REGEN-COV combination, either the 2,400 mg dose (n = 266) or the 8,000mg dose ((n = 267), or placebo (n = 266). Results from this study demonstrated a tolerable safety profile, with serious adverse events being numerically more frequent with placebo than REGEN-COV treatment (0.8% high dose REGEN-COV, 1.6% low dose REGEN-COV; 2.3% placebo). None of the SAEs were considered to be related to the study drug. Numerically more infusion reactions occurred with the REGEN-COV high dose compared to placebo but not with the low dose REGEN-COV regime (1.5% high dose; 0% low dose; 0.4% placebo). This study demonstrated that overall, the average daily change in viral load through day 7 (mean time-weighted average change from baseline) was a 0.36 log10 copies/mL greater reduction with REGEN-COV compared to placebo (combined dose groups; p=0.0003). The greatest benefit in viral load reduction was seen in individuals who had higher starting viral loads and those who were seronegative at baseline (i.e. slow immune responders) [54, 55].

Efficacy in the outpatient cohort was recorded as the number of medically attended events. Treatment with REGEN-COV reduced COVID-19 related medical visits by 57% through day 29 (2.8% combined dose groups; 6.5% placebo; p=0.024). This benefit was greater in those who had one or more risk factors for progression to severe COVID/hospitalisation with a reduction in medically attended events of 72% (combined dose groups; nominal p = 0.0065)[55]. Results showed no significant difference in virologic or clinical efficacy between the REGEN-COV high dose (8 grams) and low dose (2.4 grams). More recently, Regeneron have made public (not yet peer reviewed) data showing that the efficacy and safety of a 1.2g dose is comparable to that of the 2.4g dose. [56] This demonstrated that median time for symptom duration was reduced by 4 days in both 2.4g and 1.2g doses (10 v 14 days in placebo). Viral load reductions, hypersensitivity reactions and SAEs were comparable across both of these dose groups as well. Furthermore, the positive effect is strongest in those with high viral loads, which have been evident in the challenge model to date.

The applicability of this data to our cohort is limited. We are excluding any individuals with risk factors for severe COVID-19. However, it is clear that this is a safe and well-tolerated treatment with evidence of a significant reduction in medically attended events related to COVID-19. It would also be expected that a targeted treatment against SARS-CoV-2 infection would have greatest impact when given early in the course of infection. Given the deliberate inoculation of individuals with SARS-CoV-2, using a targeted pre-emptive or rescue therapy is an important ethical consideration recognised by the World Health Organisation in their regulatory framework for challenge models [57].

2.10 Human respiratory virus challenge studies in the UK

The UK is world-class in having extensive recent experience using models of respiratory viral challenge, and several of the study team have developed and/or used such models. At Imperial College London, Chris Chiu and Peter Openshaw began conducting experimental human infections with RSV in 2010, with the goal of establishing challenge studies in an academic setting. In 2015, the influenza challenge model was established at Imperial and together these models have been shown to be safe and well-tolerated as well as providing unique platforms for the study of immunity and immunopathogenesis during acute infection (Guvenel et al., 2020; Habibi et al., 2020; Jozwik et al., 2015). Imperial have now safely challenged >150 participants with these respiratory viruses and are currently the only academic group in Europe conducting these studies. The clinical research organisation hVIVO also has extensive experience in conducting respiratory virus challenge studies, mainly in the commercial sector. With a dedicated unit in East London and a large and experienced team that has conducted 56 studies with approximately 3000 volunteers inoculating with influenza and RSV to test a number of vaccine and therapeutic candidates, hVIVO has unparalleled expertise in running high-throughput human respiratory viral challenge studies (Bagga et al., 2013; DeVincenzo et al., 2014; DeVincenzo et al., 2010; Jones et al., 2009; Lambkin-Williams et al., 2016; McClain et al., 2016; Nguyen-Van-Tam et al., 2020; Wilkinson et al., 2012). Additionally, in developing this protocol, we have drawn on the expertise of a large group of UK experts with expertise in the ethics, regulation, virology, clinical implementation and interpretation of human infection challenges studies (see Key Protocol & Research Contributors). These include Robert Read (Southampton), whose extensive experience of initiating and developing new respiratory tract human infection challenge models, including genetically modified *Neisseria lactamica* and wild type *Bordetella pertussis* (de Graaf et al., 2020), and mitigation of risks against potentially hazardous challenge pathogens has been invaluable in study development.

2.11 Study Rationale

During 2021 it is likely that there will be widespread deployment of early generation Coronavirus vaccines in the UK. The need to refine and improve these vaccines will remain yet it will become increasingly difficult to test new vaccines in the field because (a) target groups will be already vaccinated, and (b) the natural incidence of disease will diminish.

A large number of SARS-CoV-2 vaccine candidates are at various stages of development internationally including some which have recently entered mid-to-late stage clinical testing in field studies. Under ordinary circumstances, vaccine development is a long process because of the time and risks involved in providing clinical data from field studies necessary to meet regulatory approvals. In order to attain a profound and timely impact on public health, there is an urgent need to select the most promising vaccines in the shortest possible timeframe.

Human challenge studies can provide a high precision model for the testing of vaccines and therapeutics as well as for studying host–pathogen interactions in small numbers of participants (generally about 25–100 people). Such studies have previously been conducted with many pathogens, including seasonal coronaviruses, Respiratory Syncytial Virus (RSV), rhinovirus and

both seasonal and pandemic influenza virus strains. A controlled human infection model for COVID-19 has been widely discussed in the scientific literature (Berry et al., 2020). No alternatives to this approach exist that can provide the same level of predictive value within a short timeframe using small numbers of participants. Data from animal models, while informative, have no guarantee of carrying over into humans and frequently do not. Primate models often vary between the species tested. Alternative study designs in humans, such as phase III field efficacy trials, would take many years to provide the same answers (Berry et al., 2020). During this time, deaths in the population would accumulate while waiting for months or years to gather such evidence.

The recent announcement of preliminary efficacy results of a SARS-CoV-2 vaccine by Pfizer/BioNTech does not negate the need for these studies. First, the capacity of this and other first-generation SARS-CoV-2 vaccines to reduce transmission as well as modify disease is unknown. Second, it is unclear whether this vaccine can protect against severe disease as well as it does against mild illness. It is also likely that these vaccines will perform less effectively in older adults and other high-risk groups, limitations which other vaccine candidates may overcome. There will therefore remain a critical need for improved vaccines to be developed and for vaccines to be compared in a controlled system so that these improvements can be accelerated.

Using these carefully selected endpoints, it should be possible using human infection challenge studies to identify vaccines which do or do not elicit any protective activity with potential to provide a health benefit, before investment of time, effort and resources in large scale field trials. This decreases the risk of late-stage failure. Well optimised human infection challenge studies also enable efficacy studies to be conducted during periods of low transmission (such as between pandemic waves) due to their guaranteed high infection rate. These studies will enable promising and innovative candidates to be prioritised even when they come from smaller academic or biotechnology groups that are unable to access costly field efficacy trials. While data from human infection challenge studies in young healthy adults may not always be directly translatable to populations at higher risk (such as elderly people with co-morbidities), a vaccine candidate tested by infection challenge of young adults that showed no effect on infection rate or viral shedding could reasonably be understood to have little protective efficacy in those with young healthy immune systems, and therefore would be even less likely to show efficacy in more high-risk populations with impaired immunity. The controlled nature of human infection challenge will also permit direct comparison between different vaccine candidates to prioritise the most promising.

In addition, human infection challenge can contribute to the identification of correlates and mechanisms of protection against infection and shedding of virus in vaccinated volunteers, as well as the durability of protection in seropositive individuals with documented prior wild type infection.

Thus, we will establish a SARS-CoV-2 human infection challenge system that will let us test vaccines and treatments reliably in a controlled setting with a participant group that is very unlikely to suffer a severe disease. In turn, these interventions may save the lives of a much larger section of the population, for whom COVID-19 is potentially serious. The quicker

development of vaccines that can protect everyone using this strategy balances the managed risk to a small number of study participants with potential benefit to millions, who might otherwise suffer while more effective interventions are being developed.

This first virus characterisation study will:

- Enable the immediate start of a study of protective immunity at University of Oxford funded by the Department of Health and Social Care. We are collaborating closely with Helen McShane, who will lead that study using the same challenge virus and closely aligned clinical and laboratory methods. Around 2 weeks after our dose escalation study of seronegative individuals begins if no safety signals are seen in the sentinel groups, she will start parallel dose escalations in previously-infected seropositive volunteers to show safety in this participant group. Once the optimal dose is established, she will proceed to inoculate groups of participants with a history of previous COVID-19 and different levels of antibody and T cells to test infection rates and viral shedding. Data will be shared with our seronegative study to establish the protection afforded by previous infection. Thus, by July/August 2021, we will have determined whether immune responses to previous infection are protective and started to define the levels of pre-existing immunity required for protection. These will have immediate impacts, such as determining whether to allow return to unfettered travel and normal daily activities, as well as critical information to determine the need and timing of vaccination and re-vaccination in the context of previous infection.
- Enable studies of existing and novel antiviral drugs. We are in discussions with Regeneron and Gilead to implement studies to test the efficacy of different dosing regimes, including prophylaxis and early pre-emptive treatment. These will have particular impacts on outbreaks in hospitals and care homes to prevent clusters of severe disease.
- Lead directly to comparative studies of next-generation SARS-CoV-2 vaccine candidates (funded by the Vaccines Taskforce) by establishing the model ready for vaccine testing, i.e. determining the dose of virus to use and providing data to accurately conduct sample size powering calculations for the vaccine challenge studies. These vaccine challenge studies will compare vaccines based on differing technologies against licensed vaccines, with formulations and regimens (such as low or single-dose) that will improve efficacy (such as seen with the improved results following low-dose priming of the AstraZeneca/Oxford vaccine) and global accessibility (by making the same amount of vaccine go further and delivered more simply). If these relied on field study approaches, direct comparisons could not be made (due to heterogeneity of natural infection events), and efficacy data could potentially take years to be obtained and therefore less relevant to the current pandemic. Furthermore, these challenge studies will be able to accurately ascertain if the vaccines are effective at preventing asymptomatic infection, which is not possible to do with field trials. This is crucial as asymptomatically infected individuals are considered to be driving the continuation of the pandemic, being unable to self-isolate as they are unaware of their infectious status. Human challenge is a key component of the Vaccines Taskforce strategy in order to deliver safe and

effective vaccines and therapeutics to the UK population as well as for future pandemics. The UK government is in active discussion with vaccine companies to initiate these studies by May 2021.

2.12 Research Hypotheses

1. SARS-CoV-2 infection in experimentally infected healthy young adult volunteers is safe and well tolerated.
2. Experimental COVID-19 allows early viral replication to be measured and enables monitoring of innate and adaptive immune responses and radiological changes which act as surrogates of protection and disease.

2.13 Benefit/Risk Assessment

Risk determination

All study procedures involve no more than minimal risk to participants. Similar procedures have been used for many years without severe adverse effects. These include blood and nasal sampling; participants will be counselled as follows:

Blood draws: risks include discomfort as the needle goes through the skin and/or bruising. Infection, excess bleeding, clotting, or fainting are also possible, although unlikely.

Nasosorption strips (a soft strip placed up the nose for one minute) and nasopharyngeal swabs: may tickle, make their eyes water or be slightly uncomfortable. They should not be painful. The oral swabs are not painful.

The risks of SARS-CoV-2 infection in the study population are explained in Background to Research.

Potential adverse effects of chest x-ray and CT

Each volunteer enrolled in the study will have a single chest X-ray at day -2/-1 prior to inoculation, which is to act as a baseline for further imaging that may be necessary and to ensure that no significant pulmonary infection or other pathology is present at the start of the study. The estimated dose will be 0.02 mSv which is approximately equivalent to 3 days of average natural background radiation and carries a risk of inducing a cancer of approximately 1:1,000,000 based on risk factors for a healthy adult. This is classified as minimal risk (HPA-CRCE-028).

The CT chest scans will be associated with a higher radiation dose. The delivered dose varies but is likely to be about 1.5 mSv per scan, which is approximately equivalent to 18 months of average natural background radiation and carries a risk of inducing a cancer of around 1:1,000-10,000. This is classified as low risk (HPA-CRCE-028).

Pregnant or breastfeeding women will be excluded from the study. The known risks to participants are detailed in

Table 6. However, there may also be risks that are unforeseen and not anticipated (e.g. unknown allergies). Every effort will be made to monitor the health of the participants to ensure that such risks are minimised.

Table 6: Risk Assessment

Potential Risk of Clinical Significance	Rationale for Risk	Mitigation Strategy
**Study Intervention**		
Intravenous dosing with SARS-CoV-2 intervention treatments	Local signs and symptoms associated with the cannula insertion (peripheral access) and drug administration may include erythema, swelling/induration, phlebitis, haematoma and pain/tenderness at the insertion site.	Local reactions will be monitored but are generally short-term and do not require treatment. Aseptic technique used for cannula insertion and hypoallergenic dressings.
	Other side effects include rash	Side effects will be monitored but are generally short-term and do not require treatment.
	Other less common but serious side effects reported include anaphylaxis and anaphylactic shock and other severe acute hypersensitivity reactions	Medications (e.g. epinephrine) will be available in the clinic to treat serious allergic reactions promptly. Participants with a known severe allergy, or history of anaphylaxis or other serious adverse reactions will be excluded from the study. The study site should have medical treatment available in case of severe allergic reactions.
	Systemic exposure to SARS-CoV-2 intervention treatments	To minimise any risks associated with systemic exposure to SARS-CoV-2 intervention treatments, male and female participants must be on highly effective contraception methods during the study and up to 90 days post dosing with SARS-CoV-2 intervention treatments.
Intravenous Dosing with Remdesivir	Lack of pre-clinical reproductive toxicity evaluation as well as clinical toxicity evaluations in pregnancy due to target population indication (high-risk children <24 months) of Remdesivir and requirements for licensing for indication. Although reproductive toxicity studies are not considered appropriate for the study intervention.	Participants will be advised to use appropriate methods of contraception during the study and for 90 days after administration of Remdesivir for male participants (equivalent to the sperm life cycle) and 30 days for female participants (equivalent to the length of a menstrual cycle).
	Remdesivir may interfere with immunological-based SAR-	Discharge from quarantine will utilise a PCR based test instead of an

Potential Risk of Clinical Significance	Rationale for Risk	Mitigation Strategy
	CoV-2 diagnostic tests such as some antigen detection-based assays. In addition, Remdesivir inhibits virus replication in cell culture, and therefore may also interfere with viral culture assays. Remdesivir does not interfere with reverse transcriptase-polymerase chain reaction-based assays. Assay interference could lead to false-negative SAR-COV-2 diagnostic test results. Therefore, diagnostic test results, when obtained, should be used in conjunction with clinical findings to guide medical decisions	antigen test.<br>PCR based tests will be used to confirm the incidence of infection and to quantify virus shed by infected participants.<br>Implications for culture-based quantification of SAR-CoV-2 will be assessed.
**Study procedures**		
Blood Sampling	Pain or bruising at the site where blood is drawn.	Blood samples will be obtained by a trained professional.
	Syncope (fainting) can occur following or even before any blood draw as a psychogenic response to the needle insertion.	Blood samples will be obtained by a trained professional and procedures will be put in place to avoid injury from fainting.
	There is a possibility that in the process of collecting blood a nerve may be injured.	Procedure to be performed by qualified personnel.
	Blood tests performed to address the health of the participants at screening and during the study may indicate that a participant has an infection that he/she was not previously aware of (such as HIV or hepatitis) or an unexpected illness.	The study doctor will provide the participant's general practitioner (GP), or doctor with a referral letter if the participant agrees.
Nasal sampling	Collection of nasopharyngeal swabs may cause discomfort, sneezing, watery eyes, irritated nose or nose bleeding.	Sample collection will be performed by appropriately qualified and trained study staff to minimise the discomfort.

Potential Risk of Clinical Significance	Rationale for Risk	Mitigation Strategy
**SAR-COV-2 infection from inoculation**		
SAR-COV-2 infection & severe complications	Chance of becoming infected with SAR-CoV-2. Typical SAR-CoV-2 illness: abrupt onset of fever, malaise, myalgia (muscle aches), alteration or loss of smell, cough and sore throat.<br>Severe SAR-CoV-2 infections are known to occur rarely in both infants and adults. In adult populations, multiple factors including older age, are independently associated with severe SAR-CoV-2 complications including chronic co-morbidities and significant immune compromise.<br>The study virus, like many viruses, can cause more substantial health issues such as myocarditis (inflammation or damage to the heart muscle). However, the chance of this resulting in serious or permanent changes is rare, as most cases are minor and resolve without any lasting changes.	The safety profile of the SAR-COV-2 in young healthy adults has been described in the Background to Research. SAR-CoV-2 infection in healthy young adults usually resolves without treatment within 11 to 14 days. Fatigue and loss of smell may be protracted but in almost all cases resolve completely.<br>Strict inclusion and exclusion criteria, combined with stringent health screening, will apply to ensure only healthy young adults with no known risk factors for severe COVID-19 are enrolled in this study.<br>There will be a daily medical monitoring in a quarantine unit for at least 14 days post-challenge.<br>Qualified medical and nursing staff in the quarantine unit will regularly monitor for and manage any symptoms.<br>Participants will be closely followed up while being in quarantine. Electrocardiogram will be performed, and cardiac enzymes will be tested at least 4 days, 7 days and 11 days post-viral challenge. Cardiac enzymes are tested daily at screening, during quarantine and during follow up.
	Transient increase in alanine aminotransferase (ALT) or aspartate aminotransferase (AST) without clinical presentation, with a good prognosis upon improvement of infection.	ALT and AST will be monitored.
Transmission of SAR-COV-2 to participants' close contacts	SAR-CoV-2 virus in nasal secretions can cause infection in close contacts.	Discharge criteria have been established, as detailed in Section 5.18.5 to reduce risk of transmission outside of the quarantine unit.
	Passing the SAR-CoV-2 Challenge Virus to others, including	To reduce the risk, participants will be asked to avoid, where possible,

Potential Risk of Clinical Significance	Rationale for Risk	Mitigation Strategy
	vulnerable people (see below for definition of vulnerable populations).	contact with vulnerable people for 14 days after they leave quarantine (Section 2.13.1).
	Passing the SAR-COV-2 Challenge Virus to study staff.	Clinic staff in contact with participants in the quarantine unit will require to wear Personal Protective Equipment (PPE) as per local SOPs, to avoid the transmission of the SAR-CoV-2 Challenge Virus to study staff and to prevent the risk of cross-contamination by a viral disease being brought up into the unit by staff.<br>Robust infection control measures will be implemented to prevent transmission, including disinfection of equipment, sample dispatch, infection control instructions to volunteer concerning their belongings.
Risk of reactivation of herpes infection.	If a participant ever had a herpes infection (e.g., cold sores, genital herpes or shingles), there is a small possibility that this infection could return after challenge.	Participants will be instructed to inform the study staff if they currently have an active herpes infection or have had one during the 30 days before enrolment.
Please reference – Reference Safety Information for Remdesivir and REGEN-COV for further details.		

2.13.1 Vulnerable persons

For the purposes of possible contact, a vulnerable individual is a person who has close or household (i.e., share the same apartment or house) high-risk contacts including but not limited to:

- Persons ≥65 years of age
- Children ≤2 years of age
- Residents of nursing homes
- Women who are pregnant or who are trying to become pregnant.
- Persons of any age with significant chronic medical conditions such as:
  - Chronic pulmonary disease (e.g., severe asthma, Chronic Obstructive Pulmonary Disease (COPD))
  - Chronic cardiovascular disease (e.g., cardiomyopathy, congestive heart failure, cardiac surgery, ischemic heart disease, known anatomic defects)
  - Contacts that required medical follow-up or hospitalisation during the past 5 years because of chronic metabolic disease (e.g., insulin dependent diabetes mellitus, renal dysfunction, haemoglobinopathies)
  - Immunosuppression or cancer
  - Neurological and neurodevelopmental conditions (e.g., cerebral palsy, epilepsy, stroke, seizures)

2.13.2 Benefit assessment

Healthy participants in clinical studies will not receive direct benefit from treatment during their participation. Participants may benefit from a general health check at Screening. Benefit may also be derived from the medical evaluations and assessments associated with study procedures. In addition, participants are contributing to the process of developing new therapies in an area of unmet medical need.

2.13.3 Overall benefit: risk conclusion

Taking into account the measures taken to minimise risk to participants in this study, the potential risks are justified by the anticipated benefits linked to the evaluation of a viral challenge model which is being developed to then be able to use the model as an efficient method of testing vaccine efficacy. Furthermore, the viral challenge model will also subsequently contribute to the identification of mechanisms of protection against viral infection and shedding of virus in vaccinated volunteers. The challenge model may be valuable in establishing of the durability of protection in seropositive individuals with wild type infection.

2.14 Objectives and Endpoints

To develop the first human challenge model for wild-type SARS-CoV-2 virus infection in healthy human subjects.

A prospective open label dose escalation study in healthy participants to establish the safety and identify the infectious dose of SARS CoV-2 challenge virus needed to induce infection and active viral replication in ≥50 of participants (ideally between 50% to 70%) with minimal or no disease in healthy young adults after intranasal inoculation.

The study will comprehensively describe the attack rate, host immune responses, viral kinetics and clinical disease induced by pre-emptively treated infection in this model.

OBJECTIVES	ENDPOINTS
**PRIMARY**	
To identify a safe and infectious dose of wild type SARS-CoV-2 in healthy volunteers, suitable for future intervention studies	To evaluate the safety of wild type SARS-CoV-2 challenge in healthy participants by assessing:<br>• Occurrence of unsolicited AEs within 30 days post-viral challenge (Day 0) up to Day 28 follow up.<br>• Occurrence of SAEs related to the viral challenge from the viral challenge (Day 0) up to Day 28 follow up.
	To identify a SARS-Cov-2 inoculum dose that safely induces laboratory confirmed infection in ≥50% of participants (ideally between 50% and 70%). Laboratory confirmed infection is defined as:<br>• Two quantifiable greater than lower limit of quantification (≥LLOQ) RT-PCR measurements from mid turbinate and/or throat samples, reported on 2 or more consecutive timepoints, starting from 24 hours post-inoculation and up to discharge from quarantine.
**SECONDARY**	
To further assess SARS-CoV-2 viral infection rates in upper respiratory samples in healthy volunteers, by	• To assess the incidence of laboratory confirmed infection rates using a) mid turbinate samples, b) throat swabs, and c) both mid turbinate and throat swabs, as defined by:<br>• Variant 2: Occurrence of at least two quantifiable (≥LLOQ) RT-PCR measurements, reported on 2 or more consecutive timepoints, starting from 24 hours post-inoculation and up to discharge from quarantine.<br>• Variant 3: Occurrence of at least two detectable (≥LLOD) RT-PCR measurements, reported on 2 or more consecutive timepoints, starting from 24 hours post-inoculation and up to discharge from quarantine.

inoculum dose	• Variant 4: Occurrence of at least one quantifiable (≥LLOQ) SARS-CoV-2 viral cell culture measurement, starting from 24 hours post-inoculation and up to discharge from quarantine.
To assess the incidence of symptomatic SARS-CoV-2 infection, in healthy volunteers, by inoculum dose	• To assess the incidence of lab-confirmed symptomatic SARS-CoV-2 infection using a) mid turbinate samples, b) throat swabs, and c) both mid turbinate and throat swabs, defined as:<br>• Variant 1:<br>o Occurrence of at least two quantifiable (≥LLOQ) RT-PCR measurements, reported on 2 or more consecutive timepoints, starting from 24 hours post-inoculation and up to discharge from quarantine, AND<br>o Either one or more positive clinical symptoms of any grade from two different categories in the symptom scoring system (Upper Respiratory, Lower Respiratory, Systemic), or one Grade 2 symptom from any category<br>• Variant 2:<br>o Occurrence of at least two detectable (≥LLOD) RT-PCR measurements, reported on 2 or more consecutive timepoints, starting from 24 hours post-inoculation and up to discharge from quarantine, AND<br>o Either one or more positive clinical symptoms of any grade from two different categories in the symptom scoring system (Upper Respiratory, Lower Respiratory, Systemic), or one Grade 2 symptom from any category<br>• Variant 3:<br>o Occurrence of at least one quantifiable (≥LLOQ) SARS-CoV-2 viral cell culture measurement, starting from 24 hours post-inoculation and up to discharge from quarantine, AND<br>o Either one or more positive clinical symptoms of any grade from two different categories in the symptom scoring system (Upper Respiratory, Lower Respiratory, Systemic), or one Grade 2 symptom from any category
To assess the SARS-CoV-2 viral dynamics in upper respiratory samples (AUC, peak, duration, incubation period) in	To assess the viral dynamics using a) mid turbinate samples, and b) throat swabs, as measured by:<br>• Area under the viral load-time curve (VL-AUC) of SARS-CoV-2 as determined by qRT-PCR, starting from 24 hours post-inoculation and up to discharge from quarantine.<br>• Peak viral load of SARS-CoV-2 as defined by the maximum viral load determined by quantifiable (≥LLOQ) qRT-PCR measurements, starting from 24 hours post-inoculation and up to discharge from quarantine<br>• Duration of SARS-CoV-2 quantifiable (≥LLOQ) qRT-PCR measurements, starting from 24 hours post-inoculation and up to discharge from quarantine.

healthy volunteers, by inoculum dose	Duration is defined as the time (hours) from the first quantifiable of the two viral quantifiable positives used to assess infection until first confirmed undetectable assessment after their peak measure (after which no further virus is detected).<br>• Incubation period of SARS-CoV-2 qRT-PCR measurements. Incubation period is defined as the time (hours) from inoculation to the first quantifiable of the two viral quantifiable positives used to assess infection, starting from 24 hours post-inoculation and up to discharge from quarantine.<br>The above endpoints will also be evaluated using quantitative cell culture.
To assess the SARS-CoV-2 induced symptoms, in healthy volunteers, by inoculum dose	• Sum total symptoms diary card score: sum total clinical symptoms (TSS) as measured by graded symptom scoring system, starting one day post-viral challenge (Day 1) up to discharge from quarantine<br>• Area under the curve over time (TSS-AUC) of total clinical symptoms (TSS) as measured by graded symptom scoring system (categorical and visual analogue scales), starting one day post-viral challenge (Day 1) up to discharge from quarantine.<br>• Peak symptoms diary card score: peak total clinical symptoms (TSS) as measured by graded symptom scoring system (categorical and visual analogue scales, starting one day post-viral challenge (Day 1) up to discharge from quarantine<br>• Peak daily symptom score: Individual maximum daily sum of Symptom score starting one day post-viral challenge (Day 1) up to the end of quarantine.<br>• Number (%) of participants with Grade 2 or higher symptoms
To assess the incidence of SARS-CoV-2 illness, in healthy volunteers, by inoculum dose	The incidence of:<br>• Upper Respiratory Tract illness (URT)<br>• Lower Respiratory Tract illness (LRT)<br>• Systemic illness (SI)<br>• Febrile illness (FI)<br>• Proportion of Subjects with Grade 3 symptoms on any occasion at any time from the last assessment on Day 0 to quarantine discharge<br>• Proportion of Subjects with Grade 2 or higher symptoms on any occasion at any time from the last assessment on Day 0 to quarantine discharge<br>• Proportion of Subjects with Grade 2 or higher Symptoms on two separate occasions at any time from the last assessment on Day 0 to quarantine discharge<br>• Proportion of Subjects with any symptom (grade >=1) on any occasion at any time from the last assessment on Day 0 to quarantine discharge<br>• Proportion of Subjects with any symptom (grade >=1) on two separate occasions at any time from the last assessment on Day 0 to quarantine

	discharge
**TERTIARY**<br>Tertiary/exploratory endpoints include, but are not limited to;	
To explore the safety of wild type SARS-CoV-2 human challenge model in healthy adults	To explore safety related measures of wild type SARS-CoV-2 challenge in healthy participants by assessing:<br>• Changes in smell (anosmia/parosmia) and cognition through infection<br>• Pulmonary changes due to experimental infection, as measured by:<br>o High-resolution CT<br>o Spirometry (FEV1, FVC)<br>o Forced Oscillatory Technique (FOT)<br>• Association of ABO blood group and susceptibility to infection<br>• Occurrence of haematological and biochemical laboratory abnormalities during the quarantine period.<br>• Use of concomitant medications within 30 days post-viral challenge (Day 0 up to Day 28 follow up).
To explore the SARS-CoV-2 viral infection rates in saliva in healthy volunteers, by inoculum dose	To measure the laboratory confirmed infection rates, as defined by:<br>• Occurrence of at least two quantifiable (≥LLOQ) RT-PCR measurements, reported on 2 or more consecutive timepoints, starting from 24 hours post-inoculation and up to discharge from quarantine.<br>• Occurrence of at least two detectable (≥LLOD) RT-PCR measurements, reported on 2 or more consecutive timepoints, starting from 24 hours post-inoculation and up to discharge from quarantine.<br>• Occurrence of at least one quantifiable (≥LLOQ) SARS-CoV-2 viral cell culture measurement, starting from 24 hours post-inoculation and up to discharge from quarantine.
To explore the SARS-CoV-2 viral dynamics in saliva in healthy volunteers, by inoculum dose	To assess viral dynamics, as defined by:<br>• Area under the viral load-time curve (VL-AUC) of SARS-CoV-2 as determined by qRT-PCR measurements in saliva, starting from 24 hours post-inoculation and up to discharge from quarantine.<br>• Peak viral load of SARS-CoV-2 as defined by the maximum viral load determined by quantifiable qRT-PCR measurements in saliva, starting from 24 hours post-inoculation and up to discharge from quarantine.<br>• Duration of SARS-CoV-2 quantifiable qRT-PCR measurements in saliva, starting from 24 hours post-inoculation and up to discharge from quarantine. Duration is defined as the time (hours) from first the quantifiable of the two viral quantifiable positives used to assess infection until first confirmed

	undetectable assessment after their peak measure (after which no further virus is detected).<br>• Incubation period of SARS-CoV-2 qRT-PCR measurements in saliva. Incubation period is defined as the time (hours) from inoculation to the first quantifiable of the two viral quantifiable positives used to assess infection, starting from 24 hours post-inoculation and up to discharge from quarantine.<br>• The above endpoints may also be evaluated using quantitative cell culture.
To explore the host-pathogen relationship in the SARS-CoV-2 human challenge model in healthy adults	The primary, secondary, and tertiary endpoints may be explored in relation to immunological levels at baseline and after SARS-CoV-2 challenge. Assays performed on serum and mucosal samples may include, but are not limited to:<br>• Humoral immunity / systems serology SARS-CoV-2 (for example: SARS-CoV-2 neutralizing titres, ELISAs to IgG, IgM, IgA, sIgA, ADCC)<br>• Proteomic levels and changes (for example, cytokine and chemokines)<br>• Cellular cell quantification and quality of immunity (for example T and B cell frequencies, phenotypes and functionality assays, ELISPOTs, ICS, cytokine/chemokine responses)<br>• Transcriptome levels and changes (for example, RNAseq, single cell RNAseq, microarray, PCR)<br>• Human genomics in relation to SARS-CoV-2 susceptibility, infection (e.g. HLA typing, SNPs, GWAS)<br>• Viral genomics to assess the possible emergence of mutations in SARS COV-2 over the duration of the study<br>• Viral genomics in relation to SARS-CoV-2 population changes through infection<br>• Microbiome analysis in relation to viral infection, disease and susceptibility (e.g. PCR, NGS, 16s rRNA)
To explore the Minimal Clinically Important Difference (MCID) in instrument change	To explore the average amount of instrument-assessed change for all participants who rate themselves as "a little better" or "somewhat better". Instruments include but are not limited to:<br>• Symptom diary card (Categorical Scale)<br>• Symptom diary card (Visual Analogue Scale)
To explore environmental contamination in the SARS-CoV-2 human	To explore the environmental contamination of SARS-CoV-2 as a result of infection in participants, as measured by:<br>• Air sampling for virus detection and quantification<br>• Exhaled breath sampling with the use of a face mask for virus detection and quantification

challenge model in healthy adults	• Surface swabbing for virus detection and quantification

3. Study Design

A schematic overview of the study design is presented in Figure 1.

3.1 Overall Design

This is a dose finding human experimental infection study in healthy adults 18 to 30 years of age inclusive with no known risk factors for severe COVID-19, using a GMP-produced SARS-CoV-2 wild type strain.

The study is divided into two phases, outpatient phase and quarantine phase (confinement phase).

Specific procedures to be performed during the study, as well as their prescribed time points and associated visit windows, are outlined in the SoA in Table 1**.** Details of each procedure are provided in Section 5.

Where multiple assessments are scheduled for collection at the same time point, assessments can be performed before or after the specified time point, within their permitted time windows, as required. If blood sampling or vital signs measurement is scheduled for the same time point as ECG recording, the procedures will be performed in the following order: ECG(s), vital signs, blood draw. Actual dates and times of all assessments will be recorded in the CRF.

3.2 Screening Phase

Screening of potential participants will take place in two stages with an initial screening visit, followed by a study specific remote consultation to go through the full study participant information (if not already done at the screening visit), thus allowing the informed consent form (ICF) to be signed at admission to the quarantine unit, following adequate time for ICF and participation in the study to be considered. Screening visits will take place at hVIVO QMB Innovation Centre.

Between Day -90 to Day -2 potential participants will be screened under a separate study-specific screening protocol using a screening ICF and advertising material that has been approved by the Research Ethics Committee (REC) and Health Research Authority (HRA). Screening activities under the separate screening protocol may continue up until subjects sign the study specific consent. Recruitment will be done through a number of channels:

- Approved advertising, including social media
- hVIVO volunteer database (Volunteers already registered with any other hVIVO database may be contacted to determine their interest in participating in SARS-CoV-2 research.)
- Referral
- Organic search (e.g. via Google or other search engines)

Where volunteers who have previously registered or been screened by hVIVO have given written consent, their data may be used as part of the screening process. Volunteers that do not enroll in this study for any reason will be recorded as screen failures and may be eligible to participate in other studies. If they consent, the results of their screening questionnaires and some or all screening tests may be used to assess their suitability for other ethically-approved studies, if they fall within the screening window.

The screening process will consist of several stages: registration (if not already registered); webform questionnaire; telephone questionnaire; screening visit.

Volunteers already registered with any other hVIVO database may be contacted to determine their interest in participating in SARS-CoV-2 research. Others interested in participating will be asked to register their details on a SARS-CoV-2 specific web page UKcovidchallenge.com. All advertising and media will direct volunteers to this web page to register their interest. If they fit the age criteria, they will be contacted and guided to a webform questionnaire. They will then be contacted by telephone to complete a telephone questionnaire to collect further demographic and health information.

If inclusion/exclusion criteria are provisionally met based on answers to these questions, an appointment for screening will be scheduled and a confirmation email plus both screening and full study Participant Information Sheets (PIS)/Informed Consent Forms (ICF) will be sent. Screening appointments will be conducted at hVIVO. At the screening visit, the PIS/ICF will also be discussed with them by the study doctor or nurse. Volunteers will have their QCOVID risk scores calculated and explained to them. When the subject has had enough time to consider their participation in this study, ask any questions they may have, and only when they have agreed to take part will they be asked to read, sign and date the relevant consent form in the presence of the study doctor or nurse who will also sign the consent form. Written consent will be obtained prior to any history-taking, examination or tests are carried out. A copy will be kept in the research file, a copy given to the patient and a copy put into their medical notes.

Following informed consent, medical history, examination and screening assessments will be undertaken (see SoA, Table 1). The volunteer's medical history will be requested from their GP and reviewed to assess suitability. Subjects may be invited for repeat assessment where / if required at the PI's discretion. GP medical histories that have been previously received by hVIVO and assessment results from earlier quarantine admissions (if the subject did not go on to be inoculated) may be used for screening purposes if obtained within the screening window.

3.3 Study Specific Consent

Following the screening phase, suitable participants willing to take part in the study will be provided with a study specific Participant Information Sheet (PIS) and Informed Consent Form (ICF), if this was not available at the screening visit. In all cases, the PIS/ICF will be provided prior to admission to the quarantine unit to allow adequate time for participants to read and consider the PIS/ICF, discuss the study with family members and/or friends and ask questions about the research. A follow-up appointment (conducted remotely due to the COVID-19 pandemic) will be made for the participant to go through the details of the study and PIS/ICF

with a trained study team member to ensure understanding of the risks and unknowns of the study and given an opportunity to ask further questions. A multiple-choice quiz will then be conducted to check the volunteer's understanding of the study rationale, procedures and risks. If they do not pass the quiz, the study team member will check the incorrect quiz answers and re-review with the volunteer the relevant ICF sections to ensure understanding.

On Day -2/-1, the participant will be admitted to the quarantine unit. Prior to any study specific procedures taking place, participants will have a further opportunity to discuss the research protocol with a trained study team member before signing the full study specific ICF. The full consent process will be audio recorded.

To minimise the risk of admitting someone incubating SARS-CoV-2, a risk-based approach based on the Royal Free London NHS Trust Standard Operating Procedures for elective admissions will be used prior to enrolment:

1. Participants will be advised to stay at home for the requisite number of days before admission, not leaving the house for any reason other than an emergency or to have a COVID test.
2. A swab test will be sent to the participant. This must be taken within 72 hours before admission and shown to be negative for SARS-CoV-2.
3. On admission, they will be met by staff wearing PPE and will go directly into their own en-suite room.
4. During their stay, they will only have contact with staff wearing PPE, thus mitigating onward transmission if they have undiagnosed infection.
5. Participants will then be re-tested for SARS-CoV-2 (along with a panel of other respiratory viruses and bacteria) with a negative result required before inoculation.

It is recognized that the Royal Free London NHS Trust Standard Operating Procedures for elective admissions may change over time, and as such the risk mitigating steps listed above may be adjusted to align with the procedures, as appropriate.

3.4 Quarantine Phase

Subjects will stay overnight for a period of at least 16 days, from 1 to 2 days before the viral challenge, to at least the 14th day after viral challenge (see discharge criteria, Section 5.18.5). This period of quarantine has been chosen to eliminate the possibility of subjects in the study transmitting the virus to anyone not involved in the study (i.e. family, household contacts, and the wider community). During the quarantine period, all study procedures will take place in the Royal Free Hospital Quarantine Unit designated for this study.

Around one week before each challenge cohort, a panel of leaders of the North Central London (NCL) Adult Critical Care Network will meet with the CI, PI and clinical team to assess clinical capacity as follows:

- Using their direct access to daily capacity data across the network, plus local and national projections, the panel will provide an evidence-based opinion on clinical

capacity at the start of each challenge group as well as the weeks to come, and advise whether it is safe to commence.

- This will also include review of non-ITU bed state and radiology capacity.
- This decision will be recorded in the Trial Master File and no dosing will take place without a favourable opinion from this panel.

3.5 Follow Up Phase

There will be telephone calls every 2-3 days between discharge from Quarantine and Day 28 (+/- 3 days). During follow up phase all participants will be required to attend 5 clinic visits at the hVIVO site (QMB) on Day 28 (+/- 3 days), Day 90 (+/- 7 days), Day 180 (+/- 14 days), Day 270 (+/- 14 days) and Day 360 (+/- 14 days), as specified in the SoA, Table 1.

3.6 End of Study Definition

A participant is considered to have completed the study if he/she has completed all phases including the last scheduled visit shown in the SoA Day 360 (± 14 days) or the last unscheduled visit as applicable. If a safety visit is required after the last scheduled visit, this will be at the PI's discretion as a duty of care, e.g., repeat spirometry or laboratory tests. These discretionary follow-up visits will not be considered part of the trial data unless they represent follow-up and closure on an AE or serious adverse event (SAE) identified during the trial period.

The end of the study is defined as the date of the last visit of the last participant in the study. See Section 4 for procedures in the event of Discontinuation of study intervention or volunteer withdrawal.

Subjects will return to the care of their own GP following discharge from the quarantine unit. Should any abnormal assessment or finding be reported that is deemed clinically significant the subjects GP will be informed.

3.7 Study Population

Prospective approval of protocol deviations to recruitment and enrolment criteria, also known as protocol waivers or exemptions are not permitted in this study.

3.8 Inclusion Criteria

Participants are eligible to be included in the study only if all of the following criteria apply:

NO	INCLUSION CRITERIA
1	An informed consent document signed and dated by the participant and the Investigator.

2	Male or female, age between 18 and 30 years inclusive (at the time of consent)
3	Seronegative to the challenge virus SARS-CoV-2, no history of SARS-CoV-2 infection and no previous participation in a SARS-CoV-2 vaccine trial.
4	Female participants with a documented menstrual period within 28 days before the inoculation (unless using a contraceptive method that suppressed menstruation as indicated in the study protocol) and willing and able to use contraception as described in the study protocol from 2 weeks before the scheduled date of viral challenge until 6 months after receipt of the final dose of study virus or intervention treatment (whichever occurs last). Negative urine pregnancy tests will be required at screening and on day 0 prior to inoculation. On admission to the quarantine unit a Negative serum beta human chorionic gonadotropin (β-hCG) is required.<br>**<u>Contraceptive requirements:</u>**<br>Established use of hormonal methods of contraception described below (for 2 weeks prior to the first study visit). When hormonal methods of contraception are used, male partners are required to use a condom with a spermicide:<br>1) combined (oestrogen and progestogen containing) hormonal contraception associated with inhibition of ovulation:<br>i. oral<br>ii. intravaginal<br>iii. transdermal<br>2). progestogen-only hormonal contraception associated with inhibition of ovulation:<br>i. oral<br>ii. injectable<br>iii. implantable<br>3) Intrauterine device (IUD)<br>4). Intrauterine hormone-releasing system (IUS)<br>5). Bilateral tubal ligation<br>6). Male sterilisation (with the appropriate post vasectomy documentation of the absence of sperm in the ejaculate) where the vasectomised male is the sole partner for that woman.<br>7). True abstinence - sexual abstinence is considered a highly effective method only if defined as refraining from heterosexual intercourse during the entire period of risk associated with the study treatments. The reliability of sexual abstinence needs to be evaluated in relation to the duration of the clinical trial and the preferred and usual lifestyle of the subject.

5	Men who are willing to use one of the contraception methods described in the study protocol, from the time of the date of viral challenge, until 6 months after receipt of the final dose of study medication (if applicable).<br>**<u>Contraceptive requirements:</u>**<br>a. Use a condom with a spermicide to prevent pregnancy in a female partner or to prevent exposure of any partner (male and female) to the study virus or intervention treatment.<br>b. Male sterilisation with the appropriate post vasectomy documentation of the absence of sperm in the ejaculate (*please note that the use of condom with spermicide will still be required to prevent partner exposure).* This applies only to males participating in the study.<br>c. In addition, for female partners of child bearing potential, that partner must use another form of contraception such as one of the highly effective methods mentioned above for female subjects.<br>d. True abstinence - sexual abstinence is considered a highly effective method only if defined as refraining from heterosexual intercourse during the entire period of risk associated with the study treatments. The reliability of sexual abstinence needs to be evaluated in relation to the duration of the clinical trial and the preferred and usual lifestyle of the subject.<br><br>In addition to the contraceptive requirements above, male subjects must agree not to donate sperm following discharge from quarantine until 6 months after the date of study virus receipt or intervention treatment (whichever occurs last).
6	In good health with no history of clinically significant medical conditions (as described in Exclusion criteria) that would interfere with subject safety, as defined by medical history, physical examination and routine laboratory tests, ECG, and Chest X-Ray and determined by the Investigator at an admission evaluation.
7	Subjects will have a documented medical history either prior to entering the study and/or following medical history review with the study physician at screening
8	Willing and able to commit to participation in the study

3.9 Exclusion Criteria

Participants are excluded from the study if any of the following criteria apply:

NO	STANDARD EXCLUSION CRITERIA
Any potential subject who meet any of the criteria below will be excluded from participating in this study.	
**Clinical history**	
1.	History or evidence of any clinically significant or currently active cardiovascular, (including thromboembolic events), respiratory, dermatological, gastrointestinal, endocrine, haematological, hepatic, immunological, rheumatological, metabolic, urological, renal, neurological, psychiatric illness. Specifically:<br>a) Subjects with any history of physician diagnosed and/or objective test confirmed asthma, chronic obstructive pulmonary disease, pulmonary hypertension, reactive airway disease, or chronic lung condition of any aetiology or who have experienced:<br>o Significant/severe wheeze in the past<br>o Respiratory symptoms including wheeze which has ever resulted in hospitalisation<br>o Known bronchial hyperreactivity to viruses<br>b) History of thromboembolic, cardiovascular or cerebrovascular disease<br>c) History or evidence of diabetes mellitus<br>d) Any concurrent serious illness including history of malignancy that could interfere with the aims of the study or a subject completing the study. Basal cell carcinoma within 5 years of treatment or with evidence of recurrence is also an exclusion<br>e) Migraine with associated neurological symptoms such as hemiplegia or vision loss. Cluster headache/migraine or prophylactic treatment for migraine<br>f) History or evidence of autoimmune disease or known immunodeficiency of any cause.<br>g) Other major disease that, in the opinion of the Investigator, could interfere with a subject completing the study and necessary investigations.<br>h) Immunosuppression of any type
2.	Any significant abnormality altering the anatomy or function of the nose or nasopharynx in a substantial way (including loss of or alterations in smell or taste), a clinically significant history of epistaxis (large nosebleeds) within the last 3 months, nasal or sinus surgery within 6 months of inoculation.
3.	Clinically active rhinitis (including hay fever) or history of moderate to severe rhinitis, or history of seasonal allergic rhinitis likely to be active at the time of inclusion into the study and/or requiring regular nasal corticosteroids on an at least weekly basis, within 30 days of admission to quarantine.

NO	STANDARD EXCLUSION CRITERIA
4.	History of anaphylaxis and/or a history of severe allergic reaction or significant intolerance to any food or drug, as assessed by the PI.
5.	History or presence of alcohol addiction, or excessive use of alcohol (average weekly intake in excess of 28 units alcohol; one unit being a half glass of beer, a small glass of wine or a measure of spirits), or use of drugs of abuse
6.	Psychiatric illness including subjects with a history of depression and/or anxiety with associated severe psychiatric comorbidities, for example psychosis. Specifically,<br>a) Subjects with history of anxiety-related symptoms of any severity within the last 2 years if the Generalized Anxiety Disorder-7 score is ≥4<br>b) Subjects with a history of depression of any severity within the last 2 years if the Patient Health Questionnaire-9 score is ≥4
7.	Subjects who have smoked ≥5 pack years at any time [5 pack years is equivalent to one pack of 20 cigarettes a day for 5 years]).<br>• Subjects who have smoked <5 pack years - at any time in the 3 months prior to admission to the quarantine unit they have used tobacco in any form (e.g., smoking or chewing) or other nicotine-containing products in any form (e.g., gum, patch) or electronic cigarettes.
8.	Family history of 1st degree relative aged 50 years or less with sudden cardiac or unexplained death
9.	Family History of Severe COVID or response to any other viral disease e.g. Guillain–Barré
**Measurements and investigations**	
10.	A total body weight of ≤ 50kg and a Body Mass Index (BMI) ≤18 kg/m2 and ≥28 kg/m2. The upper limit of BMI may be increased to ≤ 30kg/m2 at the PI's discretion, in the case of physically fit muscular individual
11.	Venous access deemed inadequate for the phlebotomy and cannulation demands of the study.
12.	Any clinically significant abnormal finding on screening biochemistry, haematology and microbiology blood tests or urinalysis i.e. grade 1 lab abnormalities or above (see Appendix 3 Toxicity Grading Scale for Laboratory AEs) apart from minor deviations which are clinically acceptable and approved by the Principal Investigator<br>a) Elevated random glucose and HbA1C

NO	STANDARD EXCLUSION CRITERIA
	b) Positive HIV, active/chronic hepatitis A, B or C test.<br>c) Confirmed positive test for drugs of abuse on admission and urinary cotinine at quarantine.
13.	A forced expiratory volume in 1 second (FEV1) and a forced vital capacity (FVC) <80% of predicted value calculated using ATS/ERS guidance (refer to section 5, respiratory samples)
14.	Twelve-lead ECG recording with clinically relevant abnormalities as judged by the study physician/PI.
15.	Echocardiogram outside normal parameters at baseline
**Recent respiratory infection**	
16.	History of, or currently active symptoms suggestive of upper or lower respiratory tract infection (including reduced sense of taste and smell, raised body temperature and/or persistent cough) within 6 weeks prior to viral challenge.
17.	Presence of cold-like symptoms and/or fever (defined as subject presenting with a temperature reading of >37.9ºC) on Day -2, Day -1 and/or pre-challenge on Day 0.
18.	Evidence of any respiratory viruses (on nasopharyngeal swab analysis) prior to challenge virus inoculation on admission to the quarantine unit. These include:<br>VIRUSES:<br>● Adenovirus<br>● Coronavirus HKU1<br>● Coronavirus NL63<br>● Coronavirus 229E<br>● Coronavirus OC43<br>● Human Metapneumovirus<br>● Human Rhinovirus/Enterovirus<br>● Influenza A<br>● Influenza A/H1<br>● Influenza A/H3<br>● Influenza A/H1-2009<br>● Influenza B

NO	STANDARD EXCLUSION CRITERIA
	● Parainfluenza Virus 1<br>● Parainfluenza Virus 2<br>● Parainfluenza Virus 3<br>● Parainfluenza Virus 4<br>● Respiratory Syncytial Virus<br><br>BACTERIA:<br>● Bordetella parapertussis<br>● Bordetella pertussis<br>● Chlamydia pneumoniae<br>● Mycoplasma pneumoniae
**Receipt of medications and interventions**	
19.	Evidence of a live vaccine within 60 days prior to the planned date of viral challenge, a non-live vaccine within 30 days prior to the planned date of viral challenge, or intention to receive any vaccination(s) before the day 28 follow-up visit. (NB. No travel restrictions applied after the Day 28 Follow-up visit).
20.	Receipt of blood or blood products, or loss (including blood donations) of 550 mL or more of blood during the 3 months prior to the planned date of viral challenge or planned during the 3 months after the final visit.
21.	Medications<br>a) Use of any medication or product (prescription or over-the-counter), for symptoms of hayfever, nasal congestion or respiratory tract infections or dermatitis/eczema including the use of regular nasal or medium-high potency dermal corticosteroids, antibiotics and First Defence™ (or generic equivalents) within 7 days prior to the planned date of viral challenge apart from those described in Table 7, Permitted Medication or agreed by the Principle Investigator<br>b) Receipt of any investigational drug within 3 months prior to the planned date of viral challenge.<br>c) Receipt of three or more investigational drugs within the previous 12 months prior to the planned date of viral challenge.<br>d) Prior inoculation with a virus from the same virus-family as the challenge virus.<br>e) Receipt of systemic (intravenous and/or oral) glucocorticoids or systemic antiviral drugs within 6 months prior to the planned date of viral challenge.

NO	STANDARD EXCLUSION CRITERIA
	f) Over the counter medications (e.g., paracetamol or ibuprofen) where the dose taken over the preceding 7 days prior to the planned date of viral challenge had exceeded the maximum permissible 24-hour dose (e.g., >4g per day of paracetamol over the preceding week).<br>g) Use or anticipated use within 7 days prior to the planned date of viral challenge and during the conduct of the study of concomitant medications (prescription and/or non-prescription), including vitamins or herbal and dietary supplements within the specified windows.<br>h) Chronically used medications, vitamins or dietary supplements, including any medication known to be a moderate/potent inducer or inhibitor of cytochrome P450 enzymes, within 21 days prior to the planned date of viral challenge.<br>i) Subjects who have received any systemic chemotherapy agent, immunoglobulins, or other cytotoxic or immunosuppressive drugs at any time.
22.	Prior participation in another human viral challenge study in the preceding 12 months taken from the date of viral challenge in the previous study to the date of expected viral challenge in this study.
23.	Any nasal sampling procedure in the 6 months before date of expected viral challenge in this study (excluding study tolerance test or routine tests for COVID-19)
**General**	
24.	Subject was mentally or legally incapacitated in the opinion of the Investigator.
25.	Females who:<br>a) Are breastfeeding within 6 months of study commencement, or<br>b) Had been pregnant within 6 months prior to the study, or<br>c) Had a positive pregnancy test at any point during screening or prior to inoculation with challenge virus
26.	Those in close domestic contact (i.e. sharing a household with, caring for, or daily face to face contact) with children under 2 years, the elderly (>65 years), immunosuppressed persons, or those with chronic respiratory disease
**Other**	
27.	Was employed or was a first-degree relative of anyone employed by the Sponsor, a participating clinical trial site, or any Contract Research Organisation involved in the study.
28.	Any other reason that the Investigator considered made the subject unsuitable to participate.
29.	Participants with no knowledge of their family history

Prior to enrolment, each potential participant deemed to be suitable for entry into the study (based on inclusion/exclusion criteria and their screening assessments) will undergo a personalised risk assessment using the QCOVID tool in combination with emergent results from individuals experimentally infected with SARS-CoV-2 in this study. The risk assessment will be reviewed by a member of the study medical team, documented and discussed with the participants before enrolment.

Personalised risk assessment using the QCOVID tool -

https://qcovid.org/PatientInformation/PatientInformation

3.10 Lifestyle Considerations

3.10.1 Meals and dietary restrictions

No dietary restrictions are required before or after dose administration.

3.10.2 Caffeine, alcohol and tobacco

During quarantine, participants must abstain from ingesting more than three cups of coffee, tea or other caffeine-containing products per day. Decaffeinated drinks will be made available.

Participants must not consume alcohol for 72 hours prior and during quarantine and for 72 hours prior to any clinic visits.

Participants must not smoke, use tobacco or any nicotine containing products for 3months prior to and during quarantine.

3.10.3 Activity

Participants will abstain from strenuous exercise that is unusual for them, for 48 hours prior and during quarantine and for 48 hours prior to each clinic visit (this is at the discretion of the Investigator).

3.11 Screen Failure

Screen failures are defined as participants who sign the screening study Informed Consent Form (ICF) but who are not subsequently enrolled in the study.

For individuals who do not meet the criteria for participation in this study (screen failure), the Investigator will decide whether the participant should be permanently excluded from the study or invited back for repeat assessments (i.e. repeat clinical laboratory test) if the initial screening assessments are still within the allowed screening windows, or rescreening for a later quarantine, as appropriate.

4. Study Intervention(s)

Study interventions administered to participants are described in Table 7.

4.1 Summary of Study Intervention(s) Administered

Table 7 - Study Interventions

**Intervention Name**	• SAR-COV-2	• Veklury® (Remdesivir)	• REGEN-COV<br>• (casirivimab with imdevimab)
**Type**	• Virus	• Drug (Pre-emptive treatment)	• Drug (Rescue treatment)
**Dose Formulation**	• Ampoule, Liquid	VEKLURY™ (Remdesivir) for injection, 100 mg, available as a sterile, preservative-free, white to off-white to yellow lyophilized powder	The solution for each vial should be clear to slightly opalescent, colourless to pale yellow
**Unit Dose Strength(s)**	• The titre of the undiluted Challenge Virus stock (Master Virus Bank) is determined in an infectivity assay and is reported in Tissue Culture Infective Dose units per mL (TCID50/mL). The inoculum virus vials are produced by dilution of the master virus bank to achieve targeted challenge doses, as follows:<br>• Dose level 1 ~ 10 $TCID_{50}$ (~10^1 $TCID_{50}$ dose)	Each 100 mg single-dose vial contains a sterile, preservative-free lyophilized powder that is to be reconstituted with 19 mL of Sterile Water for Injection and diluted into 0.9% sodium chloride prior to administration by intravenous infusion. Following reconstitution, each vial contains 100 mg/20 mL (5 mg/mL) of VEKLURY (Remdesivir) concentrated solution.	Casirivimab and imdevimab solutions must be diluted prior to administration.<br>Casirivimab with Imdevimab 1,200 mg Dose.<br>Further details are described in the pharmacy manual. recovery-regn-cov2-pharmacy-manual-v4-0-2020-11-04.pdf (recoverytrial.net)

	• Dose level 2 ~ 100 $TCID_{50}$ (~10^2 $TCID_{50}$ dose)<br>• Dose level 3 ~ 1,000 $TCID_{50}$ (~10^3 $TCID_{50}$ dose)<br>• Optional additional Dose levels (e.g. ~1 $TCID_{50}$, ~10,000 $TCID_{50}$, ~ 100,000 $TCID_{50}$,		
**Dosage Level(s)**	• Different doses of virus will be evaluated. Dose volume delivery method is provided in the Section 4.2.5.	• The recommended dosage for adults weighing at least 40 kg is a single loading dose of VEKLURY (Remdesivir) 200 mg on Day 1 followed by once-daily maintenance doses of VEKLURY (Remdesivir) 100 mg from Day 2. The treatment duration is 5 days.	• The dosage in adults is 600 mg of casirivimab and 600 mg of imdevimab administered together as a single intravenous infusion via pump or gravity. The duration of treatment is 1 day
**Route of Administration**	• Intranasal	• IV infusion	• IV infusion
**Use**	• Infectious challenge agent	• Pre-emptive antiviral treatment	• Rescue treatment
**IMP and NIMP**	• N/A	• NIMP	• NIMP
**Sourcing**	• Provided centrally by hVIVO	• Provided centrally by the Sponsor.	• Provided centrally by the Sponsor.
**Packaging and Labelling**	• Challenge Inoculum will be provided in single-dose vials. The details of the virus challenge agent will be provided in the Analytical Plan	• Packaging and Labelling of the study Intervention is described in the Investigational Medicinal Product Dossier 2.1.P.3 Manufacture [Remdesivir (GS-	• Packaging and Labelling of the study Intervention is described in the Investigational Medicinal Product Dossier.

		5734™) for Injection].	
**Current/Former Name(s) or Alias(es)**	• N/A	• Remdesivir ™, Veklury	• REGEN-COV (casirivimab with imdevimab)

Refer to Section 4 and the pharmacy manual for details regarding administration of the study intervention, and Section 4 of the analytical plan for further details of the virus inoculum.

4.2 SARS-CoV-2 Challenge Virus

4.2.1 Provenance of the wild type SARS-CoV-2 challenge virus

The SARS-COV-2 challenge virus strain was originally obtained from a nose/throat swab taken from a patient in the UK who developed respiratory symptoms consistent with COVID-19. The virus was isolated by inoculation with the clinical sample of a qualified cGMP Vero Cell line. A Seed Virus Stock was then generated by a further passage on the same cGMP Vero Cell line. The Zayed Centre for Research (ZCR) GMP manufacturing facility of Great Ormond Street Hospital (GOSH) subsequently used the Seed Virus Stock to manufacture the Challenge Virus in accordance with cGMP and produce a Challenge Virus Master Virus Bank.

Individual person inoculum vials were then produced in accordance with cGMP by GOSH by dilution of the cGMP MVB with cGMP sucrose diluent. Inoculum vials have been produced at a range of viral concentrations. The challenge virus has undergone extensive quality testing performed as part of the GMP manufacturing release processes according to pre-determined specifications (including identity, infectivity and contaminant / adventitious agent tests). The challenge virus will be stored in a secure –80°C freezer (normal temperature range –60°C to –90°C). This virus has not been used in previous human virus challenge studies.

4.2.2 Justification for treatment dose and route of administration

The starting SARS-CoV-2 dose (10^1 TCID50) was selected as a dose that is below the lowest reliably quantifiable amount of infectious virus. Refer to section 4 for more details on dose escalation.

4.2.3 Supply and accountability of challenge virus

The study site will establish a system for control of challenge virus in accordance with site SOPs and as detailed in the analytical plan. The PI will maintain accurate records of receipt and condition of all challenge virus inoculum stock used for challenge in accordance with the site SOPs, including details and dates of the batch and vial numbers, quantities dispensed and used in the study. Any departures from the protocol-dispensing regimen will be fully documented. Accountability records will be maintained as per the hVIVO site and hVIVO laboratory SOPs.

4.2.4 Storage of challenge virus

Stocks of challenge virus will be maintained as per the site analytical plan and SOPs and will be stored in a secure –80°C freezer (normal temperature range –60°C to –90°C). Vials will be thawed just before inoculum preparation. Once thawed, vials of stock virus will not to be re-used for human challenge studies. The residual of the inoculum in the vials used for challenging the subjects with the challenge virus will be frozen and stored in accordance with the analytical plan. All storage records were maintained as per the analytical plan.

4.2.5 Preparation and administration of challenge virus

All participants will be administered GMP-compliant SARS-CoV-2 at one of dose levels by intranasal drops on Day 0, starting at 10^1 TCID50 dose (see Section 6). In this exploratory dose optimisation study, increasing doses of wild-type SARS-COV-2 will be given via nasal administration to healthy volunteers. Up to three viral dose levels are anticipated to be needed, however higher or lower doses may be required, as appropriate:

- Dose level 1 ~ 10 TCID50 (~10^1 TCID50 dose)
- Dose level 2 ~ 100 TCID50 (~10^2 TCID50 dose)
- Dose level 3 ~ 1,000 TCID50 (~10^3 TCID50 dose)
- Optional additional Dose levels that may be evaluated
  - > 1,000 TCID50 (i.e. ~10,000 TCID50, ~ 100,000 TCID50,)
  - < 10 TCID50 (e.g. ~1 TCID50)

SARS-CoV-2 virus will be used for experimental infection of volunteers. On the day of inoculation (Day 0), aliquots of challenge virus will be removed from storage in the -80 ^{o}C freezer and transferred to the quarantine unit on dry ice.

A clinically trained staff member will inoculate each volunteer in the high containment room in which the volunteer will subsequently remain quarantined, with another staff member acting as scribe-/timekeeper/assistant. A sign will be placed on the door of the room to indicate that inoculation is taking place and to prevent accidental entry of others. Staff will wear full PPE (FFP3 mask or respirator; full waterproof gown; visor; gloves; and shoe covers) when in the participant's room during and at all times after inoculation.

The inoculum dose will be rapidly defrosted by warming in the hand and then placed on ice (if necessary). Subjects will then be inoculated with intra-nasal drops (100 µl/naris) with inoculum at a given dose divided equally between the two nostrils. Inoculations using intranasal drops will be done using a pipette with subjects' supine (face and torso facing up and remaining so for at least 20 minutes post inoculation). This will be done slowly with at least 30s intervals between each nostril (inoculation) to ensure maximum contact time between with the nasal and pharyngeal mucosa. Subjects will be asked not to swallow during the procedure to ensure maximal pharyngeal contact and not to shower or blow their nose for at least two hours. Following inoculation, advice regarding infection control, emergency call and hand hygiene will be given.

To quantify the amount of SARS-COV-2 inoculated into each volunteer within a cohort, a quantitative culture (infectivity assay) may be performed on an aliquot of the challenge inoculum removed before participant administration, stored on ice and transported immediately to the virology laboratory. Volunteer inoculations may also be back titrated using qRT-PCR. Where doses given that may not be quantifiable by cell culture given (e.g. 10^1 $TCID_{50}$) due to the limitations of the assay sensitivity, the genome copies may be used to confirm that the intended dose was given.

4.3 Antiviral Treatment (Remdesivir as Pre-emptive Medication)

Participants administered SARS-COV-2 may receive Remdesivir as an IV infusion for five days in a total volume of up to 250 mL 0.9% sodium chloride over 30 to 120 minutes. If pre-emptive treatment planned to be given, Remdesivir treatment will commence after either:

- confirmation of SARS-CoV-2 infection at two consecutive time points*, or
- immediately after symptoms, signs or investigations suggestive of COVID-19

*dosing will commence the following day of the second confirmed swab or confirmed symptoms, if this falls during the night

Details of Remdesivir dose preparation and administration will be described in the Pharmacy Manual. Please refer to the study pharmacy manual for instructions.

The timing and benefit of Remdesivir will be kept under constant review by the DSMB.

4.4 Dose Preparation

Remdesivir doses for each individual participant will be prepared by a pharmacist or designee (e.g. nurse) in the quarantine unit at the Royal Free Hospital. Dose preparation process will be described in the pharmacy manual and in accordance with the Veklury® (Remdesivir) Investigator's Brochure.

4.5 Early "Rescue" Therapy

Early "rescue" therapy, when backed by compelling evidence of efficacy and when available, is indicated for challenge model infections that demonstrate warning features beyond mild signs and symptoms that are confined to the upper respiratory tract. Indications to consider rescue therapy in those subjects confirmed to be infected include:

- Persistent tachypnoea – Respiratory Rate ≥ 21 for ≥ 8 hours
- Persistent Fever – Fever (≥ 37.9) from a time point 5 days post symptom onset and present for at least once each day for ≥ 72 hours
- Severe and persistent cough – Grade 3 (defined as "quite bothersome most or all of the time, and it stops me from participating in activities") reports of coughing via symptom diary cards that is largely persistent over 48 hours
- For CT changes related to SARS CoV-2 infection, anything more than mild changes (based on the British Society of Thoracic Imaging COVID reporting scale) represents a standalone trigger and mild changes in combination with other concerning clinical features could also be considered.
- Any event of confirmed hypoxia (≤ 94%, usually confirmed over a 1-hour period) should this occur without the above 'warnings'

Importantly CI/PI discretion will be used at all times in the decision to start Rescue Therapy. Biochemical markers (e.g. elevated CRP/D-dimer) will not be used in isolation, though a combination of factors outside of the triggers above could still lead to a decision to start therapy.

Once a decision to commence rescue therapy has been made by the CI/PI, the NHS Infectious Diseases team at the Royal Free Hospital will be made aware. The subject will remain within the quarantine unit to receive rescue therapy unless protocol stated criteria for transfer to NHS care are met.

"Rescue" treatment using Regeneron COV2

Participants administered SARS-COV-2 may receive REGEN-COV as a rescue therapy (when indicated) as a single IV infusion in a total volume of up to 250 mL 0.9% sodium chloride over 30 to 120 minutes.

Details of REGEN-COV dose preparation and administration will be described in the Pharmacy Manual. Please refer to the study pharmacy manual for instructions.

The benefit of REGEN-COV will be kept under review by the CI/PI, to decide whether or not it is appropriate to continue to use REGEN-COV, with discussion with the DSMB.

4.6 Dose Preparation

REGEN-COV doses for each individual participant will be prepared by a pharmacist or designee (e.g. nurse) in the quarantine unit at the Royal Free Hospital. Dose preparation process will be described in the pharmacy manual and in accordance with the Investigator's Brochure.

4.7 Handling, Storage, and Accountability

All study interventions:

1. The Principal Investigator or designee must confirm appropriate temperature conditions have been maintained during transit for all study intervention received and any discrepancies are reported and resolved before use of the study intervention
2. Only participants enrolled in the study may receive study intervention and only authorised site staff may supply or administer study intervention. All study intervention must be stored in a secure, environmentally controlled, and monitored (manual or automated) area in accordance with the labelled storage conditions with access limited to the Investigator and authorised site staff
3. The Principal Investigator, is responsible for study intervention accountability, reconciliation, and record maintenance (i.e., receipt, reconciliation, and final disposition records)
4. Disposal of used and unused Virus inoculum vials will be done in accordance with hVIVO's SOPs
5. Procedure for partly used or unused supplies of intervention treatment will be described in the pharmacy manual.

4.8 Randomisation and Blinding

Not applicable.

4.9 Study Intervention Compliance

Interruptions from the protocol-specified intervention plan require consultation between the investigator and the Sponsor and written documentation of the collaborative decision on participant management.

Any non-compliance or problems with the administration of the study intervention will be recorded in the participant's source notes and documented as a protocol deviation if appropriate.

4.10 Concomitant and Prior Therapy

Permitted Medication

- Subjects who were taking, wanted to take, or were required to take regular medication (whether prescribed or not) during their participation are excluded from this study with the exception of those therapies listed below or unless agreed with the PI. Use of all permitted therapies has to be documented and agreed at the screening visit.
- The use of concomitant medications other than those listed in Table 8 is prohibited unless approved by the PI, including over-the-counter or prescribed medications. Where there is uncertainty, the PI is encouraged to discuss the use of any concomitant medications with the CI/Chief Investigator before initiating therapy.
- The Investigator is to be informed as soon as possible about any medication taken by a subject from the time of screening until the completion of the follow-up visit on Day 28. Agreed concomitant medications taken during the quarantine phase will be stored, prescribed and administered in line with their label-specific requirements and full accountability will be maintained. Use of all concomitant medications will be recorded in the case report form (CRF). Concomitant medications include all prescription drugs, herbal preparations, over-the-counter medications, vitamins and minerals.

Table 8: Permitted medications and restrictions

Permitted medication	Restrictions
Oral, injected or implanted contraceptives or hormone replacement therapies	Recommended dosing
Paracetamol	Maximum daily dose of 4 g from 7 days before Day -1 and throughout the duration of the study
Mild potency topical steroids	Recommended dosing
Over-the-counter creams and topical	Recommended dosing

treatments	
Prescription and non-prescription medications, including vitamins or herbal and dietary supplements, not listed in prohibited medications are subject to approval by the PI.	

Prohibited Medication

All medications, other than those noted above are to be stopped before the planned date of viral challenge unless in the opinion of the Investigator and/or Sponsor's Medical Monitor, the medication will not interfere with the study procedures or compromise subject safety. Certain medications requiring a specific washout period before a subject is eligible to enter the study; details are provided in Table 9. These medications are prohibited until day 28.

Table 9 Prohibited Medications

**Prohibited medication**	**Washout required**
Systemic corticosteroid (oral and parenteral) therapy	6 months before the planned date of viral challenge
Systemic (oral and parenteral) antiviral drugs	6 months before the planned date of viral challenge
Vaccinations	30 days (non-live vaccine) or 60 days (live vaccine) before the planned date of the study depending on the vaccine
Short and long-acting anti-histamines	7 days before study-specific screening
Any medication or product (prescription or over-the-counter), for symptoms of nasal congestion or respiratory tract infections including nasal steroids	7 days before the planned date of viral challenge
Herbal supplements	7 days before the planned date of viral challenge
Chronically used medications, vitamins or dietary supplements, including any medication known to be an inducer or inhibitor of cytochrome P450 .enzymes	21 days before the planned date of viral challenge

4.11 Dose Escalation, De-escalation and Confirmation

The study will involve dose escalation of SARS-CoV-2 to demonstrate safety and identify an optimum dose in participants with no evidence of previous SARS-CoV-2 infection including no detectable antibodies. Groups of up to 20 individuals will be challenged at a time as shown in Figure 4:

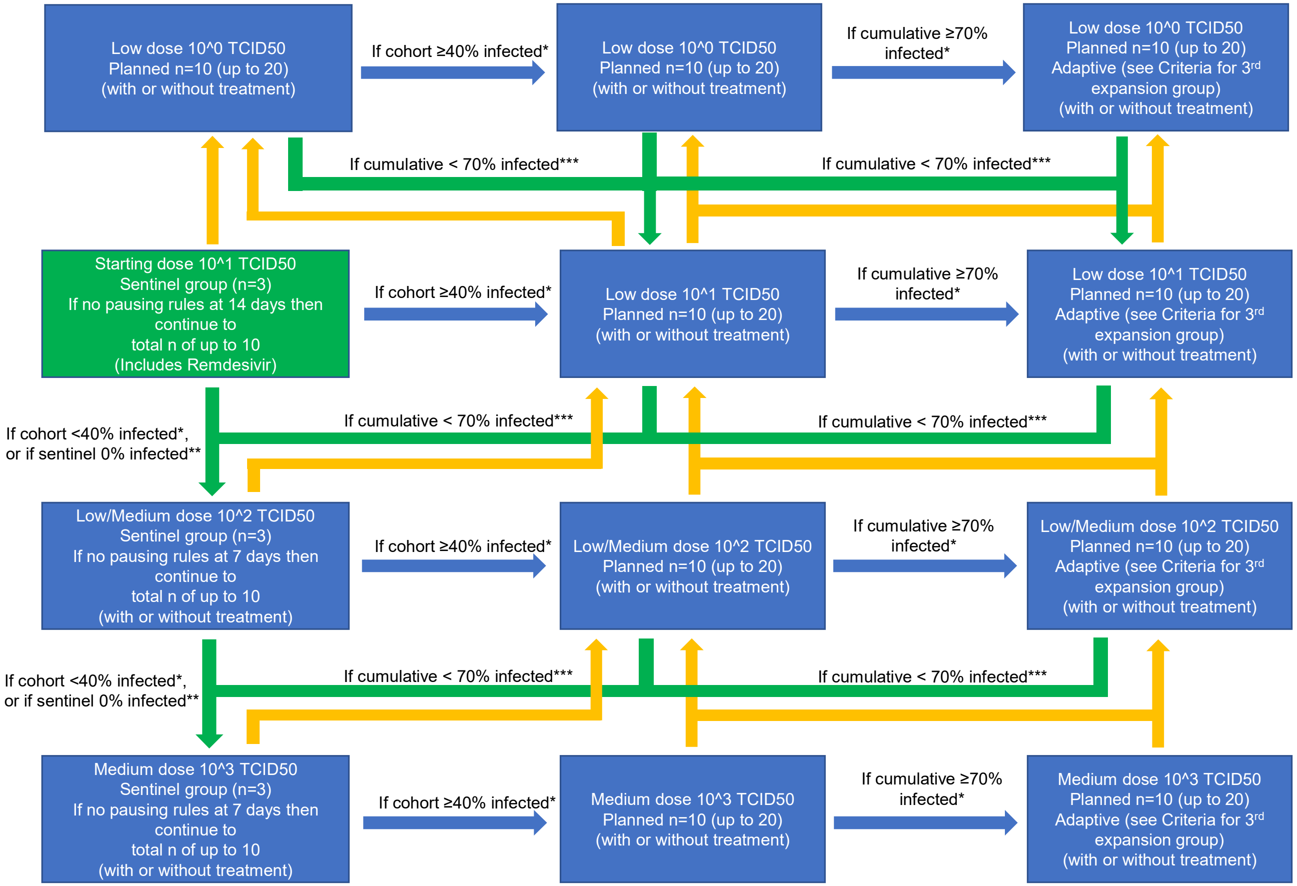

Figure 4 Group and Dose Escalation Scheme

*Next inoculation ~1 week from last participant discharge (minimum 2 weeks of data), if agreed by DSMB, unless symptoms or investigations of concern by CI/PI.
**If no infections in sentinel participants, progress to next dose level (Low dose requires a minimum of 14 days data, higher doses require a minimum of 7 days data).

***If cumulative infection rate of all participants challenged at that dose level is <70%, the CI/PI may choose to progress to the next dose level ~1 week from last participant discharge (minimum 2 weeks of data), if agreed by DSMB, unless symptoms or investigations of concern by CI/PI.

Confirmation (**blue arrow**) of the same dose will occur if the following criteria are met (and in agreement with the DSMB):

- *Safety criteria*:
  - No safety concerns identified at dose level
  - Removal/addition of Remdesivir treatment in expanded cohorts will be decided upon after data review, and in agreement with the DSMB
  - Additional expansion cohorts may be performed.
- *Infectivity/disease criteria*: If the infection criteria **is** met (i.e. ≥40% or ≥70%, as appropriate)*,* **and**
  - <2 infected participants present with any grade 3 symptom of wheeze, shortness of breath, or chest tightness* **and**
  - <2 infected participants have grade 3 physician findings (DPE signs) of abnormal breath sounds (new wheezing, râles, rhonchi, other)

Escalation (**green arrow**) may be performed if **both** of the following criteria are met (and in agreement with the DSMB):

- *Safety criteria:* No safety concerns identified at dose level
- *Infectivity/disease criteria:* If the infection criteria **is not** met (i.e. <40% or <70% as appropriate)*,* **and**
  - <2 infected participants present with any grade 3 symptom of wheeze, shortness of breath, or chest tightness* **and**
  - <2 infected participants have grade 3 physician findings (DPE signs) of abnormal breath sounds (new wheezing, râles, rhonchi, other)

De-escalation (**orange arrow**) may be performed if **either** of the following criteria are met (and in agreement with the DSMB):

- *Safety criteria*:
  - Safety concerns are identified at the dose level, but that do not fulfil study stopping criteria
  - Removal/addition of Remdesivir treatment in expanded cohorts will be decided upon after data review, and in agreement with the DSMB
- *Infectivity/disease criteria*: no infectivity requirement, and
  - ≥2 infected participants present with grade 3 symptoms of wheeze, shortness of breath, or chest tightness * **or**
  - ≥ 2 infected participants have grade 3 physician findings (DPE signs) of abnormal breath sounds (new wheezing, râles, rhonchi, other)

Dose escalation will proceed as follows:

- The starting dose (10^1 $TCID_{50}$) was selected as a dose below the lowest reliably quantifiable infectious amount of virus. At this lowest dose, three sentinel individuals will be challenged. If none of the first three sentinel individuals develop infection after 14 days and no pausing rules are met (based on severity and frequency of adverse events, see Group Safety Holding Rules), the dose will be escalated to 10^2 $TCID_{50}$. If any of the first 3 participants develop infection after 14 days and no pausing rules are met, the remaining 7 participants in the group will be challenged.
- If any of the first 10 participants become infected, the next group of participants will be challenged at the earliest 7 days after the last participant was discharged (i.e. minimum of 3 weeks between viral inoculation).
- If the number of infected participants in the first group do not meet the pre-defined attack rate threshold*, the next group of participants will be inoculated with a higher dose.
- With each subsequent higher dose level, three sentinel individuals will be challenged. After 14 days, if no pausing rules are met (based on severity and frequency of adverse events, see Appendix 4: Adverse Events: Definitions and Procedures for Recording, Evaluating, Follow-up, and Reporting) the next 7 participants in the group can be challenged.
- If none of the first 10 participants at a dose level become infected by qPCR criteria, the next group will be challenged with the higher dose once all participants in the first group are discharged from quarantine (i.e. minimum of 2 weeks between viral inoculation).
- If any of the first 10 participants at a dosing level become infected, the next group will be challenged at the earliest 7 days after the last participant planned discharge (i.e. minimum of 3 weeks between viral inoculation).
- If the number of infected participants in the first group does not meet the pre-defined attack rate threshold*, the next group may be inoculated with a higher dose.
- If the highest dose of 10^3 $TCID_{50}$ is reached and the number of infected participants in the first group does not meet the pre-defined attack rate threshold* and no pausing rules are met, further dose escalations to 10^4 and 10^5 $TCID_{50}$ may take place.
- If the number of infected participants in the first group do meet the pre-defined attack rate threshold*, a second group of 10 participants will be challenged with the same dose level (i.e. resulting in 20 participants challenged at that dose level) to increase confidence for the attack rate at that dose level. A third group of 10 participants may be challenged if review of clinical and virological outcome data suggest that a larger sample size is needed to establish confidence in the attack rate using the same experimental conditions (see Statistics and Data Analysis).

If following dose escalation, review of clinical and virological outcome data suggest a risk of participants being exposed to excess risk (due to extremely high attack rates or high viral loads), the next group will be challenged with a lower dose and confidence in the attack rate of that lower dose will be increased (see Figure 4) Note: Dose escalations, de-escalations and confirmation group decisions will be made at the discretion of the Investigators with the advice of the DSMB. Decisions will be based not only on meeting the attack rate threshold but also

take into account the quantity of viral load, symptomatology and other clinical factors to identify the dose that is not only safe but also most appropriate for future use of the model for vaccine and therapeutics testing. Therefore, the attack rate thresholds defined above will not be absolute criteria for group progression but will be considered together with these other data. For example, if at a given dose the attack rate does not reach the pre-determined threshold but is associated with high viral loads in most group participants (such as a peak viral load of $\geq 10^7$ copies/mL), may be decided that a further group at the same dose is challenged rather than escalating to a higher dose level. Conversely, if an attack rate threshold is reached but the peak viral load by qPCR is low in all group participants (e.g. $<10^6$ copies/mL) and therefore unlikely to be culturable), dose escalation may still take place if deemed to be safe. Dose escalation decisions will be documented on site and entered into the s-cubed database who will provide oversight under their remit as database vendors.

Participant Withdrawal (Early Discontinuation of Quarantine only)

A participant may elect to leave quarantine before discharge criteria are met without withdrawing their consent to continue participating in the study. Wherever possible, the tests and evaluations listed for the Early Withdrawal Visit (EWV) should be carried out prior to the participant leaving the quarantine unit, and the participant should attend planned follow-up visits. It is likely that some of the assessments required as part of the EWV will already have been performed for the study day as per the SoA, in this case the completed assessments will not be repeated on the same day unless clinically indicated and the participant agrees.

Participants will be counselled that early withdrawal from the viral challenge phase of the study is strongly discouraged, as it may pose a risk both to the participant and his/her contacts. In the event of a participant insisting on early withdrawal during the quarantine period, the participant will be encouraged to stay in the quarantine unit and would be advised of the potential risks of carrying SARS-COV-2 infection into the community, and to vulnerable groups in particular. However, if withdrawal occurs after virus inoculation, every effort will be made to manage their withdrawal and do the following:

- The participant will be counselled about the risk of onward transmission of SARS-CoV-2 and the legal requirement to self-isolate according to PHE and government rules at the time
- The participant will be counselled about any risks due to the withdrawal and specifically any risks due to missed safety monitoring that cannot be performed at home
- The participant will be reminded about infection control procedures and receive re-training if necessary, in handwashing and isolation rules
- The participant will be informed they will not receive rescue therapy and the potential consequences
- The participant will be transported home in private transport with appropriate PPE, and in accordance with national guidance
- Daily follow-up calls will be made to check on participant’s health until the medical discharge, in line with their wishes.

- Once the quarantine discharge criteria are met, the participant will be informed they can de-isolate. Where it is not possible to obtain further virology swabs following departure from the quarantine unit, de-isolation will be advised according to government guidelines.

Participant Withdrawal

A participant may withdraw their consent to participate in the study at any time and for any reason, without prejudice to his/her future medical care. Participants may decline to give a reason for their withdrawal. If a participant withdraws from the study during the quarantine phase, an EWV will be completed, where possible, prior to the participant leaving the quarantine unit. It is likely that some of the assessments required as part of the EWV will already have been performed for the study day as per the SoA, in this case the completed assessments will not be repeated on the same day unless clinically indicated and the participant agrees.

Participants will be counselled that early withdrawal from the viral challenge phase of the study is strongly discouraged, as it may pose a risk both to the participant and his/her contacts. In the event of a participant insisting on early withdrawal during the quarantine period, the participant will be encouraged to stay in the quarantine unit and would be advised of the potential risks of carrying SARS-COV-2 infection into the community, and to vulnerable groups in particular. However, if withdrawal occurs after virus inoculation, every effort will be made to manage their withdrawal and do the following:

- The participant will be counselled about the risk of onward transmission of SARS-CoV-2 and the legal requirement to self-isolate according to PHE and government rules at the time
- The participant will be counselled about any risks due to the withdrawal and specifically any risks due to missed safety monitoring that cannot be performed at home
- The participant will be reminded about infection control procedures and receive re-training if necessary, in handwashing and isolation rules
- The participant will be informed they will not receive rescue therapy and the potential consequences.
- The participant will be transported home in private transport with appropriate PPE, and in accordance with national guidance
- The participant will be reminded of how to seek medical help from their GP or hospital if they feel more unwell

The PI may withdraw a participant if, in their clinical judgement, it is in the best interest of the participant or if the participant cannot comply with the protocol. Additionally, Participants can be withdrawn from the study for the reasons listed below if the Investigator feels this is necessary.

- Non-compliance with the study requirements and restrictions.
- Clinically significant abnormal laboratory findings, which in the opinion of the Investigator(s) and/or Sponsor, precludes further participation in the study.
- Development of inter-current illness which, in the opinion of the Investigator would compromise the health of the participant or the study objectives.

- The Investigator's decision that withdrawal from further participation would be in the participant's best interest.
- Termination of the study at the discretion of the Investigator(s) or Sponsor for safety, behavioural, or administrative reasons.
- Any intervention (Virus) related SAEs.
- The participant becomes pregnant.
- The wish of the participant.

The sponsor should be notified of all study withdrawals in a timely manner, and in cases where the withdrawal is due to a medical reason the participant would be referred to his/her GP.

If the participant withdraws consent for future disclosure of information, the Sponsor may retain and continue to use any data collected before such a withdrawal of consent.

If a participant withdraws from the study, he/she may request destruction of any samples taken and not tested, and the Investigator must document this in the site study records.

In the event that the participant loses capacity during the course of the study, they will be withdrawn from the study.

Criteria for Clinical Escalation of Participants

Participants will be closely monitored throughout by the study medical team.

Any illness recorded from the following list will lead to provision of immediate medical treatment, if necessary, followed by discussion with the CI/PI and possible referral for assessment by the RFH Patient Assessment and Response Team (PARRT) as agreed with the RFH and documented in the study specific Standard Operating Procedure (SOP).

- **Cardiovascular compromise**, such as -
- Sustained elevated heart rate >120bpm AND sustained low blood pressure SBP<100 for greater will than 30 minutes
- **Respiratory compromise**, such as
- Sustained elevated respiratory rate >30/min AND sustained low blood oxygen SaO2<94% for greater than 30 minutes
- Decrease in lung function of ≥15% in FEV1 (highest value) below the baseline value (Day 0, pre-inoculation) on consecutive readings over 30 minutes
- Fever (grade 2 or higher) for >48 hours
- Evidence of pneumonia on clinical examination
- **New ECG abnormalities** (compared to baseline), such as
- Confirmed Fridericia-corrected QT (QTcF) >500 msec; confirmed increase of QTcF >60 msec above baseline value
- Deranged and progressively worsening laboratory safety tests

- Any symptoms, signs or investigation results that are deemed to be of clinical concern by the CI / PI

A complete medical history will be provided to the receiving physician including the possibility of SARS-CoV-2 infection. If following assessment by the receiving clinician the participant is transferred for further care in the emergency department or in-patient ward, infection control measures, transfer procedures and further medical or surgical management will be according to local Royal Free Hospital SOPs. Hospitalisation of any participant for the above reasons will lead to immediate suspension of further inoculations.

In the event of a cardiac arrest, the hospital crash team will be summoned to respond.

The DSMB will be convened to assess the clinical evidence in order to determine whether the study may proceed (see Data Safety Monitoring Board).

Wherever possible, safety assessments and Follow Up Visits for participants who enter the NHS pathway, will continue, after their discharge from the NHS until completion of the study.

Lost to Follow up

A participant will be considered lost to follow-up if he or she repeatedly fails to return for scheduled visits and is unable to be contacted by the study site, in accordance with hVIVO SOP.

The following actions must be taken if a participant fails to return to the clinic for a required study visit:

The site must attempt to contact the participant and reschedule the missed visit as soon as possible and counsel the participant on the importance of maintaining the assigned visit schedule and ascertain whether or not the participant wishes to and/or should continue in the study.

Before a participant is deemed lost to follow up, the investigator or designee must make every effort to regain contact with the participant (where possible, 3 telephone calls and, if necessary, a follow-up letter to the participant's last known mailing address or local equivalent methods). These contact attempts should be documented in the participant's medical record.

Should the participant continue to be unreachable, he/she will be considered to have withdrawn from the study.

Participant Discontinuation of Study Intervention Therapy (Pre-emptive therapy / Rescue therapy)

Participants will be discontinued from study intervention therapy for the reasons listed below and at the discretion of the Principal Investigator. Although Participants will not receive any further intervention therapy, they will continue to be followed for safety and monitoring as per the SoA, including any additional unscheduled assessments as required for safety reasons.

- Any intervention therapy related SAEs.
- Clinically significant abnormal laboratory findings, which in the opinion of the Investigator(s) and/or Sponsor, precludes further receipt of study intervention therapy.

- The Investigator's decision that withdrawal from further intervention receipt would be in the participant's best interest.
- Anaphylactic reaction following intervention therapy receipt. The wish of the participant.

Participants who are discontinued from the study intervention therapy will complete their Quarantine stay and be required to attend their Follow Up Visits, with assessments as detailed in the SoA.

4.12 Temporary Discontinuation/Temporary Delay in Enrolment

At the first study visit, if a participant is found to be ineligible due to transient circumstances (such as acute disease and/or fever), the inoculation will be postponed until transient circumstances have been resolved and the participant will be re-invited to a later quarantine group within the allowed time window.

4.12 Participant Replacement Strategy

Participants who withdraw or are discontinued from the study may be replaced in order to achieve the planned evaluable number of participants as follows, if deemed appropriate by the PI and with the approval of the CI/Sponsor:

Withdrawals or early discontinuation prior to virus inoculation may be replaced.

4.13 Stopping Rules

The CI, PI and the DSMB will perform safety reviews on available clinical and virology data during the quarantine period, as appropriate.

The Clinical scenario relating to the incidence of SAEs/AEs during the study and the procedures that should be performed in this instance are presented in Table 10.

Table 10 Study Stopping Rules

Status	Criterion	Procedure
1	Virus-related SAE or Virus-related AEs of clinical concern have been reported following Human Viral Challenge.	If such a status occurs at any point during the study, then the CI, PI and the DSMB will review the data and make a decision.<br><br>Further administration of the virus will be paused. The CI, PI and the DSMB will review the data and make a decision on whether it is appropriate to recommence inoculation (via a substantial amendment, if indicated) or terminate the study.

In any event, participant follow-up should continue until resolution or stabilisation of AEs and final follow-up on Day 360 (± 14 days).

Page Intentionally Blank.

5. Study Assessments and Procedures

Study procedures and their timing, including follow-up period at the Queen Mary BioEnterprise Centre (QMB) (Day 28-day – Day 360) are summarised in the SoA.

Protocol waivers or exemptions are not allowed.

Adherence to the study design requirements, including those specified in the SoA, is essential and required for study conduct.

All screening evaluations must be completed and reviewed at D-2/-1 to confirm potential participants meet all eligibility criteria. A screening log will be maintained to record details of all participants screened and to document eligibility or record the reasons for screening failure, as applicable.

For all study assessments, the value obtained nearest to inoculation will be used as the baseline measure for assessments.

Procedures conducted as part of the Screening Protocol and obtained before signing of the study specific Informed Consent Form (ICF) may be utilised for screening or baseline purposes provided the procedures met the protocol-specified criteria and were performed within the time frame defined in the SoA.

Where applicable, unless otherwise stated, normal ranges will be filed in the Trial Master File (TMF).

5.1 Medical and Medication History

Medical and medication histories including any allergies will be recorded at screening, including, but not limited to, detailed histories on allergies [e.g. rhinitis, dermatitis, food, aspirin/non-steroidal anti-inflammatory drugs (NSAIDs) and asthma]. Medical history from the participant's GP will be obtained at screening and reviewed for eligibility.

5.2 Demographics

Demographic data will be recorded at screening and repeated at D -2/-1 in accordance with the SoA.

5.3 Height, Weight and Body Mass Index (BMI)

Height and weight measurements will be recorded in compliance with hVIVO's standard procedures.

BMI will be calculated as: BMI (kg/m2) = $\frac{\text{Weight (kg)}}{\text{Height (m)2}}$

5.4 Alcohol Breath Testing

Breath alcohol testing will be conducted to determine compliance with the study alcohol restrictions in accordance with hVIVO SOP. Additional tests may be conducted for assessing eligibility at the discretion of the investigator. Results will be recorded in the source documents.

5.5 Physical Examinations

A complete physical examination will include a full systemic assessment and will be conducted by an investigator or medically qualified designee.

Directed physical examinations will be conducted as deemed appropriate by the CI/PI and will include examination of the ears, nose, throat and chest (via stethoscope).

Assessment and grading of any upper respiratory tract (URT) (nasal discharge, otitis, pharyngitis, sinus tenderness) and lower respiratory tract (LRT) symptoms (abnormal breath sounds externally [e.g. stridor] and on chest auscultation [wheezing or rhonchi, crepitations] will be performed. Physician-reported assessments of viral challenge related illness will be graded in accordance with their intensity and documented in the source data.

Following viral challenge, URT and LRT symptoms (as described above) will be expected and presumed to represent virus infection consequent to viral challenge and will not be additionally captured as AEs unless they meet the definition of an AE, and are deemed to be clinically significant (in the opinion of the Investigator) to be classed as AEs.

Following viral challenge all unexpected (in the opinion of the Investigator) directed physical examination findings will be captured as AEs, along with all other occurrences that meet the criteria for an AE.

5.6 Vital Signs

Subjects will be rested in a supine position for a minimum of 5 minutes before vital signs assessments. During vital signs assessments, subjects will be rested in a quiet setting without distractions (e.g., television, mobile phones, computers).

Vital signs assessments will be recorded as follows:

- Heart rate (HR) will be recorded in beats per minute.
- Respiratory rate (RR): respirations will be counted and recorded as breaths per minute.
- BP: systolic BP and diastolic BP will be measured in millimetres of mercury (mmHg); measurements will be made supine. Where possible, the same arm will be used for all measurements.
- Peripheral arterial oxygen saturation (SpO2%) will be assessed using pulse oximetry.

In the event of a participant having an unexpected abnormal or out of normal range result, the assessment may be repeated after at least 2 minutes to exclude a technical fault and confirm

the original reading. The assessment may then be repeated at the PI's discretion and in accordance with SOPs.

Study specific normal ranges are provided in Section 8 a result is out of the normal range and meets the criteria for an AE, the severity of the AE will be guided by the Division of Microbiology and Infectious Diseases (DMID) Adult Toxicity Table November 2007.

Deterioration in a vital sign (compared to baseline) should only be reported as an AE if the deterioration fulfils the criteria for an AE. If deterioration in a vital sign is associated with clinical signs and symptoms, the sign or symptom will be reported as an AE and the associated vital sign will be considered as additional information.

5.7 Temperature

The study specific normal range for tympanic temperature is detailed in Appendix 5.The severity of out of normal range values will be assigned using the DMID toxicity scale as a guide.

Temperature may be more frequently monitored in quarantine if deemed necessary by the Investigator.

Following viral challenge, pyrexia will be expected and presumed to represent virus infection consequent to viral challenge and will not be additionally captured as an AE unless it meets the definition of an AE and is deemed to be clinically significant (in the opinion of the Investigator) to be classed as an AE.

Following viral challenge all unexpected (in the opinion of the Investigator) pyrexia will be captured as an AE, along with all other occurrences that meet the criteria for an AE.

Febrile illness (FI) is defined as any occurrence of temperature ≥ 37.9 ^{o}C.

5.8 Radiology

Chest X-rays and CT scans will be carried out in the hospital radiology department associated with the quarantine unit, according to local NHS SOPs. A chest x-ray will be performed upon admission to quarantine. Where possible, this should be acquired with the patient erect, taking a full inspiration and using a Posterior-Anterior (PA) projection. Where an admitted volunteer was unable to be inoculated in a quarantine and asked to return for a subsequent quarantine, a repeat X-ray may not be required for subsequent admissions, unless requested by the CI/PI.

A CT of the chest will be acquired in all participants at either Day 5 or 6. The CT scan will be repeated on Day 10 or 11 in those participants with PCR-confirmed infection or symptoms consistent with COVID-19. An unenhanced volumetric CT scan will be performed. The patient will be positioned supine with their arms above their head and the scan performed with the patient at held full inspiration. The scan will be acquired from the lung apices to the costophrenic sulci to obtain full coverage of the lung parenchyma using a 0.625mm section thickness and reconstructed using a lung algorithm. The radiation dose from the CT scan makes it difficult to

justify performing an additional routine CT chest in the screening phase of the study, particularly given that the likelihood of finding abnormalities prior to inoculation is very low.

5.9 Echocardiogram

Transthoracic echocardiograms will be performed by a trained echocardiographer on admission to the quarantine unit before virus inoculation according to local NHS SOPs.

5.10 Electrocardiogram

Study specific normal ranges are provided in Appendix 5.

Twelve-lead ECGs will be obtained to evaluate the electrical activity of the heart, In accordance with hVIVO SOP. ECGs will be read on site by an appropriately qualified Investigator. Wherever possible the same Investigator will review subsequent ECGs from the same participant for the assessment of any change from baseline.

Any changes from baseline during the study will be assessed for their clinical significance. Clinically significant changes will be reported as AEs. The PI or delegate will assess non-clinically significant changes to determine whether they should be recorded.

5.11 Lung Function

5.11.1 Spirometry

Spirometry will be performed according to hVIVO's procedures. Height at screening will be used as the baseline measurement for all spirometry assessments.

Spirometry should meet the ATS/ERS guidelines criteria (Miller et al., 2005). For FEV_1 and FVC, the highest value from a minimum of 3 technically satisfactory attempts will be considered. For FEV_1 and FVC the highest and the second highest value should not exceed more than 150 mL or 5% (whichever is greater). If the difference is larger, up to 8 technically acceptable measurements will be made with repeatability assessed after each additional attempt. If after 8 technically acceptable attempts the difference remains greater than 150 mL or 5% (whichever is greater) the highest values will be reported, and an operator comment will be made to the source data. FEV_1 and FVC will be assessed and reported as the highest values regardless of curve.

Predicted values will be calculated according to the formula of the European Coal and Steel Community (ECCS).

FEF25-75 can be collected by hVIVO on a study-by-study basis as an exploratory endpoint governed by PI/sub-investigator discretion. This would not be used for eligibility assessment. FEF25-75 will be assessed from admission to quarantine discharge. As the variability of FEF-25-75, measured as coefficient of variation (CV), has been shown to be 20-30% for within test measurements on the same individual compared to 10-15% for FEV1, FVC etc as well as the

fact that FEF25-75 has been reported not to influence clinical decision making hVIVO will collect FEF-25-75 as an exploratory endpoint only (Quanjer et al., 2014; Stanojevic et al., 2008)

Spirometry may be repeated at any time in the event of respiratory signs or symptoms (repeated coughing, bradypnoea, tachypnoea, rales and rhonchi) or respiratory difficulties.

5.11.2 Forced oscillation technique (FOT)

Forced oscillation technique (FOT) is a versatile, non-invasive assessment of respiratory mechanics based on tidal breathing analysis. Airwaves of multiple frequencies (typically 5 to 30 Hz) are applied to the respiratory tract during relaxed breathing. The test involves participants breathing through a breathing handle in a seated position, wearing a nose-clip, with cheeks supported. Breathing is recorded for approximately 1 minute. During this time the instantaneous pressure-flow relationship, measured at the mouth at varying frequencies, are analysed in order to measure Respiratory Impedance (Zrs). Zrs is made up of an in-phase, termed resistance (Rrs) and out-phase, referred to as reactance (Xrs). Resistance at 5Hz penetrates the periphery of the lung, providing information of the entire respiratory tract. Resistance > 20Hz represents respiratory resistance of the proximal airways. Distal lung resistance can be calculated by subtracting total airway resistance from proximal airway resistance (R5-R20). Xrs comprises of 2 elements that are dependent upon frequency. At higher frequencies (>20 Hz) Xrs is positive in sign and represents the inertive force needed to move air in the conducting airways. At low frequencies of 5 Hz (X5) is negative in sign and indicates the capacitance of the lung periphery. A minimum of 3 tests will be performed and the mean values reported. Testing will take approximately 5 minutes.

5.12 Patient Health Questionnaire (PHQ-9) and Generalised Anxiety Disorder (GAD-7) Questionnaire

PHQ-9 and GAD-7 questionnaire will be used at the discretion of the investigator at screening and/or quarantine admission to assess participants' eligibility in terms of ability to tolerate isolation in the quarantine unit.

5.13 Participant Symptom Diary Card – Clinical scores

Participants will report and assess the severity of any challenge virus-related signs and symptoms three times/day during quarantine, at the same time each day (± 1 hour), using the Symptom Diary Card.

The following symptoms in the symptom questionnaire will be graded on a scale of 0-3

- grade 0: No symptoms;
- grade 1: just noticeable;
- grade 2: clearly bothersome from time to time but does not interfere with me doing my normal daily activities;
- grade 3: Quite bothersome most or all of the time, and it stops me participating in activities

- grade 4: Symptoms at rest. (Shortness of Breath and Wheeze only)

Table 11 List of symptoms recorded by subjects

Runny Nose	Cough	Chilliness/Feverishness
Stuffy Nose	Chest tightness	Dizziness
Sneezing	Shortness of Breath	Rashes
Sore Throat	Wheeze	Blisters
Hoarse voice	Malaise/Tiredness	Diarrhoea
Eye Soreness	Headache	
Earache	Muscle and/or Joint ache	

Additional to the categorical symptom diary card, a Visual Analogue Scale diary card using a 100mm scale, with the same symptoms, will be completed by the participants.

Once a day, (in the evening, approx. every 24 hours) participants will also be asked to confirm (Yes/No) if they have experienced any ‘Confusion, disorientation or drowsiness’ or any ‘Loss of appetite’ in the last day (24 hours).

Participant cold perception questions

Two additional cold-related questions will also be answered by the participant each morning. The first question asks whether the participant’s perception of whether they have a cold or not, the second asks the participant’s perception of improvement/worsening of the cold.

i. **Do you have a cold:** Yes/No

If the participant selects Yes to having a cold, then the second 7-point Likert scale “global change since yesterday” question is completed by the participant, as below.

ii. **Compared to yesterday**, I feel that my cold is:
    - Very much better
    - Somewhat better
    - A little better
    - The same
    - A little worse
    - Somewhat worse
    - Very much worse

5.14 University of Pennsylvania Smell Identification Test (UPSIT)

UPSIT is a well-validated and reliable test (test-retest r = 0.94) that employs microencapsulated "scratch and sniff" odorants. It is provided as booklets containing a series of cards that the participants scratch and smell then asked to identify the odours, which have been previously validated in an English population. The test provides an index of absolute dysfunction (ie, anosmia, severe microsmia, moderate microsmia, mild microsmia, normosmia, factitious), as well as relative dysfunction based upon age and gender-adjusted normative percentile ranks. The total number of odorant stimuli out of 40 that is correctly identified serves as the test measure. Scores on this test correlate well with other types of olfactory tests, including threshold tests. The UPSIT is designed to be self-administered after explanation of the test by study staff and will be performed once before virus inoculation and then at least every third day starting from Day 1, though the test can be conducted more frequently at the discretion of the PI/study physician.

5.14 Cognitive Testing

To assess changes in cognition associated with infection, a computerised system for repeated assessment of cognitive function will be used. This has been developed by Dr Adam Hampshire (Imperial College London) and has been successfully used in healthy individuals and clinical populations inclusive of traumatic brain injury. Participants will be provided with a study tablet computer assigned to them for from the time of their admission to the quarantine unit until discharge that will contain a pre-loaded app (CogAsses) and factory-provided software only. This app will provide a brief battery of computerised tasks and questionnaires to track speed of information processing, memory, attention, executive function and sleep. The battery of tests will last for ~20-30 minutes. Participants will be asked to complete the cognitive assessment every day around the same time of day during their admission within the quarantine unit. In addition, a standardised questionnaire about the quality of sleep from the preceding night will be included in the app. The cognitive tasks included within this battery of tests are:

1. Motor Control
2. Object memory-Immediate recall
3. Simple Reaction Time
4. Choice reaction time
5. 2D Manipulations
6. Allocentric towers
7. Spatial span
8. Target detection
9. Tower of London
10. Verbal analogies

11. Object memory-Delayed recall

Participants will also be invited to complete this assessment battery at each follow-up visit. All data from the cognitive assessments will be collected on a remote web server. The servers are behind a firewall in a secure cloud computing facility. These measures represent a high-level of data security that are the standard for any website. Remote storage of performance data will be in a pseudo-anonymised format and will be linked across tasks using anonymised user identifier codes. Identifiable information will be stored in an encrypted database 'key' that is separate from the test scores.

5.15 Urinalysis

Clinical urine safety analysis will be undertaken using commercially available urine test strips that provide an instant result and will be documented in the source data.

Urinalysis will be performed to evaluate the parameters described in Appendix 2.

If the dipstick yields abnormal results, a urine sample may be sent for microscopy, culture and sensitivity (MCS), at the Investigator's discretion. MCS will include but is not limited to RBC, WBC, epithelial cells, crystals, casts, and bacteria.

Urine safety analysis values will be evaluated by the Investigator for clinical relevance. Those deemed to be clinically significant will be reported as AEs.

5.15 Urine Drugs of Abuse and Nicotine Test

Urinalysis will be performed for drugs of abuse and cotinine using commercially available kits that provide an instant result, which will be documented in the source data.

Drugs of abuse screen will include (but is not limited to) amphetamines, barbiturates, cocaine, opiates, cannabinoids and benzodiazepines.

5.16 Pregnancy Test

Female participants are to have a urine pregnancy test at screening and at quarantine prior to inoculation (Day 0) and a serum pregnancy test on either day -2/-1. Participants will only be enrolled if the pregnancy tests are negative.

Note: Pregnancy test must be performed even if the participant is menstruating at the time of the study visit.

5.17 Blood Samples

A maximum volume of 550 mL of blood may be taken from each participant in any 8-week period (e.g. from screening through to the Day 28 follow up visit). If additional samples are required in excess of this amount, e.g., to monitor abnormalities, these will be collected at the discretion of the Investigator.

Blood will be taken for multiple purposes including:

- Eligibility, safety and pathogenicity assessments (haematology, biochemistry, and other eligibility safety monitoring tests) Safety blood samples and Appendix 2
- Immunology assays
- Exploratory research

Further instructions for the collection, handling, storage, shipment and analysis of samples are provided in the Analytical Plan.

5.17.1 Safety blood samples

Appendix 2 describes the safety blood tests that will be performed including, but not limited to, haematology, biochemistry and cardiac enzymes. Additional safety assessments (e.g. coagulation) will be conducted at the discretion of the PI/Investigator, as required

5.17.2 Immunology and sero-suitability to challenge virus

Participants must be sero-suitable to take part in the study, i.e. he/she must have no detectable pre-existing serum levels of antibodies specific to SARS-CoV-2.

Serum levels of pre-existing SAR-COV-2 specific antibodies to the Challenge Virus will be determined as described in the AP.

Additional exploratory immunological assays may be performed, as described in Exploratory Research.

5.18 Respiratory Samples

The following exploratory respiratory sampling procedures will be performed during the study:

- Mid turbinate FLOQ swab (or nasopharyngeal FLOQ swab)
- Mid-turbinate and/or nasopharyngeal swab for cells for RNA
- Throat FLOQ swab
- Saliva
- Nasosorption

Sample collection, handling, storage, shipment and analysis of samples from the respiratory tract will be performed in accordance with hVIVO SOPs, or as detailed in the Analytical Plan, as appropriate. These samples will be collected for:

- Viral loads
- Respiratory Pathogen Screen
- SARS-COv-2 confirmation of infection for intervention treatment
- SAR-COV-2 discharge test
- Exploratory purposes e.g. immunology

Tolerance of the procedure may be determined at the screening visit.

5.18.1 Viral load

Viral load will be determined by quantitative reverse transcription polymerase chain reaction (qRT-PCR) and a viral culture assay to investigate the following parameters:

- Infectivity status and rate
- viral dynamics (e.g. duration, peak, time to peak)

5.18.2 Respiratory pathogen screen

On entry to quarantine, a nasopharyngeal swab or nasal wash will be collected and tested to detect the presence of a set of respiratory pathogens, including Covid-19, that could potentially contraindicate a participant's participation in the study. The methodology to be used to conduct the respiratory virus screen will be documented in the Analytical Plan. Additional test may be conducted if the results from the first test were invalid to support study eligibility prior to virus inoculation, or if a community acquired infection is suspected during quarantine.

Any additional screening tests will be conducted at the discretion of the CI/PI.

5.18.3 SARS-CoV-2 confirmation of infection for intervention treatment

Infection will be determined by quantitative RT-PCR, of mid-turbinate and or throat swab samples taken 12 hourly. Intervention treatment can commence, if indicated and available after viral detection (≥LLOQ) in two consecutive upper respiratory tract samples by PCR.

5.18.4 SAR-CoV-2 confirmation of infection endpoint analysis

Infection will be determined by quantitative RT-PCR, quantitative viral culture and/or culture of mid-turbinate and throat swab samples taken 12 hourly. This is detailed in the objectives and endpoints.

5.18.5 SARS-CoV-2 discharge process

Subjects will remain in the quarantine unit for a minimum of 14 days after inoculation, until the following criteria are met:

1. 2 consecutive swabs with qPCR Ct >33.5 AND negative culture.
2. Lateral flow tests may be used in place of culture if culture is not available.
3. A qualitative PCR may be used in place of quantitative PCR, if the latter is not available.
   At the PI’s discretion if protracted quarantine is deemed to be causing harm to the participant’s mental or physical health and no viable virus is detected by culture or a lateral flow test is negative.

Discharge criteria for non-infected subjects

1. 2 consecutive swabs with undetectable virus by PCR only prior to discharge.

If, on the 14th day of residence post challenge the discharge criteria above are met then participants will be discharged from the confinement facility at the discretion of the PI.

Participants will be advised that they should stay in the quarantine unit they meet discharge criteria.

If a participant wishes to leave quarantine after virus inoculation but before discharge criteria are met, they will be counselled on risk of transmission, instructed on infection control measures and reminded to isolate within their own home. As part of the counselling, participants will be reminded that any household members will also need to self-isolate according to PHE and UK government guidelines as potential contacts of a COVID-19 case; that “rescue” or pre-emptive treatment is not possible in the community and therefore they will not receive it; and that they must remain in close contact with the study team to be monitored for any safety signals. The Health Protection Unit local to the participant’s home will be informed and any additional infection control measures recommended by them will be implemented.

In the event that a participant is suspected of becoming unwell or suffers a SAE and requires further treatment, the participant will be provided with immediate medical management followed by discussion with the PI/CI for possible referral for further assessment in accordance with “Criteria for Clinical Escalation of Participants” or elsewhere as appropriate (see Withdrawal criteria). NHS Hospitalisation of any subject, will lead to the immediate suspension of the trial. The DSMB will be convened to assess the clinical evidence in order to determine whether the study may proceed (see Data Safety Monitoring Board).

5.18.6 Post-discharge process

Self-isolation post-discharge

- If viable virus is no longer detectable by culture or lateral flow test following 10 days of quarantine after the first positive SARS-CoV-2 PCR test or first day of symptoms consistent with COVID-19 (whichever is later), discharged participants will be advised at the PI’s discretion that any additional self-isolation post-discharge will be in line with government guidelines.

Self-swabbing post discharge

- Weekly combined nose and throat swabs will be arranged for subjects who show persistent PCR detectable virus shedding. If swab results raise any concern about infectiousness, e.g. PCR value <33.5, then a lateral flow test can be performed. If the lateral flow test is negative isolation is not required.
- Additional swabs will not be required once a single swab has returned a not detected result.

Where low level virus can still be detected, participants will be reminded at each contact with the study team that they must follow government guidelines regarding infection control (including hand-washing and social distancing) and avoid contact with high-risk individuals.

5.19 Environmental Sampling

Exploratory environmental sampling may be conducted one day before (Day -1) and up to twice daily every day following intranasal inoculation of volunteers with SARS- CoV-2 (from Day 1).

Daily sampling will entail:

- Swabs collected from bedside furniture, door handles, computer keyboards, mobile phone and toilet floor in the volunteer quarters. Each day volunteer rooms will undergo a surface clean to avoid contamination from the previous day's virus deposition.

- Air sampling to detect airborne virus will be performed using a variety of devices. At certain time points volunteers may be asked to sing or speak loudly and collect samples before and after. Other than generating some noise, the samplers do not have any other impact on study participants.

Following collection, samples will be kept cold and returned to the laboratory for downstream processing. This may involve different viral assays such as:

- Quantitation of extracted viral RNA using two different RT-PCRs to E gene and polymerase gene
- Quantitation of infectious material by immunofluorescence: inoculation onto Vero cells, overnight incubation and staining of fixed cells with antibody to SARS CoV2 N protein. N positive cells are counted to give a read out of numbers of infectious foci in each sample.

Room temperature and humidity will be recorded. Baseline room ventilation rates (air changes per hour) will be measured.

5.20 Mask wearing sampling

As an additional exploratory assessment of virus in exhaled breath, volunteers will be asked to wear a single use face mask that has been fitted with a polyvinyl alcohol (PVA, Orbi-Tech, Leichlingen, Germany) sampling matrix insert, capable of capturing virus. They will do this on Study day -1 and then on a daily basis from Study Day 1 up to a maximum of 3 times a day for 60 - 90 minutes on each occasion. No hazards have been identified by the manufacturer of the PVA material and these face masks have been validated for the detection of M. tuberculosis in infected participants (Williams et al., 2020b). They have recently also been successfully demonstrated to detect SARS-CoV-2 in exhaled breath (Williams et al., 2020a). Up to five PVA strips will be harvested from exposed masks and analysed for virus and virus-related signals.

5.21 Exploratory research

The primary, secondary, and tertiary endpoints may be explored in relation to immunological levels at baseline and after SARS-CoV-2 challenge. Assays performed on blood and respiratory samples may include, but are not limited to:

- Humoral immunity / systems serology SARS-CoV-2 (for example: SARS-CoV-2 neutralizing titres, ELISAs to IgG, IgM, IgA, sIgA, ADCC)
- Proteomic levels and changes (for example, cytokine and chemokines)
- Cellular cell quantification and quality of immunity (for example T and B cell frequencies, phenotypes and functionality assays, ELISPOTs, ICS, cytokine/chemokine responses)
- Transcriptome levels and changes (for example, RNAseq, single cell RNAseq, microarray, PCR)
- Human genomics in relation to SARS-CoV-2 susceptibility, infection (e.g. HLA typing, SNPs, GWAS)
- Viral genomics in relation to SARS-CoV-2 population changes through infection
- Microbiome analysis in relation to viral infection, disease and susceptibility (e.g. PCR, NGS, 16s rRNA)

Results of these analyses may be reported separately to the final study report.

5.22 Optional Exploratory Non-Interventional Study of Wearable Device

Participants may be given the option to take part in a separate ethically-approved exploratory non-interventional trial that aims to collect physiological data via a wearable device. Participants will have the wearable device study explained to them and a choice to opt into the study, which will require them to consent to wear a wearable device (such as a wrist- or arm-band during the challenge trial, via an additional informed consent, which will be separately ethically-approved).

There will be no impact on participants eligibility and/or safety follow-up or treatment in the challenge trial. The wearable device will not replace any conventional safety monitoring. All subjects will be monitored for adverse events related to the wearable device from signing of the wearable device ICF to the end of data capture. An adverse event related to the wearable device may lead to discontinuation of the exploratory wearable study, without having an impact on the human challenge trial.

6. Recording of Adverse Events and Serious Adverse Events

The PI is responsible for ensuring that all AEs, SAEs and pregnancies are identified, evaluated, recorded and reported in a timely manner as per hVIVO's SOPs, and also for ensuring that the medical management (including follow up) of AEs, SAEs and, where appropriate, pregnancy symptoms/complications is provided by site staff.

The definitions of an AE or SAE, as well as the method of recording, evaluating, and assessing causality of AE and SAE and the procedures for completing and transmitting AE, SAE, and other reportable safety event reports can be found in Appendix 4.

6.1 Time Period and Frequency for Collecting AE and SAE Information

All AEs/SAEs will be collected from the signing of the ICF until the last Follow-up Visit at the time points specified in the SoA (Table 1).

Investigators are not obligated to actively seek AE or SAE after conclusion of the study participation. However, if the Investigator learns of any SAE, including a death, at any time after a participant has been discharged from the study, and he/she considers the event to be reasonably related to the study intervention or study participation, the Investigator must promptly notify the sponsor.

6.2 Method of Detecting AEs and SAEs

The method of recording, evaluating, and assessing causality of AE and SAE and the procedures for completing and transmitting SAE reports are provided in Appendix 4.

Care will be taken not to introduce bias when detecting AEs and/or SAEs. Open-ended and non-leading verbal questioning of the participant is the preferred method to inquire about AE occurrences.

6.2.1 Follow-up of AEs and SAEs

After the initial AE/SAE report, the Investigator is required to proactively follow each participant at subsequent visits/contacts. Additional Information/clarification may be required to ensure accurate completion of safety reports. The follow up reports should include a detailed Investigator's clinical assessment. All AEs/SAEs, will be followed until resolution, stabilisation, the event is otherwise explained, or the participant is lost to follow-up (as defined in Section 4). Further information on follow-up procedures is provided in Appendix 1.

6.2.2 Regulatory reporting requirements of SAEs

Prompt notification by the Investigator (PI) to the CI and Sponsor of a SAE is essential so that legal obligations and ethical responsibilities towards the safety of participants and the safety of a study intervention under clinical investigation are met.

SAEs will be documented and reported in accordance with hVIVO SOPs:

PI will send SAE/pregnancy forms to the delegated Pharmacovigilance (PV) provider within 24 hours of becoming aware of the event. PI will also inform the CI and Sponsor (RGIT.ctimp.team@imperial.ac.uk) at the same time.

The sponsor ensures compliance with country-specific regulatory requirements relating to safety reporting to the regulatory authority, Institutional Review Boards (IRB)/Independent Ethics Committees (IEC), and Investigators.

An Investigator who receives an investigator safety report describing a SAE or other specific safety information (e.g., summary or listing of SAEs) from the sponsor will review and will notify the IRB/IEC, if appropriate according to local requirements.

Further information on regulatory reporting requirements is provided in Appendix 1.

6.3 Reporting of events related to REGEN-COV

Any SAEs deemed to be related to REGEN-COV will be reported within 24 hours of investigator awareness following hVIVO SOP - P_SOP_Clin_0084.

Furthermore, we will report to Regeneron the following adverse events of special interest (AESI) related to REGEN-COV.:

- Grade ≥2 infusion-related reaction or Grade ≥2 hypersensitivity reaction. Where:

  i. Infusion-related reactions are defined as any relevant adverse events that occurs during the infusion or up to day 4.
  ii. Hypersensitivity reactions are defined as any relevant adverse event that occurs during the infusion or up to study day 29.

The only data that will be transferred to Regeneron will be restricted solely to safety information as outlined above.

6.3.1 Pregnancy

Details of all pregnancies in female participants and, if indicated, female partners of male participants will be collected from study specific informed consent and until the last study assessment as outlined in the SoA. If a pregnancy is reported, the Investigator should inform the sponsor within 24 hours of learning of the pregnancy and should follow the procedures outlined in Appendix 4..

Additionally, identification of pregnancy in a female participant within 6 months of receipt of REGEN-COV will be reported within 24 hours to Regeneron via a standardised reporting form. Optional consent for reporting of pregnancy in female partners of male participants within 6

months of receipt of REGEN-COV will also be sought. Outcomes for all participant pregnancies (and consented partner pregnancies) will be reported to Regeneron.

Abnormal pregnancy outcomes (e.g., spontaneous abortion, foetal death, stillbirth, congenital anomalies, ectopic pregnancy) are considered SAEs.

6.3.2 Disease-related events and/or disease related outcomes not qualifying as AEs or SAEs

Not applicable.

6.3.3 Treatment of overdose

For this study, any dose of any drug administered as part of the study greater than the dose prescribed by the protocol will be considered an overdose.

In the event of an overdose. the Investigator should:

1. Contact the Medical Monitor (Dr. Chris Chiu, Chief Investigator) immediately.
2. Closely monitor the participant for any AE/SAE and laboratory abnormalities associated with overdose and participants will be clinically followed up until the AE has resolved.
3. Document the quantity of the excess dose as well as the duration of the overdose in the CRF.

The Sponsor delegate (hVIVO) is responsible for notifying the REC of the potential serious breach within 7 days of becoming aware of it.

6.4 Safety Oversight Procedures

6.4.1 Procedures to be followed in the event of abnormal findings

Eligibility for enrolment in the trial in terms of laboratory findings will be assessed by clinically qualified staff. Abnormal clinical findings from medical history, examination or blood tests will be assessed as to their clinical significance throughout the trial. Laboratory AEs will be assessed using specific toxicity grading scales adapted from the FDA Toxicity Grading Scale for Healthy Adult and Adolescent Volunteers Enrolled in Preventive Vaccine Clinical Trials (see Appendix 3). If a test is deemed clinically significant, it may be repeated, to ensure it is not a single occurrence. If a test remains clinically significant, the volunteer will be informed and appropriate medical care arranged as appropriate and with the permission of the volunteer. Decisions to exclude the volunteer from enrolling in the trial or to withdraw a volunteer from the trial will be at the discretion of the Investigator.

6.4.2 Ongoing and interim safety reviews

The safety profile will be assessed on an on-going basis by the study Investigators. The CI/PI and relevant Investigators (as per the trial delegation log) will be reviewing safety issues and SAEs as they arise. The Chief Investigator will be informed of any safety concerns.

Safety reviews are planned as follows:

- After the first 3 volunteers at each dose level (Sentinels), available safety data will be reviewed by the PI, CI and relevant investigators, before inoculating further participants
- After each cohort has been challenged, a review will be performed by the chair of Data Safety Monitoring Board (DSMB) (or full DSMB at the discretion of the DSMB chair) before proceeding with challenge of further volunteers.

The DSMB will review safety data accumulated after each cohort and evaluate frequency of events, safety and infection rate / symptom data. The DSMB will make recommendations to the investigators concerning the conduct, continuation or modification of the study.

6.4.3 Safety holding rules

Safety holding rules have been developed considering the fact that this is the first study in which SARS-CoV-2 virus has been administered experimentally.

6.4.4 Group holding rules

Group holding rules below will apply:

- **Adverse events:**
  - If 2 or more individuals suffer the same Grade 3 adverse event persisting at Grade 3 for >72 hrs following virus challenge
- **Laboratory adverse event:**
  - If 2 or more individuals suffer the same Grade 3 laboratory adverse event persisting at Grade 3 for >72 hrs following virus challenge
- **A serious adverse event considered possibly, probably or definitely related to inoculation occurs**
- **Withdrawal from the study of 2 or more individuals due to subjectively intolerable symptoms considered possibly, probably or definitely related to SARS-CoV-2 inoculation**

If a holding rule is activated, then further virus inoculations will not occur until a safety review by the DSMB, study Sponsor, CI and PI has been conducted and it is deemed appropriate to restart challenges. Follow-up visits and procedures, including safety assessments for all study participants already challenged with the virus will continue. Intervention treatment will be given as planned unless the adverse events in question are deemed possibly, probably or definitely related to such treatment. The safety review will consider:

- The relationship of the AE or SAE to the virus inoculation, or other possible causes of the event.
- If appropriate, additional screening or laboratory testing for other volunteers to identify those who may develop similar symptoms and alterations to the current Participant Information Sheet (PIS)

- New, relevant safety information from ongoing research programs on COVID-19.

The ethics committee will also be notified if a holding rule is activated or released.

6.5 Data Safety Monitoring Board

The Data Safety Monitoring Board (DSMB) will be appointed for this study to periodically review and evaluate the accumulated study data for participant safety, study conduct, progress, and efficacy and make recommendations concerning the continuation, modification, or termination of the trial. Specifically, the DSMB will meet before study start and following the discharge of each challenged group.

The DSMB will operate in accordance with the study-specific charter, which will be established before recruitment starts.

The chair of the DSMB may be contacted for advice and independent review by the Investigator or Sponsor in the following situations:

- Following any SAE deemed to be possibly, probably or definitely related to a study intervention.
- Any other situation where the Investigator or trial Sponsor feels independent advice or review is important.

The DSMB will review SAEs deemed possibly, probably or definitely related to study interventions. The DSMB will be notified within 24 hours of the Investigators' being aware of their occurrence. The DSMB has the power to place the study on hold if deemed necessary following a study intervention-related SAE. Additionally, the DSMB will review safety data after each dosing cohort to assess the safety of progressing to the next cohort at the same dose, higher dose or lower dose. The DSMB is expected to meet approximately every 4 weeks during the period in which quarantines are taking place or as required.

6.6 Trial Steering Committee (also known as Medical Oversight Committee)

A Trial Steering Committee (TSC) will be convened to provide overall guidance for the project on behalf of the Project Sponsor (Imperial College London) and to ensure the project is conducted to the rigorous standards set out in the Department of Health's Research Governance Framework for Health and Social Care and the Guidelines for Good Clinical Practice. It should be noted that the day-to-day management of the trial is the responsibility of the Chief Investigator (Chris Chiu). The Chief Investigator in conjunction with the Project Delivery Group and the Sponsor's Project Manager is responsible for overseeing Trial management and progress. The TSC will comprise at least 3 independent members (including the Chair). The TSC will operate in accordance with the study-specific charter, which will be established before recruitment starts.

6.7 Human Challenge Steering Committee

Per the collaboration agreement signed by all collaborating partners a Human Challenge Steering Committee (HCSC) will be convened to provide overall guidance for the project on behalf of the Project Funder (UK Vaccines Taskforce, Department of Business, Energy & Industrial Strategy) and Partners to ensure the project is conducted in a timely and high quality manner.

The study will be terminated if this is recommended by the DSMB and/or TSC following any safety review. Additionally, the study will be terminated if data becomes available that raises concern about the safety of the study so that continuation would have posed significant new risks to the subjects. If the Investigators, DSMB, TSC or any member of the HCSC become aware of such data, they will call an extraordinary meeting to discuss the implications and assess the risk to study participants in light of the new data, after which the DSMB and TSC will provide a recommendation as to whether the study can continue.

6.8 Pharmacokinetics

Pharmacokinetic parameters are not evaluated in this study.

6.9 Pharmacodynamics

Pharmacodynamic parameters are not evaluated in this study.

7. Statistical Methods and Planned

This section describes the statistical analyses that will be performed in this study. Full details of the planned statistical analysis will be presented in the Statistical Analysis Plan (SAP). Any deviations from the SAP will be documented in the Clinical Study Report (CSR).

7.1 Study Analysis Sets

The following analysis sets are defined for this study:

- **Full Analysis Set (FAS)** is defined as all subjects that are inoculated with wild-type SARS-COV-2 challenge virus. The FAS will be considered as the primary analysis set for all primary, secondary and exploratory endpoints.
- **Safety Analysis Set** is defined as all subjects that are inoculated with wild-type SARS-COV-2 challenge virus. The Safety Analysis Set is identical to FAS, and will be used for all safety endpoints.
- **Per Protocol (PP) Analysis Set** is defined as all FAS subjects that are sero-suitable, who have no major protocol deviations and who complete the quarantine period up to the final day of quarantine (study Day 14). The PP Analysis Set will be considered as the secondary analysis set for pre-specified primary, secondary and exploratory endpoints.

Membership of subjects in each analysis set will be determined at a planned data review meeting (DRM), prior to any analysis and database lock.

7.2 Subgroup Analysis

A ‘Laboratory confirmed infected’ subgroup will be identified and will be presented for certain pre-specified analyses (as described in this protocol and/or documented in the SAP). The ‘subgroup is defined as those subjects that fulfil the following criteria:

- Two quantifiable (≥LLOQ) RT-PCR measurements from mid turbinate or throat samples, reported on 2 or more consecutive timepoints, starting from 24 hours post-inoculation and up to discharge from quarantine.

The subjects not part of the ‘Laboratory confirmed infected’ (i.e. those that are uninfected) will also be presented for certain pre-specified analyses.

Some pre-specified endpoints will additionally be explored in relation to subsets of symptoms from the symptom diary card.

Additional subgroup analysis may be performed, as detailed in the SAP (e.g., summarizing subjects that received a given dose level with a specific intervention treatment or not).

7.3 Sample Size

The primary objective of the study is to identify a safe and infectious dose of wild type SARS-CoV-2 in healthy volunteers, suitable for future intervention studies. No formal sample size calculation has been performed for this early stage dose finding study. However, a sample size of up to an expected 30 subjects for a dose level and treatment regimen (made up of 3 cohorts of up to an expected 10 subjects, including the dose expansion cohort), with a total of up to 90 subjects overall, is felt sufficient to meet the primary objective of escalating/expanding the dose in a safe manner whilst providing information on the attack rate.

The initial dose level administered will include a sentinel group of 3 subjects. As described in Section 4, if none of the 3 subjects in this initial dose level sentinel group become infected then the dose will be escalated, i.e. based on a target attack rate of 70%, the probability of seeing at least 1 subject in the 3 sentinel subjects being infected is 97.3%. Hence, if no subjects are infected then dose escalation will occur.

See below for further statistical rationale for the dose escalation/expansion scheme.

7.4 Cohort and Dose Escalation

An overview of the cohort and dose escalation scheme is presented in Figure 4. The following describes the statistical aspects to support the cohort and dose escalation scheme in relation to the first, second and third groups each of an expected 10 subjects (i.e. the first, second and third cohorts, respectively) at each dose level.

To support future studies, a target attack rate of 70% has been deemed desirable and should not be below 50%. A two-sided 95% confidence interval (CI) approach will be used for assessing the precision of the point estimate for the attack rate. This approach is consistent with previous human infection dose escalation studies[43].This approach is consistent with previous human infection dose escalation studies. Given that sample sizes are relatively small, an exact (Clopper-Pearson) confidence interval will be used. The assessment of the early (first and second) cohorts of the dose escalation scheme will be based primarily on safety together with clinically relevant fixed attack rate criteria. Subject numbers will then be expanded with a third cohort at the selected dose level and a 95% CI calculated to obtain a level of precision for the observed attack rate.

First Cohort:

For the first cohort, criteria for escalation, de-escalation and expansion, require no further statistical justification, i.e. dose expansion, escalation and de-escalation are based on a combination of safety and a clinically relevant fixed attack rate criterion.

Second Cohort:

For the second cohort, criteria for escalation, de-escalation and expansion are based on combination of safety and clinically relevant fixed attack rate criterion. A group size made up of subjects from the first and second cohorts will be used to show early indications of the attack rate. However, this is not based on statistical considerations.

Third Cohort (expansion group):

The dose expansion cohort should only take place when it is believed that an appropriate dose has been identified after the completion of the second cohort. Using "≥70% infected" as the criterion to expand to the third cohort, is a reasonable criterion to use based on targeting a 70% target attack rate, but also reducing the risk of seeing an attack rate of less than 50%, after an expected 30 subjects. This is similar to previous human infection dose escalation studies. If however an observed ≥70% attack rate after the first 2 cohorts at any dose level has not been achieved (even after reaching the highest dose) then expansion could be undertaken at the "most promising" dose (providing the attack rate after the second cohort is high enough. So, if a 70% attack rate is never reached after the first 2 cohorts at any dose level then the "≥70% infected" criterion becomes a "guide".

For the level of precision, once say 30 subjects are recruited, and an observed 21 out of 30 (70%) subjects are infected then this provides a 95% CI of (51%, 85%), and therefore this provides the necessary CI width to have the lower bound above the required 50%. In addition to this, by recruiting the third cohort (e.g. 30 subjects in total), this allows for inspection of any variation in the attack rate between the 3 cohorts at a particular dose level, and also increases the chance of potentially seeing any less frequent adverse events

7.5 Interim Statistical Analysis

No formal interim statistical analysis will be performed. However, two analyses are planned to be conducted. The primary analysis of study data will be conducted when all data through the Day 28 post-inoculation are available, monitored, and locked for all subjects. This will be followed by a final analysis which will include all study data through to the extended (Day 365) follow-up.

7.6 Statistical Analysis Plan

Data will be analysed and reported using SAS® version 9.4 or later.

No statistical comparison of dose groups is planned. Primary, secondary and exploratory endpoints will be analysed descriptively. Continuous variables will be summarised using number of observations, mean (and/or geometric mean, where applicable), standard deviation, standard error, median, lower quartile, upper quartile, minimum and maximum values. Categorical variables will be summarised using proportions (counts and percentages). A 95% confidence interval (CI) may be presented for certain pre-specified endpoints.

An SAP will be developed and approved prior to any lock of the study database. The SAP will give a more detailed description of the report presentations to be produced for the study, expanding on the protocol specified analysis. Any deviation(s) from the original statistical plan will be described and justified in an amendment to the protocol and/or SAP as appropriate and also referenced in the final clinical study report (CSR). The SAP will describe and account for the occurrence of and extent of missing data, and its possible impact on the study analysis. Any required sensitivity analyses will be specified in the SAP.

Further post-hoc evaluations of any exploratory endpoints may be conducted and reported separately.

7.6.1 Subject Accountability

The number of subjects receiving Challenge Virus, receiving at least one dose of intervention treatment withdrawing from the study (also split by reason for withdrawal), completing the study, and the numbers in each analysis set, will be summarised by dose group and across all subjects.

7.6.2 Protocol deviations

Subject data will be reviewed for major protocol deviations prior to database lock at a planned Data Review Meeting (DRM), and decisions will be documented within the meeting minutes. At this meeting, subjects will be reviewed for their inclusion/exclusion from the analysis sets. Protocol deviations will be listed.

7.6.3 Demographic and baseline characteristics

Descriptive statistics of demographics (age, sex, height, weight, BMI, and ethnicity) will be presented by dose group and across all subjects. Medical history information will be listed. Other baseline characteristics will be defined in the SAP.

7.7 Primary Endpoint Analysis

The primary objective is to identify a safe and infectious dose of wild type SARS-CoV-2 in healthy volunteers, suitable for future intervention studies.

The following primary endpoints will be analysed as follows:

- To evaluate the safety of wild type SARS-CoV-2 challenge in healthy subjects the following endpoints will be summarised by dose group, as well as the subgroups of infected and uninfected, and for all subjects:
    - Occurrence of unsolicited AEs within 30 days post-viral challenge (Day 0) up to Day 28 follow up.
    - Occurrence of SAEs related to the viral challenge from the viral challenge (Day 0) up to Day 28 follow up.

  Additional safety data presentations are also described in Section 5.
- To evaluate the infection rate, the laboratory confirmed infection rate will be summarised by dose group.

  The laboratory confirmed infection is defined as: Two quantifiable greater than lower limit of quantification (≥LLOQ) RT-PCR measurements from mid turbinate and/or, throat samples, reported on 2 or more consecutive timepoints, starting from 24 hours post-inoculation and up to discharge from quarantine.

In addition, the laboratory confirmed infection will be assessed using at least two detectable (≥LLOD) in place of quantifiable (≥LLOQ) RT-PCR measurements and will be summarised by dose group.

7.8 Secondary Endpoint Analysis

Secondary endpoints as described in Section 2 will be summarised by dose group as per Section 7, as appropriate. In addition, subgroup analyses may be performed as per Section 7. Further details will be described in the SAP.

7.9 Exploratory Endpoints

Exploratory endpoints as described in Section 2 may be summarised by dose group as per Section 7, as appropriate. In addition, subgroup analyses may be performed as per Section 7. Further details will be described in the SAP.

7.10 Safety Analysis

Safety data will be summarised descriptively.

All Adverse Events (AEs) will be coded using the most current version of the Medical Dictionary for Regulatory Activities (MedDRA). Virus Challenge emergent adverse events will be summarised by MedDRA system organ class (SOC), preferred term and dose group, for those subjects infected and uninfected and for all subjects.

An AE will be defined as Virus Challenge emergent if the onset date is on or after the date of inoculation. Any AE with an onset date earlier than the date of inoculation will be considered as a pre-Virus Challenge AE. If a subject experiences more than one AE with the same preferred term, that preferred term will be counted only once within summary presentations. It will be assigned the highest severity and the strongest relationship to Virus Challenge among those events for the summaries in which those characteristics are considered. Pre-Virus Challenge AEs will be identified in a listing.

Summary presentations will be performed for the number and percentage of subjects reporting Virus Challenge emergent: AEs, severity of AEs and AEs related to Virus Challenge. In addition, SAEs and AEs directly resulting in withdrawal from the study will be listed.

Other safety endpoints that will be presented by dose group include laboratory evaluations (biochemistry, haematology, coagulation, cardiac enzymes and urine analysis), vital signs assessments, 12-lead ECG, Forced Oscillation Technique (FOT) and spirometry. Additionally, physical examinations will be listed.

Concomitant medications will be coded using the most current version of the World Health Organization (WHO) Drug Dictionary. A medication will be assigned as being prior to Virus Challenge or concomitant with Virus Challenge, based on the start and stop dates of the medication and the date of inoculation. If the medication stop date is before the date of

inoculation, the medication will be assigned as being prior to Virus Challenge. In all other situations, the medication will be assigned as being concomitant with Virus Challenge. Concomitant medications will be separately summarised by WHO drug class, generic name and dose group. Prior medications will be identified in a listing. If a subject has separate periods of taking specific medications, then that medication is only counted once within the specific period of observation (i.e. prior or concomitant) where it is taken.

Further details of the safety analyses will be documented in the SAP.

8. Appendices: Supporting Documentation and Operational Considerations

Appendix 1: Regulatory, Ethical, and Study Oversight Considerations

8.1.1 Regulatory and ethical considerations

- This study will be conducted in accordance with the protocol and with the following:
  - Consensus ethical principles derived from international guidelines including the Declaration of Helsinki and Council for International Organizations of Medical Sciences (CIOMS) International Ethical Guidelines
  - Applicable ICH Good Clinical Practice (GCP) Guidelines
  - Applicable laws and regulations
- The protocol, protocol amendments, ICF and other relevant documents (e.g., advertisements) must be submitted to an IRB/IEC by the Chief Investigator and reviewed and approved by the IRB/IEC before the study is initiated.
- Any amendments to the protocol will require IRB/IEC approval before implementation of changes made to the study design, except for changes necessary to eliminate an immediate hazard to study participants. These will be reviewed by Sponsor prior to submission.
- The sponsor delegate, hVIVO, will be responsible for the following:

  - Notifying the IRB/IEC of SAEs or other significant safety findings as required by IRB/IEC procedures.
  - Providing written summaries of the status of the study to the IRB/IEC annually or more frequently in accordance with the requirements, policies, and procedures established by the IRB/IEC.
  - Provide Gilead and Regeneron with necessary information to facilitate safety reporting related to Remdesivir and REGEN-COV respectively
  - Providing oversight of the conduct of the study at the site and adherence to requirements of 21 CFR, ICH guidelines, the IRB/IEC, European regulation 536/2014 for clinical studies (if applicable), and all other applicable local regulations

8.1.2 Financial disclosure

Investigators and co-Investigators will provide the sponsor with sufficient, accurate financial information as requested to allow the sponsor to submit complete and accurate financial certification or disclosure statements to the appropriate regulatory authorities. Investigators are responsible for providing information on financial interests during the course of the study.

8.1.3 Funding

This study is funded by the UK Vaccines Taskforce and Department of Business, Energy and Industrial Strategy. They are acting as sole funders and the contract and agreement are in place. The investigators will not receive any additional payment above their normal salaries. Participants will be given a donation of up to £4565 to compensate for the time and inconvenience of taking part in the study (including at least 16-day quarantine). This was calculated using the NIHR formula and national living wage. It is similar to the market rate for in-patient human infection challenge studies. These expenses will be paid in line with standard hVIVO procedures. Where participants have an extended quarantine stay beyond Day 14 post-inoculation, the additional days will be reimbursed on a *pro rata* basis according to the number of days they additionally attended. Where participants have completed the confinement period but fail to attend further follow-up appointments, they will be reimbursed on a *pro rata* basis according to the number of visits they have attended. This will be detailed fully in the PIS.

8.1.4 Informed Consent Process

Before any study specific procedures are attempted, study physician will again discuss the study and study procedures with the potential participant and answer any questions. After that, participants will be asked to read, sign and date the full study ICF in the presence of the study doctor or nurse, who will also sign the consent form. A copy of the consent will be given to the participant and another copy will be filed in their medical notes.

The Investigator will obtain a signed screening ICF from each participant before any screening or study specific procedures are performed as described in section 2.

Potential participants will be sent a copy of the Screening ICF when their Screening Visit is arranged and at least one day prior to the visit, and will be encouraged to read it prior to their appointment. Upon arrival at the Screening Visit the Screening ICF is discussed, and they will be given the opportunity to ask any questions, and may take the information sheet away to consider their participation. Participants will be informed that a further discussion with the full study PIS/ICF will be scheduled at a later point (if necessary) and that they will be free to withdraw at any point before or after that.

All participants will be required to have a good understanding of English and the Investigator will be responsible for ensuring that the subject understands the information contained in the ICFs. Once they have confirmed that the subject has understood the study, including the benefits and risks of participation, the subject and the Investigator can sign and date the ICF.

The ICF must be signed and dated by the subject and countersigned by the Investigator (whoever conducted the consent discussion). A copy of the ICF will be given to the subject, and the original will be held in the full study TMF. The consent process will be documented in line with hVIVO SOPs, which require that documented and signed evidence must be available to confirm staff obtaining consent are trained and passed as competent, prior to independently obtaining consent. The Investigator will document the consent process in the subject notes and progress notes as applicable. This documentation will include details of capacity assessment, discussion with subject, and when/how consent has been obtained.

Participants will be assured that they can withdraw from the study at any time and for any reason without prejudice to their future medical care, and that they will be informed in a timely manner if new information becomes available that may affect their willingness to continue their participation in the study. This information will be included within the ICF.

The ICF will contain a separate section that addresses the use of samples for future research. The investigator or authorised designee will explain to each participant the objectives of the exploratory research. Participants will be told that they are free to refuse to participate and may withdraw their consent at any time and for any reason.

8.1.5 Confidentiality

The Chief Investigator will preserve the confidentiality of participants taking part in the study and is registered under the Data Protection Act.

8.1.6 Data protection

Participants will be assigned a unique 6-digit subject number by hVIVO Any participant records or datasets that are transferred to the sponsor will contain the identifier only; participant names or any information which would make the participant identifiable will not be transferred.

The participant must be informed that his/her personal study-related data will be used by the sponsor in accordance with local data protection law. The level of disclosure must also be explained to the participant who will be required to give consent for their data to be used as described in the informed consent.

The participant must be informed that his/her medical records may be examined by Clinical Quality Assurance auditors or other authorized personnel appointed by the sponsor or by appropriate IRB/IEC members.

8.1.7 Indemnity

Imperial College London holds negligent harm and non-negligent harm insurance policies which apply to this study. In addition, hVIVO and the Royal Free Hospital hold negligent harm insurance policies which apply to this study.

8.1.8 Sponsor

Imperial College London will act as the sponsor for this study. Delegated responsibilities will be assigned to hVIVO and the Royal Free Hospital taking part in this study.

8.1.9 Audits

The study may be subject to audit by Imperial College London under their remit as sponsor and other regulatory bodies to ensure adherence to GCP and the UK Policy Framework for Health and Social Care Research.

8.1.10 Sample and data storage and usage

Samples of tissue, cells and fluids will be stored according to Imperial College London and hVIVO SOPs as appropriate. Samples will be anonymised with participant identification numbers only and the anonymisation key kept in a separate locked location accessible only to the clinical study team. Samples may be used for further assays or in other ethically approved studies. Samples and data may be shared with UK and international collaborators in studies that have been approved by local ethics committees and subject to a valid Materials Transfer Agreement. Data and samples sent outside the UK will be pseudonymised with no patient identifiable data transferred. Data and all appropriate documentation will be stored for a minimum of 10 years after the completion of the study, including the follow-up period according to Imperial College London policy.

8.1.11 Dissemination of clinical study data

The key design elements of this Protocol will be posted on publicly accessible registers, such as ClinicalTrials.gov. Where required, protocol summaries will also be posted on national or regional clinical trial registers or databases (e.g., EudraCT database) in compliance with the applicable regulations.

It is the Sponsor's (or Sponsor delegate) responsibility to send the Clinical Trial Summary Report to the REC (if required) within 1 year of the end of the trial. In addition, the Sponsor or Sponsor delegate is responsible for entering appropriate data into the EudraCT results database within 1 year of the end of the trial.

The CI/PI/Investigator shall provide assurance to participants that their confidentiality will be maintained. hVIVO have a legal obligation to protect at all times the confidentiality of participant personal data from the point of capture, through processing, dissemination in line with consent from the participant and to its final disposition.

8.1.12 Data quality assurance

Participant data will be collected at site using paper source casebooks which will then be data entered into the electronic case report form (eCRF) database unless transmitted to the sponsor or designee electronically (e.g., laboratory data). The Investigator is responsible for verifying that data entries are accurate and correct by physically or electronically signing the CRF.

The Investigator must maintain accurate documentation (source data) that supports the information entered in the CRF.

The Investigator must permit study-related monitoring, audits, IRB/IEC review, and regulatory agency inspections and provide direct access to source data documents. Monitoring details describing strategy (e.g., risk-based initiatives in operations and quality such as Risk Management and Mitigation Strategies and Analytical Risk-Based Monitoring), methods, responsibilities and requirements, including handling of noncompliance issues and monitoring techniques (remote or on-site monitoring) are provided in the Monitoring Plan.

Study monitors will perform ongoing source data verification to confirm that data entered into the CRF by authorized site personnel are accurate, complete, and verifiable from source documents; that the safety and rights of participants are being protected; and that the study is being conducted in accordance with the currently approved protocol and any other study agreements, ICH GCP, and all applicable regulatory requirements.

Records and documents, including signed ICFs, pertaining to the conduct of this study must be retained by the Investigator during the retention period as agreed with the sponsor and as required by local regulations or institutional policies. No records may be destroyed during the retention period without the written approval of the Sponsor. No records may be transferred to another location or party without written notification to the Sponsor.

8.1.13 Source documents

Source documents provide evidence for the existence of the participant and substantiate the integrity of the data collected. Source documents are filed at the Investigator's site.

Data reported on the CRF or entered in the eCRF that are transcribed from source documents must be consistent with the source documents or the discrepancies must be explained. The Investigator may need to request previous medical records or transfer records, depending on the study. Also, current medical records must be available.

Definition of what constitutes source data can be found in the Source Data Agreement.

8.1.14 Study discontinuation

The Sponsor reserves the right to temporarily suspend or discontinue the study for any reason at any time. In addition, the study may be stopped at any time if, in the opinion of the CI, the safety data suggest that the medical safety of participants is being compromised.

If the study is suspended or terminated for safety reason(s), the Sponsor will promptly inform the PI, and will also inform the regulatory authorities of the suspension or termination of the study and the reason(s) for the action.

The CI is responsible for promptly informing the REC and providing the reason(s) for the suspension or termination of the study.

If the study is prematurely terminated, all study data must be returned to the Sponsor. In addition, the site must conduct final disposition of all unused intervention treatment in accordance with the Sponsor's procedures for the study.

Termination of the clinical trial may also be initiated by the REC.

8.1.15 Study management and governance

The study will be registered on https://clinicaltrials.gov/.

This study is part of the Human Challenge Programme overseen by the Vaccine Taskforce and funded by the Department of Business, Energy and Industrial Strategy (BEIS) of Her Majesty's Government. The programme is overseen by the Hu cman Challenge Steering Committee,

which reports into the Human Challenge Board. The Human Challenge board in turn reports to the Vaccine Taskforce Steering Group. overseen

The Human Challenge Steering Committee is the projects operational governing and advisory committee in relation to the scope, budget and timelines of the project. It has operational oversight, governance and decision-making rights of the study with respect to scope, timelines and budget. It has no authority with respect to decisions concerning medical or safety aspects of the subjects in the study.

The Medical Oversight Committee (also known as the TSC) is an independent committee with representatives appointed by the sponsor. The Medical Oversight Committee shall have authority with respect to decisions concerning the delivery of the protocol or any medical or clinical aspects of the project and/or any subjects of the study. The Medical Oversight Committee may provide information to the study Delivery team or the HCSC via their sponsor Chair.

The Study Delivery Team is responsible for the projects operational management and has day-to day responsibility for the management of the project but with no authority with respect to medical or safety aspects of the study. The study delivery team reports to the HCSC on performance against the service agreements and study milestones.

8.1.16 Publication policy

By signing the study protocol, the CI/PI agrees that the results of this study may be used for the purposes of national and international registration, publication, and information for medical and pharmaceutical professionals by the Sponsor and collaboration partners.

In order to allow the use of the information derived from this clinical study, the CI/PI understands that he has an obligation to provide complete test results and all data developed during this study to the Sponsor.

If the study is to be published, the Sponsor and collaboration partners may jointly prepare and co-author manuscript(s) that could result from the clinical trial. In the case the Sponsor acts as fully responsible for the publication, the Sponsor agrees to allow the partners time to review all manuscripts and abstracts prior to submission for publication. All proposed publications that discuss or disclose any part of the Study Data will be submitted to the Human Challenge Steering Committee (HCSC) and will be subject to the prior approval of the Human Challenge Steering Committee, or its successor The Sponsor also reserves the right to delete any confidential information from any proposed manuscripts prior to submission for publication. Confirmation of study specific arrangements can be found in the partnership collaboration agreement.

The expectation is that after analysis aggregated anonymised data from this study will be widely distributed in the medical and scientific community. Facilitated with presentations at local, national and international meetings, we hope to publish widely in the medical literature. In addition, we will work with public engagement and involvement groups and the media department at Imperial College and the collaboration partners to publicise research that is of public interest. No personal data from the participants will be published.

Appendix 2: Clinical Laboratory Tests

- The tests detailed in Table 12 will be performed The Doctor's Laboratory (TDL).
- Protocol-specific requirements for inclusion or exclusion of participants are detailed in Section 3 of the protocol.
- Additional tests may be performed at any time during the study as determined necessary by the Investigator or required by local regulations.
- Pregnancy Testing.

Table 12 Protocol-Required Safety Laboratory Assessments

Laboratory Assessments	Parameters
**Haematology**	Platelet Count.<br>White blood cell (WBC) count (absolute)<br>WBC differential:<br>Neutrophils<br>Lymphocyte<br>Monocytes<br>Eosinophils<br>Basophils<br>Red blood cell (RBC) count<br>Reticulocyte count (% and absolute)<br>Haemoglobin<br>Haematocrit<br>Mean corpuscular volume (MCV)<br>Mean corpuscular haemoglobin (MCH)<br>MCH concentration (MCHC).
**Coagulation**	Prothrombin Time (PT)<br>Activated Partial Thromboplastin Time (APTT)<br>D-dimer
**Biochemistry**	Sodium<br>Potassium<br>Glucose (random)<br>HbA1c (only required at screening)<br>Albumin<br>Chloride<br>Bicarbonate<br>Calcium<br>Uric acid<br>Total protein<br>Creatinine<br>eGFR<br>Total, direct, and indirect bilirubin

Laboratory Assessments	Parameters
	Inorganic phosphate<br>Blood urea nitrogen<br>C-reactive protein (CRP)<br>Gamma glutamyl transferase<br>Alkaline phosphatase (ALP)<br>Alanine transaminase (ALT)<br>Lactate dehydrogenase (LDH)<br>Aspartate transaminase (AST)<br>Urea.
**Thyroid function**	Thyroid Stimulating Hormone (TSH)<br>Thyroxine
**Cardiac enzymes**	Creatine Kinase (CK)<br>CK-MB<br>Troponin (T)<br>High sensitivity Troponin (T)
**Routine urinalysis**	Colour<br>Specific gravity<br>Appearance<br>pH<br>Presence of blood, glucose, leukocytes, ketones, nitrites, proteins, urobilinogen, bilirubin by dipstick<br>Microscopy, culture and sensitivity examination (If the dipstick yields abnormal results)
**Other screening/eligibility tests**	Follicle stimulating hormone (FSH)*<br>Total cholesterol (and full lipid profile at Investigator’s discretion)<br>Thyroid function test [thyroid stimulating hormone (TSH), free thyroxine (T4)]<br>Antibodies against HIV-1 and HIV-2<br>Hepatitis A immunoglobulin M (HepA)<br>Hepatitis B surface antigen (HBsAg)<br>Hepatitis C antibodies (HepC)

*Only for post-menopausal women

Investigators must document their review of each laboratory safety report.

Laboratory results that could unblind the study will not be reported to investigative sites or other blinded personnel until the study has been unblinded.

Appendix 3: Toxicity Grading Scale for Lab AEs

Toxicity Grading Scale for Lab AEs			Lab Range	Grade 1	Grade 2	Grade 3	Grade 4
**Sodium**	**Elevated**	**mmol/L**	145	146-147	148-149	150-155	>155
**Sodium**	**Low**	**mmol/L**	135	132-134	130-131	125-129	<125
**Potassium**	**Elevated**	**mmol/L**	5.1	5.2-5.3	5.4-5.5	5.6-6.5	>6.5
**Potassium**	**Low**	**mmol/L**	3.5	3.2-3.3	3.0-3.1	2.5-2.9	<2.5
**Urea**	**Elevated**	**mmol/L**	1.7-8.3	8.4-9.3	9.4-11.0	>11.0	Requires Dialysis
**Creatinine (Female)**	**Elevated**	**µmol/L**	49-92	101-138	139-276	>277	Requires Dialysis
**Creatinine (Male)**	**Elevated**	**µmol/L**	66-112	123-168	169-336	>336	Requires Dialysis
**Bilirubin**	**Normal LFTs**	**µmol/L**	0-20	22-30	31-40	41-60	>60
**Bilirubin**	**Abnormal LFTs**	**µmol/L**	0-20	22-25	26-30	31-35	>35
**ALP (Female)**	**Elevated**	**IU/L**	104	114-208	209-312	313-1040	>1040
**ALP (Male)**	**Elevated**	**IU/L**	129	142-258	259-387	388-1290	>1290
**ALT (Female)**	**Elevated**	**IU/L**	35	39-88	89-175	176-350	>350
**ALT (Male)**	**Elevated**	**IU/L**	50	55-125	126-250	251-500	>500
**AST (Female)**	**Elevated**	**IU/L**	31	34-78	79-155	156-310	>310
**AST (Male)**	**Elevated**	**IU/L**	37	41-93	94-185	186-370	>370
**Albumin**	**Low**	**g/L**	34-50	28-31	25-27	<25	-

			**Lab Range**	**Grade 1**	**Grade 2**	**Grade 3**	**Grade 4**
**Haemoglobin Absolute**	**Male**	**g/L**	130 – 170	115-125	100-114	85-99	<85
**Haemoglobin Absolute**	**Female**	**g/L**	115 – 155	105-113	90-104	80-89	<80
**Haemoglobin Decrease from Baseline**			n/a	0-15	16-20	21-50	>50
**White Blood Cells**	**Elevated**	**10^9/L**	3.0 – 10.0	11.5-15.0	15.01-20	20.01-25	>25
**White Blood Cells**	**Low**	**10^9/L**	3.0 – 10.0	2.0-3.5	1.5-1.99	1.0-1.49	<1.0
**Platelets**	**Low**	**10^9/L**	150 – 400	125-140	100-124	25-99	<25
**Neutrophils**	**Low**	**10^9/L**	2.0 – 7.5	1.5-1.99	1.0-1.49	0.5-0.99	<0.5
**Lymphocytes**	**Low**	**10^9/L**	1.2 – 3.65	0.75-0.99	0.3-0.74	0.25-0.29	<0.25
**Eosinophils**	**Elevated**	**10^9/L**	0.0 – 0.4	0.65-1.5	1.51-5.00	>5.00	Hyper-eosinophilia

Appendix 4: Adverse Events: Definitions and Procedures for Recording, Evaluating, Follow-up, and Reporting

8.4.1 Adverse event

AE Definition
An AE is any untoward medical occurrence in a volunteer, which may occur during or after administration of an intervention and does not necessarily have a causal relationship with the intervention. An AE can therefore be any unfavourable and unintended sign (including any clinically significant abnormal laboratory finding or change from baseline), symptom or disease temporally associated with the study intervention, whether or not considered related to the study intervention.

Events <u>Meeting</u> the AE Definition
• Exacerbation of a pre-existing illness.<br>• Increase in frequency or severity of a pre-existing eVISodic condition.<br>• A condition detected or diagnosed after inoculum administration even though it may have been present prior to the start of the study.<br>• A complication that occurs during a hospitalisation.
**Events <u>NOT</u> Meeting the AE Definition**
• Medical or surgical procedures (e.g., surgery, endoscopy, tooth extraction, transfusion); the condition that leads to the procedure is an AE.<br>• Pre-existing disease or conditions present or detected prior to start of Challenge Virus inoculation that does not worsen (including screening findings such as abnormal laboratory results).<br>• Hospitalisation for elective surgery, social and/or convenience admissions provided they are arranged before the start of Challenge Virus administration.<br>• Over-administration of either the challenge virus or concomitant medication without any signs or symptoms.<br>• An uncomplicated pregnancy or an induced elective abortion to terminate a pregnancy without medical reason.<br>• Typical/normal viral symptoms on symptom diary cards.

8.4.2 Serious adverse event

SAE Definition
An SAE is an AE that results in any of the following outcomes, whether or not considered

related to the study intervention.
**a. Results in death**
**b. Is life-threatening**<br>The term 'life-threatening' in the definition of 'serious' refers to an event in which the participant was at risk of death at the time of the event. It does not refer to an event, which hypothetically might have caused death, if it were more severe.
**c. Requires inpatient hospitalization or prolongation of existing hospitalisation**<br>• In general, hospitalisation signifies that the participant has been detained (usually involving at least an overnight stay) at the hospital or emergency ward for observation and/or treatment that would not have been appropriate in the physician's office or outpatient setting. Complications that occur during hospitalization are AEs. If a complication prolongs hospitalization or fulfils any other serious criteria, the event is serious. When in doubt as to whether "hospitalisation" occurred or was necessary, the AE should be considered serious.<br>• Hospitalisation for elective treatment of a pre-existing condition that did not worsen from baseline is not considered an AE.
**d. Results in persistent disability/incapacity**<br>• The term disability means a substantial disruption of a person's ability to conduct normal life functions.<br>• This definition is not intended to include experiences of relatively minor medical significance such as uncomplicated headache, nausea, vomiting, diarrhoea, influenza, and accidental trauma (e.g., sprained ankle) which may interfere with or prevent everyday life functions but do not constitute a substantial disruption.
**e. Is a congenital anomaly/birth defect**
**f. Is an important medical event:**<br>• Important medical events' - some medical events may jeopardise the participant or may require an intervention to prevent one of the above characteristics/consequences. Such events should also be considered as 'serious' in accordance with the above definition.<br>• Medical judgement should be exercised in deciding whether an adverse event/reaction is serious. Important adverse events/reactions that are not immediately life threatening or do not result in death or hospitalisation but may jeopardise the participant or may require intervention to prevent one of the other outcomes listed in the definition above occurring, should also be considered serious. Details of the SAE must be provided.

8.4.3 Potential adverse events related to SARS-CoV-2 infection

Following viral challenge, pyrexia (or febrile illness) is expected and presumed to represent virus infection consequent to viral challenge. Additionally, symptoms including headache, malaise, rhinorrhoea, nasal congestion, sneezing, sore throat, and cough may occur as features of upper respiratory tract symptoms. These would not be captured as an AE except those that meet the definitions below, or if they are deemed to be clinically significant (in the opinion of the Investigator) to be classed as an AE, or require intervention (e.g. medication except paracetamol) (see Withdrawal criteria). All unexpected (in the opinion of the Investigator) symptoms will be captured as AEs. Symptoms lasting longer than 12 weeks post-inoculation and consistent with "long COVID" (such as fatigue, neurological symptoms and anosmia, see Background "Potential long-term complications of COVID-19 and "long COVID") will be classed as AEs.

Criteria for classing expected symptoms and signs as AEs are:

- Temperature Grade 2 or higher for >48 hours
- Headache Grade 3 or higher
- Cough Grade 3 or higher

Any clinical events set out in Withdrawal criteria that require a participant to be withdrawn from the study or pause in the study are defined as SAEs.

8.4.4 Recording, assessment and follow-up of AE and/or SAE

8.4.4.1 AE and SAE recording

All AEs and SAEs will be collected from the time of written informed consent until study completion/final study contact or until the resolution of the AE. AEs will be fully recorded in the source documents as they are reported, whether spontaneously volunteered by a subject or in response to questioning about wellbeing at each face to face study visit and during telephone calls. Enquiries about AEs should cover the period between the previous and current visit.

The following are examples of open ended, non-leading questions that may be used to obtain this information:

- How are you feeling?
- Have you had any medical problems since your last visit/assessment?
- Have you taken any new medicines, other than those given to you in this study, since your last visit/assessment?

Following the reporting of AEs and concomitant medication, the Investigator should assess the subject's eligibility to continue in the study.

The PI will record all relevant information regarding an AE/SAE in the source documents and evaluate AEs using the following guidelines:

- Description of events (if the event consists of a cluster of signs and symptoms, a diagnosis should be recorded)
- Seriousness
- Severity (or grade)
- Onset date and time
- Frequency
- Date and time of resolution (or ‘continuing’ if unresolved)
- Action taken
- Concomitant medication
- Clinical outcome
- Relationship or causality (Intervention treatment/ Challenge Virus/ study procedures/ concomitant medication/other).

Any clinically significant abnormal laboratory result, vital sign or other measure will be followed until it returns to normal or baseline values, stabilises, or is judged by the Investigator to be no longer clinically significant.

If an AE is not resolved at the end of the study, the AE should be followed until it has resolved or (in the case of pregnancy) the pregnancy has been terminated (including spontaneous abortion), resulted in a birth, or a decision has been made by the Sponsor that no further follow-up is required.

Even if the AE or SAE is assessed by the PI as not reasonably attributable to the challenge virus, its occurrence must be fully documented in the source notes.

8.4.4.2 Assessment

<u>Description</u>

If the event consists of a cluster of signs and symptoms, a diagnosis should be recorded (e.g. gastroenteritis) rather than each sign and symptom.

<u>Onset and end</u>

The dates and times of the onset and end of the event should be recorded.

**Assessment**
**Challenge Virus Symptoms**<br>The Investigator will assess, and review Challenge Virus related symptoms recorded in participants’ hVIVO Symptom Diary Cards. Symptoms greater than Grade 0 will be expected and presumed to represent virus infection consequent to Viral Challenge, and will not be additionally captured as AEs unless they meet the definition of an AE, and are deemed to be

clinically significant (in the opinion of the Investigator) to be classed as AEs.<br><br>Following Viral Challenge all unexpected (in the opinion of the Investigator) symptoms post inoculation will be captured as AEs, along with all other occurrences that meet the criteria for an AE.
**Physical Examination**<br><br>Any clinically significant change in complete physical examination findings during the study will be documented as an AE.
**Direct Physical Examination**<br><br>Following Viral Challenge, upper and lower respiratory symptoms (nasal discharge, otitis, pharyngitis, sinus tenderness, new wheezes, rales and rhonchi) will be expected and presumed to represent virus infection consequent to Viral Challenge, and will not be additionally captured as AEs unless they meet the definition of an AE, and are deemed to be clinically significant (in the opinion of the Investigator) to be classed as AEs.
**Vital Signs**<br><br>Deterioration in a vital sign (compared to baseline) should only be reported as an AE if the deterioration fulfils the criteria for an AE. If deterioration in a vital sign is associated with clinical signs and symptoms, the sign or symptom will be reported as an AE and the associated vital sign will be considered as additional information.
**Temperature**<br><br>Following Viral Challenge, pyrexia will be expected and presumed to represent virus infection consequent to Viral Challenge, and will not be additionally captured as an AE unless it meets the definition of an AE, and is deemed to be clinically significant (in the opinion of the Investigator) to be classed as an AE.<br><br>Following Viral Challenge all unexpected (in the opinion of the Investigator) pyrexia post inoculation will be captured as an AE, along with all other occurrences that meet the criteria for an AE.
**Spirometry**<br><br>A 15% drop in a spirometry value (compared to baseline, and confirmed by a repeat on the same day) may be judged a Grade 1 (mild) AE. However, due to variability in participants' ability to perform these tests with adequate technique, the Investigator and/or SME will use his/her clinical judgement to assess whether abnormal spirometry readings are consistent with a true drop and whether an AE should be raised. The PI/Investigator will use his/her clinical judgement to assign severity grades above Grade 1, based on evaluation of clinical signs and symptoms. If a spirometry reading on repeat assessment has returned to normal an AE will not be raised.
**Laboratory Values**

Deterioration in a laboratory value (compared to baseline) should only be reported as an AE if the deterioration fulfils the criteria for an AE. If deterioration in a laboratory result is associated with clinical signs and symptoms, the sign or symptom will be reported as an AE and the associated laboratory result will be considered as additional information.

The Investigator and/or SME will judge whether abnormal laboratory values are clinically significant or not clinically significant, and record this in the source document. This entry should be signed and dated by the relevant Investigator. Laboratory abnormalities detected at screening will be considered as part of the medical history and will not be reported as AEs

Challenge Virus associated laboratory abnormalities (e.g.: elevated ALT, AST or GGT; decreased neutrophils) may be recorded as AEs (at the discretion of the Investigator).

8.4.4.3 Assessment of intensity

The term 'severe' is often used to describe the intensity (severity) of a specific event. This is not the same as 'serious' which is based on participant/event outcome or action criteria.

The PI will use the grading scale for AEs as a reference when collecting, reporting and clarifying database queries of AEs and SAEs.

The severity of an AE that does not appear in the **grading scale for AEs** should be determined according to the definitions in Table 13.

Table 13 Classification of Adverse Event Severity

Grade	Definition
Grade 0	**Absent**
Grade 1	**Mild** level of discomfort, and does not interfere with regular activities
Grade 2	**Moderate** level of discomfort that intermittently interferes with regular activities
Grade 3	**Severe:** Significant level of discomfort and prevents regular activities
Grade 4	**Potentially life threatening**

An event is defined as 'serious' when it meets at least 1 of the predefined outcomes as described in the definition of an SAE, NOT when it is rated as severe. It is important to distinguish between serious and severe AEs. An AE of severe intensity needs not necessarily be considered serious. For example, a migraine headache that incapacitates a participant for many hours may be severe, whereas a stroke that results in a limited degree of disability may be considered mild but should be reported as a SAE.

Table 14 Severity grading criteria for physical observations

Vital Signs	Grade 1 (mild)	Grade 2 (moderate)	Grade 3 (severe)	Grade 4 Potentially Life threatening
**Fever (tympanic)**	**37.9°C - 38.4°C**	**38.5°C – 38.9°C**	**39.0°C - 40°C**	> 40°C
**Tachycardia (bpm)***	**101 – 115**	**116 – 130**	**>130**	A&E visit or hospitalisation for arrhythmia
**Bradycardia (bpm)****	**45-49**	**40-44**	**<40**	A&E visit or hospitalisation for arrhythmia
**Systolic hypertension (mmHg)**	**141 – 150**	**151 – 155**	**≥155**	A&E visit or hospitalization for malignant hypertension
**Diastolic hypertension (mmHg)**	**91 – 95**	**96 – 100**	**>100**	A&E visit or hospitalization for malignant hypertension
**Systolic hypotension (mmHg)*****	**85 – 89**	**80 – 84**	**<80**	A&E visit or hospitalization for hypotensive shock
**Respiratory Rate – breaths per minute**	**17 – 20**	**21-25**	**>25**	Intubation

*Taken after 5 minutes at rest **When resting heart rate is between 50 – 100 beats per minute. Use clinical judgement when characterising bradycardia among some healthy subject populations, for example, conditioned athletes. ***Only if symptomatic (e.g. dizzy/ light-headed)

8.4.4.4 Frequency

The frequency of the AE should be categorised as one of the following:

- Single
- Intermittent
- Continuous

8.4.4.5 Assessment of causality

For every AE, an assessment of the relationship of the event to the administration of the challenge virus will be undertaken by the PI. An interpretation of the causal relationship of the challenge virus to the AE in question will be made, based on the type of event; the relationship of the event to the time of challenge virus administration; and the known biology of the challenge virus (Table 9). Alternative causes of the AE, such as the natural history of pre-existing medical conditions, concomitant therapy, other risk factors and the temporal relationship of the event to challenge virus will be considered and investigated. Causality assessment will take place during planned safety reviews, interim analyses (e.g. if a holding or stopping rule is activated) and at the final safety analysis, except for SAEs, which should be assigned by the reporting investigator immediately.

- The Investigator is obligated to assess the relationship between challenge virus and each occurrence of each AE/SAE.
- A “reasonable possibility” of a relationship conveys that there are facts, evidence, and/or arguments to suggest a causal relationship, rather than a relationship cannot be ruled out.
- The Investigator will use clinical judgment to determine the relationship.
- Alternative causes, such as underlying disease(s), concomitant therapy, and other risk factors, as well as the temporal relationship of the event to study intervention administration will be considered and investigated.
- For each AE/SAE, the Investigator **must** document in the medical notes that he/she has reviewed the AE/SAE and has provided an assessment of causality.
- There may be situations in which an SAE has occurred, and the Investigator has minimal information to include in the initial report to PV lead provider. However, it is very important that the investigator always make an assessment of causality for every event before the initial transmission of the SAE data.
- The Investigator may change his/her opinion of causality in light of follow-up information and send a SAE follow-up report with the updated causality assessment.
- The causality assessment is one of the criteria used when determining regulatory reporting requirements – AE/SAE related to the challenge virus will be reported to the REC.
- The relationship of an AE to the challenge virus will be categorised as shown in

Table 15 Classification of Adverse Event Relationship

Classification	Definition
Not related	The AE is related to an aetiology other than the challenge virus (the alternative aetiology must be documented in the participant's medical record).
Unlikely to be related	The AE is unlikely to be related to the challenge virus and likely to be related to factors other than challenge virus
Possibly related	There is an association between the AE and the administration of the challenge virus, and there is a plausible mechanism for the AE to be related to the challenge virus, but there may also be alternative aetiology, such as characteristics of the participant's clinical status or underlying disease.
Probably related	A reasonable temporal sequence of the AE and the challenge virus administration exists and, based upon the known pharmacological action of the drug, known or previously reported adverse reactions to the drug or class of drugs, or judgment based on the Investigator's clinical experience, the association of the AE with the challenge virus seems likely.
Definitely related	A definite causal relationship exists between the AE and the administration of the challenge virus, and other conditions do not appear to explain the AE.

Unless an AE is 'definitely related' to the challenge virus, a causal relationship to one of the following should be considered, and full details provided on the AE reporting form as appropriate.

- Study procedures
- Concomitant medication
- Other

8.4.4.6 Action taken

The Investigator should ensure that adequate medical care is provided to participants for any AEs, including clinically significant laboratory values related to the study intervention. In addition, the Investigator will describe whether any treatment was given for the AE.

The Investigator will classify the action taken with regard to the AE. The action taken should be classified according to the following categories and full details provided as appropriate:

- None

- Non-drug therapy given
- Concomitant medication taken
- Study intervention dose not changed
- Study intervention dose adjusted
- Study intervention administration temporarily interrupted
- Study intervention administration permanently discontinued
- N/A Study intervention not administered
- Participant withdrawn
- Participant hospitalised
- Other

8.4.4.7 Outcome

An AE should be followed until the Investigator has determined and recorded the outcome or an alternative explanation. The outcome should be classified according to the categories shown in Table 16.

Table 16 Classification of Adverse Event Outcome

Classification	Definition
Resolved	Resolution of the AE with **no** residual signs or symptoms
Resolved with sequelae	Resolution of the AE with residual signs or symptoms
Ongoing	Either incomplete improvement or no improvement of the AE, such that it remains on-going
Fatal	Outcome of the AE was death. 'Fatal' should be used when death was at least possibly related to the AE.
Unknown (e.g. Lost to follow-up)	Outcome of the AE is not known (e.g. the participant is lost to follow-up).

8.4.4.8 Follow up

All AEs and SAEs must be followed-up by the Investigator, or where appropriate, be referred to the participant's GP or other healthcare professional for follow-up until they are:

- Resolved (return to normal or baseline values), or
- Stabilised, or
- Judged by the PI/Investigator to be no longer clinically significant, or
- An alternative explanation has been provided.

Additional measurements and/or evaluations may be necessary to investigate the nature and/or causality of an AE or SAE. This may include additional laboratory tests, diagnostic procedures, or consultation with other healthcare professionals. If the participant dies, any post-mortem findings (including histopathology) will be provided to the Sponsor if possible.

8.4.5 Reporting of SAEs

Prompt notification and assessment of SAEs to ethics committee(s) where necessary for this study will be the responsibility of the sponsor delegate (hVIVO). Contact details are detailed in Table 17

Annual safety/progress reports and final Study report will be generated and submitted to relevant ethics committee(s). This will be the responsibility of hVIVO as described in Section 6.

Notification should be made:

- In a detailed written SAE form report within 24 hours of the Investigator becoming aware of the event.

All reports should be directed to the pharmacovigilance mailbox. The Investigator at the site is responsible for ensuring that a member of the Sponsor study team is made aware of any SAE reports that have been transmitted.

Table 17 Contact Details for Reporting SAEs

[redacted]

In addition, any AE resulting in permanent study discontinuation for a participant, even if not serious and regardless of expectedness or causality, must be reported by telephone, email or fax to the CI and Sponsor within 7 calendar days of the PI or any other site personnel's knowledge of the event.

The SAE form, AE record and relevant concomitant medication record should be faxed/emailed to the Sponsor within 24 hours of the Investigator or any site personnel's knowledge of a SAE. An updated SAE report form should be forwarded to the Sponsor within 24 hours of receipt of the new/updated information as relevant.

Information relating to the participant's subsequent medical progress must be submitted to the Sponsor as available, until the SAE has subsided or, in the case of permanent impairment, until it stabilises and the overall clinical outcome has been ascertained.

The Investigator will also provide additional information, including a copy of the following documents (where applicable):

- Copies of test results, as available
- Hospital discharge summary (as soon as it is available to the PI)
- Autopsy report (as soon as it is available to the PI).

The Investigator must report SAEs to the relevant REC in accordance with applicable regulatory requirements and within the relevant timelines.

The REC will be sent annual safety updates in order to facilitate their continuing review of the study.

8.4.6 Adverse reactions to non-IMPS

Any AEs and SAEs which are related to/caused by a concomitant medication or Challenge agent, should not be classed as ARs, SARs, or SUSARs (ARs, SARs, SUSARs relate only to IMP by definition). However, an SAE caused by a non-IMP would need to be reported to the REC for the appropriate action to be taken.

8.4.7 Post-study AEs and SAEs

All SAEs that occur during the study must be reported by the Investigator to the SME as soon as possible, in accordance with hVIVO's SOPs, and at the latest within 24 hours of becoming aware of the event.

8.4.8 Pregnancy

If a female participant or partner of a male participant becomes pregnant during [specify time period], this must be reported by the Investigator to the medical monitor and Study Monitor by telephone as soon as possible, in accordance with hVIVO's SOPs, and at the latest within 24 hours of becoming aware of the event.

Following the telephone notification, the Investigator must fully and accurately complete the appropriate pregnancy reporting form, which must be e-mailed to the pharmacovigilance department and the Study Monitor at the latest within 24 hours of becoming aware of the pregnancy.

Participants will be advised to contact their GP or a specialist, as appropriate.

Provided that the appropriate consent is in place, information related to the pregnancy will be collected as per hVIVO's SOPs and the Sponsor's requirements. The completed reporting form(s) will be sent to the Sponsor for review and assessment, and subsequent reporting as required.

- A complete evaluation will be documented in the source data to permit transfer to the clinical database.

- hVIVO will maintain contact with the participant for a protracted period of time, but certainly until after the birth, in order to assess for outcomes that may be reportable as related AEs, and for reporting to the Sponsor as appropriate.
- hVIVO in consultation with the participant will keep the participant's GP informed.
- All cases of foetal drug exposure via the parent as a study participant will be reported to the Sponsor and the REC.

Appendix 5: Normal Ranges

Vital Signs

Vital sign Parameters	Lower limit	Higher limit	Units
Tympanic temperature (above 37.8 classed as pyrexia)	35.5	37.8	°C
Oxygen saturation	Normal is ≥ 95		%
Respiratory rate	10	17	breaths per minute
Heart rate	50	100	beats per minute
Systolic BP	90	140	mmHg
Diastolic BP	50	90	mmHg

ECG

ECG Parameters	Lower limit	Higher limit	Units
**HR**	**50**	**100**	**bpm**
**QRS**	**60**	**120**	**ms**
**PR interval**	**120**	**220**	**ms**
**QT**	**320**	**450**	**ms**
QTc	Normal for females is < 470		ms
	Normal for males is < 450		
QTcF	320	450	ms
QTcB	320	450	ms

Spirometry

Spirometry parameters	Lower limit	Higher limit	Units
FEV1	Normal if ≥ 80% of the predicted value		litres
FEV1/FVC	Normal if ≥70% (≥ 0.7) of the base value		litres

Appendix 6: Abbreviations

ADA	Anti-Remdesivir Antibody
ADCC	Antibody-Dependent Cellular Cytotoxicity
AE	Adverse Event
ALP	Alkaline Phosphatase
ALRI	Acute Lower Respiratory Infection
ALT	Alanine Aminotransferase
AP	Analytical Plan
APTT	Activated Partial Thromboplastin Time
AR	Adverse Reaction
AST	Aspartate Aminotransferase
AST	Aspartate Transaminase
AUC	Area Under the Curve
BP	Blood Pressure
BD	Twice Daily
BMI	Body Mass Index
cGMP	Current Good Manufacturing Practices
CK	Creatine Kinase
CHIM	Controlled human infection model
CI	Chief Investigator
CIOMS	Council for International Organizations of Medical Sciences
CMI	Cell Mediated Immunity
COPD	Chronic Obstructive Pulmonary Disease
CRF	Case Report Form
CRP	C-reactive Protein
CTL	Cytotoxic T cell
COVID-19	Coronavirus Disease 19
CYP450	Cytochrome 450
DBP	Diastolic blood pressure
DNA	Deoxyribonucleic acid
DMID	Division of Microbiology and Infectious Disease
DRM	Data review meeting
DSMB	Data Safety Monitoring Board
ECG	Electrocardiogram
ELISA	Enzyme-linked Immunosorbent Assay
ERS	European Respiratory Society
FAS	Full Analysis Set
FEV	Forced Expiratory Volume
FEV1	Forced Expiratory Volume in One Second
FOT	Forced oscillation technique
FSH	Follicle Stimulating Hormone

FVC	Forced Vital Capacity
GAD	Generalised Anxiety Disorder
GCP	Good Clinical Practice
GDPR	General Data Protection Regulation
GMP	Good Manufacturing Practice
GP	General Practitioner
HAV	Hepatitis A
HbA1c	Haemoglobin A1c
HBV	Hepatitis B
HCV	Hepatitis C
HIV	Human Immunodeficiency Virus
HVC	Human Viral Challenge
IB	Investigator Brochure
ICF	Inform Consent Form
ICH	International Council for Harmonisation
IEC	Independent Ethics Committees
IgA	Immunoglobulin A
IgG	Immunoglobulin G
IgM	Immunoglobulin M
IM	Intramuscular
IMP	Investigational Medicinal Product
IRB	Institutional Review Boards
IUD	Intrauterine Device
IV	Intravenous
LRT	Lower Respiratory Tract
MCH	Mean Corpuscular Haemoglobin
MCHC	Mean Corpuscular Haemoglobin Concentration
MCID	Minimal Clinically Important Difference
MCV	Mean Corpuscular Volume
MedDRA	Medical Dictionary for Regulatory Activities
MHRA	Medicines and Healthcare products Regulatory Agency
MOI	Monoamine Oxidase Inhibitors
NIMP	Non-Investigational Medicinal Product
NPS	Nasopharyngeal Swab
PBMC	Peripheral Blood Mononuclear Cell
PCA	Principal Component Analysis
PCR	Polymerase Chain Reaction
PEF	Peak Expiratory Flow
PFM	Peak flow meter
PFU	Plaque Forming Unit
PHQ	Patient Health Questionnaire
PI	Principal Investigator*

PIS	Participant Information Sheet
PK	Pharmacokinetic
PP	Per Protocol
PT	Prothrombin Time
QDS	Four Times Daily
qRT-PCR	Quantitative Reverse Transcriptase-Polymerase Chain Reaction
RBC	Red Blood Cell
REC	Research Ethics Committee
RNA	Ribonucleic acid
RSI	Reference Safety Information
RSV	Respiratory Syncytial Virus
SAR-COV-2	Severe Acute Respiratory Syndrome Coronavirus 2
SAE	Serious Adverse Event
SAP	Statistical Analysis Plan
SARS-CoV-2	Severe Acute Respiratory Syndrome Coronavirus 2
SBP	Systolic blood pressure
SoA	Schedule of Activities
SOC	System Organ Class
SOP	Standard Operating Procedure
SpO2	Peripheral arterial oxygen saturation
SUSAR	Suspected Unexpected Adverse Reaction
T	Troponin
TDS	Three Times Daily
TMF	Trial Master File
TSH	Thyroid Stimulating Hormone
TSS	Total Symptoms Score
UK	United Kingdom
UPSIT	University of Pennsylvania smell identification test
URT	Upper Respiratory Tract
WBC	White Blood Cell
WHO	Word Health Organisation
β-HCG	β-human chorionic gonadotrophin

* Delegation Log will list all investigators / study physicians delegated by PI to perform study activities.

9. References

Bagga, B., Woods, C.W., Veldman, T.H., Gilbert, A., Mann, A., Balaratnam, G., Lambkin-Williams, R., Oxford, J.S., McClain, M.T., Wilkinson, T., *et al.* (2013). Comparing influenza and RSV viral and disease dynamics in experimentally infected adults predicts clinical effectiveness of RSV antivirals. Antiviral therapy *18*, 785-791.

Beigel, J.H., Tomashek, K.M., Dodd, L.E., Mehta, A.K., Zingman, B.S., Kalil, A.C., Hohmann, E., Chu, H.Y., Luetkemeyer, A., Kline, S., *et al.* (2020). Remdesivir for the Treatment of Covid-19 - Final Report. The New England journal of medicine, NEJMoa2007764.

Berlin, D.A., Gulick, R.M., and Martinez, F.J. (2020). Severe Covid-19. The New England journal of medicine.

Berry, D., Berry, S., Hale, P., Isakov, L., Lo, A., Siah, K.W., and Wong, C.H. (2020). A Cost/Benefit Analysis of Clinical Trial Designs for COVID-19 Vaccine Candidates. medRxiv, 2020.2009.2015.20195495.

Bost, P., Giladi, A., Liu, Y., Bendjelal, Y., Xu, G., David, E., Blecher-Gonen, R., Cohen, M., Medaglia, C., Li, H., *et al.* (2020). Host-Viral Infection Maps Reveal Signatures of Severe COVID-19 Patients. Cell *181*, 1475-1488.e1412.

Brouwer, P.J.M., Caniels, T.G., van der Straten, K., Snitselaar, J.L., Aldon, Y., Bangaru, S., Torres, J.L., Okba, N.M.A., Claireaux, M., Kerster, G., *et al.* (2020). Potent neutralizing antibodies from COVID-19 patients define multiple targets of vulnerability. Science *369*, 643-650.

Buckland, M.S., Galloway, J.B., Fhogartaigh, C.N., Meredith, L., Provine, N.M., Bloor, S., Ogbe, A., Zelek, W.M., Smielewska, A., Yakovleva, A., *et al.* (2020). Treatment of COVID-19 with remdesivir in the absence of humoral immunity: a case report. Nat Commun *11*, 6385.

de Graaf, H., Ibrahim, M., Hill, A.R., Gbesemete, D., Vaughan, A.T., Gorringe, A., Preston, A., Buisman, A.M., Faust, S.N., Kester, K.E., *et al.* (2020). Controlled Human Infection With Bordetella pertussis Induces Asymptomatic, Immunizing Colonization. Clinical infectious diseases : an official publication of the Infectious Diseases Society of America *71*, 403-411.

DeVincenzo, J.P., McClure, M.W., Symons, J.A., Fathi, H., Westland, C., Chanda, S., Lambkin-Williams, R., Smith, P., Zhang, Q., Beigelman, L., *et al.* (2015). Activity of Oral ALS-008176 in a Respiratory Syncytial Virus Challenge Study. New England Journal of Medicine *373*, 2048-2058.

DeVincenzo, J.P., Whitley, R.J., Mackman, R.L., Scaglioni-Weinlich, C., Harrison, L., Farrell, E., McBride, S., Lambkin-Williams, R., Jordan, R., Xin, Y., *et al.* (2014). Oral GS-5806 activity in a respiratory syncytial virus challenge study. The New England journal of medicine *371*, 711-722.

DeVincenzo, J.P., Wilkinson, T., Vaishnaw, A., Cehelsky, J., Meyers, R., Nochur, S., Harrison, L., Meeking, P., Mann, A., Moane, E., *et al.* (2010). Viral load drives disease in humans experimentally infected with respiratory syncytial virus. American journal of respiratory and critical care medicine *182*, 1305-1314.

Docherty, A.B., Harrison, E.M., Green, C.A., Hardwick, H.E., Pius, R., Norman, L., Holden, K.A., Read, J.M., Dondelinger, F., Carson, G., *et al.* (2020). Features of 20 133 UK patients in hospital with covid-19 using the ISARIC WHO Clinical Characterisation Protocol: prospective observational cohort study. BMJ *369*, m1985.

Eyal, N., Lipsitch, M., and Smith, P.G. (2020). Human Challenge Studies to Accelerate Coronavirus Vaccine Licensure. The Journal of infectious diseases *221*, 1752-1756.

Gbesemete, D., Barker, M., Lawrence, W.T., Watson, D., de Graaf, H., and Read, R.C. (2020). Exploring the acceptability of controlled human infection with SARSCoV2—a public consultation. BMC medicine *18*, 209.

Goldman, J.D., Lye, D.C.B., Hui, D.S., Marks, K.M., Bruno, R., Montejano, R., Spinner, C.D., Galli, M., Ahn, M.Y., Nahass, R.G.*, et al.* (2020). Remdesivir for 5 or 10 Days in Patients with Severe Covid-19. The New England journal of medicine.

Grifoni, A., Weiskopf, D., Ramirez, S.I., Mateus, J., Dan, J.M., Moderbacher, C.R., Rawlings, S.A., Sutherland, A., Premkumar, L., Jadi, R.S.*, et al.* (2020). Targets of T Cell Responses to SARS-CoV-2 Coronavirus in Humans with COVID-19 Disease and Unexposed Individuals. Cell *181*, 1489-1501.e1415.

Guvenel, A., Jozwik, A., Ascough, S., Ung, S.K., Paterson, S., Kalyan, M., Gardener, Z., Bergstrom, E., Kar, S., Habibi, M.S.*, et al.* (2020). Epitope-specific airway-resident CD4+ T cell dynamics during experimental human RSV infection. The Journal of clinical investigation *130*, 523-538.

Habibi, M.S., Thwaites, R.S., Chang, M., Jozwik, A., Paras, A., Kirsebom, F., Varese, A., Owen, A., Cuthbertson, L., James, P.*, et al.* (2020). Neutrophilic inflammation in the respiratory mucosa predisposes to RSV infection. Science *370*.

Harrison, E.M.e.a. (2020). Ethnicity and Outcomes from COVID-19: The ISARIC CCP-UK Prospective Observational Cohort Study of Hospitalised Patients. The Lancet.

Hayden, F.G., Treanor, J.J., Fritz, R.S., Lobo, M., Betts, R.F., Miller, M., Kinnersley, N., Mills, R.G., Ward, P., and Straus, S.E. (1999). Use of the Oral Neuraminidase Inhibitor Oseltamivir in Experimental Human InfluenzaRandomized Controlled Trials for Prevention and Treatment. JAMA *282*, 1240-1246.

He, X., Lau, E.H.Y., Wu, P., Deng, X., Wang, J., Hao, X., Lau, Y.C., Wong, J.Y., Guan, Y., Tan, X.*, et al.* (2020). Temporal dynamics in viral shedding and transmissibility of COVID-19. Nature medicine *26*, 672-675.

Helleberg, M., Niemann, C.U., Moestrup, K.S., Kirk, O., Lebech, A.-M., Lane, C., and Lundgren, J. (2020). Persistent COVID-19 in an Immunocompromised Patient Temporarily Responsive to Two Courses of Remdesivir Therapy. The Journal of infectious diseases *222*, 1103-1107.

Horby, P., Lim, W.S., Emberson, J.R., Mafham, M., Bell, J.L., Linsell, L., Staplin, N., Brightling, C., Ustianowski, A., Elmahi, E.*, et al.* (2020). Dexamethasone in Hospitalized Patients with Covid-19 - Preliminary Report. The New England journal of medicine.

Humeniuk, R., Mathias, A., Cao, H., Osinusi, A., Shen, G., Chng, E., Ling, J., Vu, A., and German, P. (2020). Safety, Tolerability, and Pharmacokinetics of Remdesivir, An Antiviral for Treatment of COVID-19, in Healthy Subjects. Clin Transl Sci *13*, 896-906.

Iwasaki, A., and Yang, Y. (2020). The potential danger of suboptimal antibody responses in COVID-19. Nature reviews. Immunology *20*, 339-341.

Jiang, L., Tang, K., Levin, M., Irfan, O., Morris, S.K., Wilson, K., Klein, J.D., and Bhutta, Z.A. (2020). COVID-19 and multisystem inflammatory syndrome in children and adolescents. The Lancet. Infectious diseases *20*, e276-e288.

Jones, S., Evans, K., McElwaine-Johnn, H., Sharpe, M., Oxford, J., Lambkin-Williams, R., Mant, T., Nolan, A., Zambon, M., Ellis, J.*, et al.* (2009). DNA vaccination protects against an influenza challenge in a double-blind randomised placebo-controlled phase 1b clinical trial. Vaccine *27*, 2506-2512.

Jozwik, A., Habibi, M.S., Paras, A., Zhu, J., Guvenel, A., Dhariwal, J., Almond, M., Wong, E.H., Sykes, A., Maybeno, M.*, et al.* (2015). RSV-specific airway resident memory CD8+ T cells and differential disease severity after experimental human infection. Nat Commun *6*, 10224.

Kuiper, V.P., Rosendaal, F.R., Kamerling, I.M.C., Visser, L.G., and Roestenberg, M. (2020). Assessment of risks associated with SARS-CoV-2 experimental human infection studies. Clinical Infectious Diseases.

Lam, M.H., Wing, Y.K., Yu, M.W., Leung, C.M., Ma, R.C., Kong, A.P., So, W.Y., Fong, S.Y., and Lam, S.P. (2009). Mental morbidities and chronic fatigue in severe acute respiratory syndrome survivors: long-term follow-up. Archives of internal medicine *169*, 2142-2147.

Lambkin-Williams, R., Gelder, C., Broughton, R., Mallett, C.P., Gilbert, A.S., Mann, A., He, D., Oxford, J.S., and Burt, D. (2016). An Intranasal Proteosome-Adjuvanted Trivalent Influenza Vaccine Is Safe, Immunogenic & Efficacious in the Human Viral Influenza Challenge Model. Serum IgG & Mucosal IgA Are Important Correlates of Protection against Illness Associated with Infection. PloS one *11*, e0163089.

Long, Q.-X., Tang, X.-J., Shi, Q.-L., Li, Q., Deng, H.-J., Yuan, J., Hu, J.-L., Xu, W., Zhang, Y., Lv, F.-J.*, et al.* (2020). Clinical and immunological assessment of asymptomatic SARS-CoV-2 infections. Nature medicine *26*, 1200-1204.

Maxwell (2020). Living with Covid19.

McClain, M.T., Henao, R., Williams, J., Nicholson, B., Veldman, T., Hudson, L., Tsalik, E.L., Lambkin-Williams, R., Gilbert, A., Mann, A.*, et al.* (2016). Differential evolution of peripheral cytokine levels in symptomatic and asymptomatic responses to experimental influenza virus challenge. Clinical and experimental immunology *183*, 441-451.

Miller, M.R., Hankinson, J., Brusasco, V., Burgos, F., Casaburi, R., Coates, A., Crapo, R., Enright, P., van der Grinten, C.P., Gustafsson, P.*, et al.* (2005). Standardisation of spirometry. The European respiratory journal : official journal of the European Society for Clinical Respiratory Physiology *26*, 319-338.

Nabavi, N. (2020). Long covid: How to define it and how to manage it. BMJ *370*, m3489.

Ngai, J.C., Ko, F.W., Ng, S.S., To, K.W., Tong, M., and Hui, D.S. (2010). The long-term impact of severe acute respiratory syndrome on pulmonary function, exercise capacity and health status. Respirology (Carlton, Vic.) *15*, 543-550.

Nguyen-Van-Tam, J.S., Killingley, B., Enstone, J., Hewitt, M., Pantelic, J., Grantham, M.L., Bueno de Mesquita, P.J., Lambkin-Williams, R., Gilbert, A., Mann, A.*, et al.* (2020). Minimal transmission in an influenza A (H3N2) human challenge-transmission model within a controlled exposure environment. PLoS pathogens *16*, e1008704.

Pan, H., Peto, R., Karim, Q.A., Alejandria, M., Henao-Restrepo, A.M., García, C.H., Kieny, M.-P., Malekzadeh, R., Murthy, S., Preziosi, M.-P.*, et al.* (2020). Repurposed antiviral drugs for COVID-19 – interim WHO SOLIDARITY trial results. medRxiv, 2020.2010.2015.20209817.

Plotkin, S.A., and Caplan, A. (2020). Extraordinary diseases require extraordinary solutions. Vaccine *38*, 3987-3988.

Polack, F.P., Thomas, S.J., Kitchin, N., Absalon, J., Gurtman, A., Lockhart, S., Perez, J.L., Pérez Marc, G., Moreira, E.D., Zerbini, C.*, et al.* (2020). Safety and Efficacy of the BNT162b2 mRNA Covid-19 Vaccine. New England Journal of Medicine.

Quanjer, P.H., Weiner, D.J., Pretto, J.J., Brazzale, D.J., and Boros, P.W. (2014). Measurement of FEF25-75% and FEF75% does not contribute to clinical decision making. The European respiratory journal : official journal of the European Society for Clinical Respiratory Physiology *43*, 1051-1058.

Roberts, A., Lamirande, E.W., Vogel, L., Jackson, J.P., Paddock, C.D., Guarner, J., Zaki, S.R., Sheahan, T., Baric, R., and Subbarao, K. (2008). Animal models and vaccines for SARS-CoV infection. Virus Res *133*, 20-32.

Rogers, T.F., Zhao, F., Huang, D., Beutler, N., Burns, A., He, W.-t., Limbo, O., Smith, C., Song, G., Woehl, J.*, et al.* (2020). Isolation of potent SARS-CoV-2 neutralizing antibodies and protection from disease in a small animal model. Science *369*, 956-963.

Sakurai, A., Sasaki, T., Kato, S., Hayashi, M., Tsuzuki, S.-I., Ishihara, T., Iwata, M., Morise, Z., and Doi, Y. (2020). Natural History of Asymptomatic SARS-CoV-2 Infection. The New England journal of medicine *383*, 885-886.

Salje, H., Tran Kiem, C., Lefrancq, N., Courtejoie, N., Bosetti, P., Paireau, J., Andronico, A., Hozé, N., Richet, J., Dubost, C.L., *et al.* (2020). Estimating the burden of SARS-CoV-2 in France. Science *369*, 208-211.

Shrestha, D.B., Budhathoki, P., Syed, N.-i.-H., Rawal, E., Raut, S., and Khadka, S. (2020). Remdesivir: A potential game-changer or just a myth? A systematic review and meta-analysis. Life Sciences, 118663.

Spiegelhalter, D. (2020). Use of “normal” risk to improve understanding of dangers of covid-19. BMJ *370*, m3259.

Stanojevic, S., Wade, A., Stocks, J., Hankinson, J., Coates, A.L., Pan, H., Rosenthal, M., Corey, M., Lebecque, P., and Cole, T.J. (2008). Reference ranges for spirometry across all ages: a new approach. American journal of respiratory and critical care medicine *177*, 253-260.

Sudre, C.H., Murray, B., Varsavsky, T., Graham, M.S., Penfold, R.S., Bowyer, R.C., Pujol, J.C., Klaser, K., Antonelli, M., Canas, L.S., *et al.* (2020). Attributes and predictors of Long-COVID: analysis of COVID cases and their symptoms collected by the Covid Symptoms Study App. medRxiv, 2020.2010.2019.20214494.

Tenforde, M.W. (2020). Symptom Duration and Risk Factors for Delayed Return to Usual Health Among Outpatients with COVID-19 in a Multistate Health Care Systems Network — United States, March–June 2020. MMWR Morb. Mortal. Wkly. Rep *69*.

Tillett, R.L., Sevinsky, J.R., Hartley, P.D., Kerwin, H., Crawford, N., Gorzalski, A., Laverdure, C., Verma, S.C., Rossetto, C.C., Jackson, D., *et al.* (2020). Genomic evidence for reinfection with SARS-CoV-2: a case study. The Lancet Infectious Diseases.

van Doremalen, N., Lambe, T., Spencer, A., Belij-Rammerstorfer, S., Purushotham, J.N., Port, J.R., Avanzato, V.A., Bushmaker, T., Flaxman, A., Ulaszewska, M., *et al.* (2020). ChAdOx1 nCoV-19 vaccine prevents SARS-CoV-2 pneumonia in rhesus macaques. Nature *586*, 578-582.

van Kampen, J.J.A., van de Vijver, D.A.M.C., Fraaij, P.L.A., Haagmans, B.L., Lamers, M.M., Okba, N., van den Akker, J.P.C., Endeman, H., Gommers, D.A.M.P.J., Cornelissen, J.J., *et al.* (2020). Shedding of infectious virus in hospitalized patients with coronavirus disease-2019 (COVID-19): duration and key determinants. medRxiv, 2020.2006.2008.20125310.

Wang, Y., Zhang, D., Du, G., Du, R., Zhao, J., Jin, Y., Fu, S., Gao, L., Cheng, Z., Lu, Q., *et al.* (2020). Remdesivir in adults with severe COVID-19: a randomised, double-blind, placebo-controlled, multicentre trial. Lancet *395*, 1569-1578.

Watson, J.M., Francis, J.N., Mesens, S., Faiman, G.A., Makin, J., Patriarca, P., Treanor, J.J., Georges, B., and Bunce, C.J. (2015). Characterisation of a wild-type influenza (A/H1N1) virus strain as an experimental challenge agent in humans. Virology journal *12*, 13-13.

Whittaker, E., Bamford, A., Kenny, J., Kaforou, M., Jones, C.E., Shah, P., Ramnarayan, P., Fraisse, A., Miller, O., Davies, P., *et al.* (2020). Clinical Characteristics of 58 Children With a Pediatric Inflammatory Multisystem Syndrome Temporally Associated With SARS-CoV-2. Jama *324*, 259-269.

WHO_Solidarity_Trial_Consortium (2020). Repurposed Antiviral Drugs for Covid-19 — Interim WHO Solidarity Trial Results (Dec). New England Journal of Medicine.

Wilkinson, T.M., Li, C.K., Chui, C.S., Huang, A.K., Perkins, M., Liebner, J.C., Lambkin-Williams, R., Gilbert, A., Oxford, J., Nicholas, B., *et al.* (2012). Preexisting influenza-specific CD4+ T cells correlate with disease protection against influenza challenge in humans. Nature medicine *18*, 274-280.

Williams, C., Pan, D., Decker, J., Wisniewska, A., Sze, S., Fletcher, E., Haigh, R., Abdulwhhab, M., Bird, P., Holmes, C., *et al.* (2020a). COVID-19: Exhaled virus detected by Face Mask Sampling provides new links to disease severity and potential infectivity. medRxiv, 2020.2008.2018.20176693.

Williams, C.M., Abdulwhhab, M., Birring, S.S., De Kock, E., Garton, N.J., Townsend, E., Pareek, M., Al-Taie, A., Pan, J., Ganatra, R., *et al.* (2020b). Exhaled Mycobacterium tuberculosis output and detection of subclinical disease by face-mask sampling: prospective observational studies. The Lancet. Infectious diseases *20*, 607-617.

Williamson, B.N., Feldmann, F., Schwarz, B., Meade-White, K., Porter, D.P., Schulz, J., Doremalen, N.v., Leighton, I., Yinda, C.K., Pérez-Pérez, L., *et al.* (2020). Clinical benefit of remdesivir in rhesus macaques infected with SARS-CoV-2. bioRxiv, 2020.2004.2015.043166.

Wölfel, R., Corman, V.M., Guggemos, W., Seilmaier, M., Zange, S., Müller, M.A., Niemeyer, D., Jones, T.C., Vollmar, P., Rothe, C., *et al.* (2020). Virological assessment of hospitalized patients with COVID-2019. Nature *581*, 465-469.
