# Supplementary material for: Changes in memory and cognition during the SARS-CoV-2 human challenge study
Source: eClinicalMedicine. 2024 Sep 21;76:102842. doi: 10.1016/j.eclinm.2024.102842 (PMC11447363; doi:10.1016/j.eclinm.2024.102842)
Supplement: Statistical analysis plan [file mmc2.docx]

# Statistical Analysis Plan

# Analysis Tools

All analyses were conducted using MatLab Version R2022b.

# Analysis Overview

1. Cleaning the data
2. Generating the analysis variables
3. Main Outcome analysis
4. Sensitivity analyses
5. Exploratory analyses

# 1 - Cleaning the data

## 1.1 - Cognitive Task Data

### 1.1.1 - Defining Non-compliant / Missing data

A number of different methods were used to automatically flag data that might be exhibiting non-compliant behaviour using the raw response outputs generated by the tasks.

Firstly, trial by trial reaction times were screened; if greater than 40% of the trials were responded to with a reaction time below a certain threshold then the task instance was flagged for non-compliance. This is to detect volunteers that are simply clicking through the response elements without engaging with the trial. This threshold was set manually per task by looking at the distribution of reaction times in the normative data collected elsewhere on these tasks. They were set at: Motor Control - 225ms, Object Memory (Immediate Recall) - 500ms, Simple Reaction Time - 100ms, Choice Reaction Time - 100ms, 2D Manipulations - 700ms, Four Towers - 1000ms, Spatial Span - 400ms, Target Detection - 440ms, Tower of London - 1000ms, Verbal Analogies - 500ms and Object Memory (Delayed Recall) - 500ms.

Secondly, repetitive response patterns were detected; if a volunteer responded repeatedly to the same response element (or area of the screen if there were no defined response elements) greater than 80% of the trials then that task instance was flagged as non-compliant.

There was also a small number of missing data points through either participant error (possible because the participants were performing the tasks unsupervised in their quarantine rooms) or through researcher error (possible if the battery of tasks was initialised incorrectly on the study devices.

### 1.1.2 - Interpolating Non-compliant / Missing Data

Once missing and non-compliant data had been identified, interpolation of these data points was performed. This was done in multiple ways:

#### 1.1.2.1 - Composite scores

1. During calculation of the bcGCCS (and other composite scores), the mean of multiple tasks was taken after normalisation and baseline correction. At this point, if there were under half of the task data missing for a given volunteer and timepoint then the composite score was calculated using only the available data.
2. If there were fewer than half of the tasks present for a given volunteer on a given timepoint then the composite score was calculated using linear interpolation of the neighbouring composite score timepoints in the timeline. The function used was the matlab function interp1 with the linear and extrapolation option applied.

#### 1.1.2.2 - Non-composite task scores

1. Individual task scores when they were not included in a composite score were interpolated using linear interpolation with the matlab function interp1 with the linear and extrapolation option applied.

#### 1.1.2.3 - Plasma markers of brain injury

1. Missing brain injury marker data was interpolated using class interpolation, where missing values were replaced by the mean of the group within the same time point.

# 2 - Generating Analysis Variables

## 2.1 - Baseline Corrected Global Composite Cognitive Score (bcGCCS)

In order to calculate the baseline corrected global cognitive composite score (bcGCCS) across the 11 cognitive task primary outcome measures a number of steps were taken. The task scores were standardised using the following procedure:

1. Standardise the individual task scores.
   1. Start with a matrix of values [volunteer x task x timepoint].
   2. Take the mean across volunteers for the baseline timepoints in the matrix from 1a (resulting in a matrix of values [task x baseline timepoint]).
   3. Take the standard deviation across volunteers for the baseline timepoints in the matrix from 1a (resulting in a matrix of values [task x baseline timepoint]).
   4. Take the mean across baseline days for the values calculated in 1b (resulting in a vector of values, 1 for each task).
   5. Take the mean across baseline days for the standard deviations calculated in 1c (resulting in a vector of values, 1 for each task).
   6. Subtract the task values calculated in 1d from the corresponding task values for every volunteer and time point in 1a (resulting in a matrix of baseline corrected values [volunteer x task x timepoint]).
   7. Take the matrix from step 1f and divide through every volunteer and timepoint value by the corresponding task value calculated in step 1e (resulting in a matrix of values [volunteer x task x timepoint] standardised by the baseline mean and standard deviation for each task).
2. Calculate the composite scores
   1. Take the mean across task for the matrix calculated in step 1g (resulting in a matrix of composite task scores [volunteer x timepoint]).
3. Standardise the composite scores
   1. Take the mean across volunteers for the baseline days of the matrix calculated in step 2a (resulting in a vector of mean values 1 for each baseline day).
   2. Take the standard deviation across volunteers for the baseline days of the matrix calculated in step 2a (resulting in a vector of standard deviation values 1 for each baseline day).
   3. Take the mean across baseline days for the values calculated in step 3a (resulting in a single mean of means of the baseline value).
   4. Take the mean across baseline days for the values calculated in step 3b (resulting in a single mean of standard deviations of the baseline value).
   5. Subtract the value calculated in step 3c from every value in the matrix calculated in step 2a (resulting in a mean corrected matrix [volunteer x timepoint]).
   6. Divide every value calculated in step 3e by the value calculated in step 3d (resulting in a standardised matrix of composite scores [volunteer x timepoint]).
4. Baseline correct the standardised composite score
   1. Take the mean across volunteers of the baseline timepoints for the matrix calculated in step 3f (resulting in a mean value for each baseline timepoint).
   2. Take the mean across values calculated in step 4a (resulting in a mean of means of the baseline timepoints).
   3. Subtract the value from step 4b from every value in the matrix from step 3f (resulting in a baseline corrected standardised composite score [volunteer x timepoint]).
5. Summarise across quarantine phase
   1. Take the matrix from step 4c and mean across the timepoints included in the quarantine (resulting in a single value for each volunteer).
   2. Concatenate the values calculated in step 5a with the followup timepoints from step 4c (resulting in a [volunteer x timepoint] matrix where the first column is the mean of the quarantine timepoints and the subsequent columns are each individual follow up timepoint).

## 2.2 - Individual task scores

Secondary to the main composite, the individual task scores were prepared for analysis in the following steps:

1. Baseline correct the standardised scores.
   1. Starting with the matrix from step 1g in section 2.1.
   2. Take the mean of the baseline timepoints across volunteers (resulting in a matrix of mean values for the baseline timepoints [task x timepoint]).
   3. Take the mean of the values across timepoint from the matrix from step 1b (resulting in a single mean value for each task).
   4. Subtract the values from step 1c from their corresponding task values in the matrix from step 1a for each volunteer and timepoint (resulting in a matrix of baseline corrected standardised scores [volunteer x task x timepoint]).
2. Summarise across quarantine phase
   1. Take the matrix from step 1d and mean across the timepoints included in the quarantine (resulting in a value for each task and volunteer).
   2. Concatenate the values calculated in step 2a with the followup timepoints from step 1d (resulting in a [volunteer x task x timepoint] matrix where the first timepoint is the mean of quarantine timepoints and the other timepoints are the follow up timepoints).

## 2.3 - Learning and Non-learning Composite scores

The first step to creating the learning and non-learning composite scores was to determine which tasks exhibited effects of learning and which did not. This was done by performing a repeated measures ANOVA on the data for the quarantine timepoints for the uninfected volunteers for each task individually using the matlab function fitrm. If a given task had a significant effect of time over the quarantine timepoint it was deemed to have a significant effect of learning. And the task were split into two groups based on this criteria. The Composites were then calculated in the same way as was described in section 2.1.

## 2.4 - Viral Load Data

The information on viral titre was collected via Quantitative polymerase chain reaction (qPCR) from both throat and nasal swab sites twice a day. In order to get the measure of viral load used in the analysis, the following steps were taken:

1. Summarise across swab site and time of day
   1. Start with a matrix of viral titre values [volunteer x timepoint x swab site x time of day].
   2. Take the mean of the matrix from step 1a across swab site and time of day (resulting in a matrix of mean viral titre values [volunteer x timepoint]).
2. Calculate the Area Under the Curve or “Total Viral Load”
   1. For each timepoint in the matrix from 1b take the sum of all previous timepoints up to and including the current timepoint (resulting in a matrix of areas under the curve for past viral titre up to each timepoint [volunteer x timepoint]).
3. Summarise across the quarantine phase
   1. Take the values from the final timepoint of quarantine in the matrix from step 2a (resulting in a vector of values that represent the area under the curve for the viral titre in the quarantine phase).
   2. Concatenate the values from step 3a with the follow up timepoints from the matrix from step 2a (resulting in a [volunteer x timepoint] matrix where the first column is the area under the curve over the quarantine timepoints and the subsequent columns are the area under the curve up to that timepoint).

## 2.5 - Temperature

Volunteers’ temperature was collected up to 4 times per day. These raw data were prepared for analysis in the following way.

1. Summarise across the time of day.
   1. Start with a matrix of viral titre values [volunteer x timepoint x time of day].
   2. Take the max of the matrix from step 1a across time of day (resulting in a matrix of maximum temperature values per timepoint [volunteer x timepoint]).
2. Baseline correct
   1. Take the mean across timepoint for the two baseline days from the matrix resulting from step 1b (resulting in a vector of baseline temperature values one for each volunteer).
   2. Subtract the baseline temperature values from step 2a from each subsequent timepoint temperature value (resulting in a baseline corrected set of temperature values [volunteer x timepoint]).
3. Extract peak change values
   1. Take the matrix from step 2b and take the maximum value from baseline up until a given timepoint for each timepoint within each volunteer (resulting in a matrix of peak change values up to each timepoint [volunteer x timepoint]).
4. Summarise the quarantine phase
   1. Take the values from the final timepoint of quarantine in the matrix from step 3a (resulting in a vector of values that represent the maximum temperature change over the quarantine phase).
   2. Concatenate the values from step 4a with the follow up timepoints from the matrix from step 3a (resulting in a [volunteer x timepoint] matrix where the first column is the maximum temperature change over the quarantine timepoints and the subsequent columns are the maximum temperature change up to that timepoint).

## 2.6 - Symptom load

The participants were given daily subjective symptom rating questionnaires where they were asked to rate the severity of their symptoms on a score of 0 to 3 where 0 was no symptoms and 3 was severe symptoms. The raw data were processed in the following way:

1. Determine which days each participant experienced symptoms.
   1. Start with a matrix of raw values [volunteer x symptom type x timepoint].
   2. Sum the values from the matrix in step 1a across symptom type (resulting in a matrix of total symptom scores for each volunteer and timepoint [volunteer x timepoint]).
   3. Perform a logical operation to determine if the values in the matrix from step 1b are greater than 0. If they are greater than 0 then the value is assigned a 1 and if not then the value is assigned a 0 (resulting in a logical matrix that represents if the volunteer experienced any symptoms on each given timepoint [volunteer x timepoint]).
2. Calculate the cumulative number of days a participant experienced symptoms.
   1. Take the logical matrix from step 1c and take the cumulative sum across time from baseline to the given timepoint for each timepoint (resulting in a matrix representing the number of days that a volunteer experienced symptoms up to each timepoint [volunteer x timepoint]).
3. Summarise across the quarantine phase.
   1. Take the values from the final timepoint of quarantine in the matrix from step 2a (resulting in a vector of values that represent the total days a participant experienced symptoms over the quarantine phase).
   2. Concatenate the values from step 3a with the follow up timepoints from the matrix from step 2a (resulting in a [volunteer x timepoint] matrix where the first column is the total number of days a volunteer experienced symptoms over the quarantine timepoints and the subsequent columns are the total number of days a volunteer experienced symptoms up to that timepoint).

## 2.7 - University of Pennsylvania Smell Identification Test (UPSIT)

The UPSIT data were processed in the same way as the temperature data in section 2.5. This produced maximum change from baseline UPSIT scores to take forward for analysis.

## 2.8 - Plasma Markers of Brain Injury

The plasma markers of brain injury were prepared for analysis in the following way:

1. Normalisation within brain injury marker
   1. Starting with a matrix of plasma concentrations [volunteer x brain injury marker x timepoint].
   2. Take the mean across volunteers of the baseline timepoint (resulting in a mean baseline value for each brain injury marker).
   3. Take the standard deviation across volunteers of the baseline timepoint (resulting in a baseline standard deviation value for each brain injury marker).
   4. Subtract the result of step 1b from the matrix from step 1a on each time point (resulting in a mean corrected set of values for each brain injury marker, volunteer and timepoint [volunteer x brain injury marker x timepoint]).
   5. Divide through by the values calculated in step 1c to fully normalise the data (resulting in a matrix of plasma concentrations of brain injury markers normalised by the mean and standard deviation of the baseline for the corresponding brain injury marker [volunteer x brain injury marker x timepoint]).
2. Baseline correction
   1. The value of the baseline timepoint from step 1e for each brain injury marker was subtracted from each subsequent timepoint within corresponding brain injury marker (resulting in a baseline corrected normalised matrix of brain injury marker plasma concentrations [volunteer x brain injury marker x timepoint]).

# 3 - Main Analyses

## 3.1 - Main Group Difference in bcGCCS

The main outcome analysis was designed to look at the group difference over time between infected and uninfected individuals in the bcGCCS. In order to investigate this the following procedure was taken:

1. Start with the matrix from step 5b of section 2.1
2. A repeated measures analysis of variance was conducted using phase as the within subjects factor and the group variable as the between subjects factor. The matlab functions fitrm, anova and ranova were used.
3. Two sample, two tailed, uncorrected T-Tests were performed at each phase between the infected and uninfected groups. The matlab function fitlm was used with the binary group categorical used to predict the cognitive scores (equivalent to a T-Test).

## 3.2 - Group Differences in Individual Tasks

The secondary analysis was designed to look at more detail in to the nature of the cognitive deficits observed in the main analysis. This was split in to two sections, one looking at all of the individual tasks, and one looking solely at the task with the greatest effect size, the object memory task.

### 3.2.1 - All individual tasks

The steps taken for this analysis were as follows:

1. Start with the matrix from step 2b of section 2.2.
2. Take the mean across all timepoints to create a summary of the whole time course of the experiment for each volunteer and task.
3. Perform Two sample, Two tailed, uncorrected T-Tests of cognitive scores at each phase of the matrix from step 1 and the mean of the phases calculated in step 2.

### 3.2.2 - Object Memory Task

The object memory task had the highest effect size between the groups so it was deemed worthwhile to look at scores beyond the main outcome measure for this task alone. The extra scores that were investigated were processed in the same way as the primary outcome measures for the other tasks as can be seen in section 2.2. Then the following steps were taken:

1. A repeated measures anova was performed using phase as the within subjects factor and group as the between subjects factor for each of the 3 extra scores for the immediate and delayed task.

# 4 - Sensitivity Analyses

There were a number of quirks of the study design and outcome that may have affected the main results. These included:

1. Learning in the cognitive scores.
2. Which baseline day was used to baseline correct the data.
3. Including or excluding 2 volunteers that completed the study but were found to have evidence of prior COVID infection between screening and starting the study.
4. The effect of volunteers getting upper respiratory tract infections (including COVID-19) in the community between quarantine and follow-up timepoints.
5. The effect of Remdesivir that was given to some of the initial study volunteers.

This section will describe the statistical processes by which the effects of these factors were investigated.

## 4.1 - Learning Effects

The effect of learning was investigated by performing the main analysis described in section 3.1 but starting with the learning and non-learning composites described in section 2.3.

## 4.2 - Baseline Day

The effect of which baseline day was investigated by repeating the main analysis in section 3.1 but starting by selectively using baseline day 1 OR baseline day 2 (rather than the mean of the two) when calculating the bcGCCS described in section 2.1.

## 4.3 - Including excluded volunteers

The effect of including the excluded volunteers was investigated by simply repeating the main analysis in section 3.1 but starting with data that include the 2 excluded volunteers when calculating the bcGCCS.

## 4.4 - Community infection and Remdesivir

Investigating the effect of community infection was more complicated because it is a time varying covariate. In order to investigate its effect on the results we calculated a linear mixed effects model. It included timepoint, remdesivir treatment, lab infection group and community infection as fixed effects along with a community infection by lab infection interaction and subject as a random effect. We used the matlab function fitlme.

This was also repeated comparing every combination of the factors from section 4.1, 4.2 and 4.3.

# 5 - Exploratory Analyses

A number of exploratory analyses were conducted to look at the relationship between the main cognitive finding and other physiological measurements. These measurements included:

1. Viral load
2. Temperature
3. Symptom load
4. UPSIT score
5. Plasma markers of brain injury

## 5.1 - Viral Load, Temperature, Symptom Load and UPSIT scores

These 4 variables were analysed in relation to the cognitive data by performing spearman’s rank correlations with the cognitive data in the infected group only at each phase of the study.

## 5.2 - Plasma Markers of Brain Injury

The plasma markers of brain injury were analysed in two ways:

1. Repeated measures ANOVA of brain injury markers across the phases of the trial.
2. Spearman’s rank correlation within the infected group for each marker and the bcGCCS at each phase of the trial.

## 
